# Differential effects of electroacupuncture and manual acupuncture on spontaneously hypertensive rats: insights from intestinal microbiota and metabolomics

**DOI:** 10.3389/fmolb.2025.1619356

**Published:** 2025-07-08

**Authors:** Ji-Peng Liu, Long-Teng Tu, Ke-Zhen Yang, Yin-Yin Li, Yu Gong, Bing-Xuan Han, Chuan Liu, Tian-Qi Xia, Yu Liu, Xiao-Min Hao, Bing-Nan Yue, Jing Zhang, Bing-Hui Wang, Gui-Rong Luo, Qing-Guo Liu, Meng Xu

**Affiliations:** ^1^ School of Acupuncture-Moxibustion and Tuina, Beijing University of Chinese Medicine, Beijing, China; ^2^ Department of Rehabilitation Medicine, Sir Run Run Shaw Hospital, Zhejiang University School of Medicine, Hangzhou, Zhejiang, China; ^3^ Department Shenzhen Hospital, Beijing University of Chinese Medicine, Shenzhen, China; ^4^ Department of Traditional Chinese Medicine, Wangjing Community Health Center, Beijing, China; ^5^ College of Special Education, Beijing Union University, Beijing, China; ^6^ Department of Tuina, Beijing University of Chinese Medicine Third Affiliated Hospital, Beijing, China

**Keywords:** hypertension, electroacupuncture, manual acupuncture, spontaneously hypertensive rats, gut flora, metabolites

## Abstract

**Background:**

Hypertension is a significant risk factor for cardiovascular disease, and acupuncture has demonstrated therapeutic effects in managing hypertension. However, the precise antihypertensive mechanisms of acupuncture require further elucidation.

**Methods:**

In this study, 30 male spontaneously hypertensive rats (SHRs) and 10 male Wistar Kyoto (WKY) rats were utilized as experimental models. The SHRs were randomly assigned to three groups: the model group (Group M), the electroacupuncture group (Group EA), and the manual acupuncture group (Group MA), while the WKY rats served as the blank control group. Treatment was given every other day for 8 weeks, and systolic and diastolic blood pressures were measured every 2 weeks during the intervention period. Upon completion of the intervention, analyses of intestinal flora, as well as serum and fecal metabolomics, were conducted.

**Results:**

The findings indicated that both EA and MA effectively reduced systolic and diastolic blood pressure in SHRs, with EA demonstrating a more rapid onset of blood pressure reduction. EA and MA influence the composition of intestinal microbiota in SHRs, aligning the microbial structure more closely with that of the WKY group. This modulation results in an increased abundance of beneficial bacteria, such as Blautia, and a decreased abundance of harmful bacteria, such as *Helicobacter*. Regarding serum metabolomics, EA and MA affect metabolic pathways involving glycerophospholipids, linoleic acid, and arachidonic acid. In terms of fecal metabolomics, both acupuncture techniques are implicated in primary bile acid biosynthesis, dopaminergic synapse function, and sphingolipid signaling pathways. Notably, EA exerts a more significant influence on the steroid hormone biosynthesis pathway, whereas MA impacts the tryptophan metabolic pathway.

**Conclusion:**

Both EA and MA demonstrate antihypertensive effects by modulating intestinal microbiota composition and metabolite profiles in SHRs, although the specific microbiota and metabolites affected differ between the two techniques, and EA treatment reversed more fecal and serum metabolites than did MA. This study serves as a reference for investigating the mechanisms underlying acupuncture in the treatment of hypertension and facilitates its clinical application.

## 1 Introduction

Hypertension is a significant risk factor for ischemic heart disease, stroke, other cardiovascular diseases, chronic kidney disease, and dementia (Fuchs and Whelton, 2020; Suvila and Niiranen, 2022; Zhou et al., 2021a; Zhou et al., 2021b). Presently, over one billion adults globally are affected by hypertension, and its prevalence, along with the associated socioeconomic burden, continues to escalate due to population aging and lifestyle modifications. The control rate of hypertension remains suboptimal (Zhou et al., 2021b). Despite advancements in the combined use of antihypertensive medications and the optimization of drug dosages, the stabilization of hypertension control remains inadequate (Richards et al., 2022). Furthermore, current oral antihypertensive medications may lead to adverse effects such as electrolyte imbalances, renal impairment, and hyperkalemia (Gao et al., 2021; Whelton et al., 2018). Acupuncture is being explored as a green, safe complementary alternative therapy (Zhang B et al., 2022). Emerging evidence indicates that it demonstrates superior clinical efficacy in antihypertensive therapy and significantly contributes to the protection of target organs (Niu et al., 2019; Zhang et al., 2021; Zhang M et al., 2022; Zheng et al., 2019).

The pathogenesis of hypertension is intricately associated with gut microbiota (Howitt and Garrett, 2012; Tang et al., 2013; Yang et al., 2018), with dysbiosis potentially leading to elevated blood pressure and subsequent damage to the heart, vasculature, and kidneys (Avery et al., 2023). The composition of the gut microbiome and the integrity of the intestinal barrier are critical determinants in the progression of hypertension (Yang et al., 2024). An imbalance in intestinal flora induces intestinal inflammation, leading to dysfunctions in both the sympathetic nervous system and intestinal function (Yang et al., 2015). This imbalance compromises the intestinal barrier, decreases mechanotransduction function, and increases permeability, allowing intestinal pathogenic bacteria and enterotoxins to enter the bloodstream. This penetration triggers a cascade of inflammatory responses, impairing vascular endothelial function and contributing to vascular sclerosis, which subsequently elevates blood pressure and facilitates the onset of hypertension (Avery et al., 2021; Santisteban et al., 2017). Acupuncture has been shown to positively influence the intestinal microbial community, effectively modulating the composition of intestinal microorganisms and optimizing the intestinal environment (Jang et al., 2020; Lv et al., 2022; Yang L et al., 2022). Furthermore, acupuncture has been observed to reduce blood pressure by modulating dysregulated intestinal flora in hypertensive patients, although the precise mechanisms underlying this effect remain unclear (Wang J. et al., 2021).

Metabolomics has been extensively employed to elucidate disruptions in metabolic pathways across a spectrum of diseases, including hypertension (Sinclair et al., 2021). Recent serum metabolomics investigations in SHRs have uncovered the presence of several metabolic abnormalities, encompassing lipid metabolism, amino acid metabolism, the tricarboxylic acid cycle, urea metabolism, and the 5-hydroxytryptamine synaptic transduction pathway within hypertensive pathology (Tian et al., 2018; Wang et al., 2020). Metabolites present in feces represent the products of the combined metabolic activities of the intestinal microbiota and the host, and they can also serve as indicators of metabolic status under pathological conditions (Zeng et al., 2015). Interactions between the host and microbiota, particularly those involving inflammatory and metabolic pathways, have been implicated in the etiology of cardiovascular disease (Tang et al., 2017; Witkowski et al., 2020). Metabolites derived from intestinal flora have been linked to the regulation of blood pressure, with their concentrations correlating with the progression of hypertension. These metabolites primarily include short-chain fatty acids (SCFAs), trimethylamine-N-oxide (TMAO), lipopolysaccharides, and bile acids (Verhaar et al., 2020). In recent years, the modification of intestinal microbiota by Traditional Chinese Medicine (TCM) and the targeting of these microbiota for disease treatment have emerged as significant advancements in the life sciences (Li et al., 2021). Notably, acupuncture has been shown to enhance clinical pathology by modulating and restoring the body’s metabolomic network (Chen et al., 2022; Jung et al., 2021).

Preliminary metabolomics research conducted by our team has demonstrated that acupuncture at the Zusanli (ST36) and Taichong (LR3) points in SHRs can regulate metabolic pathways, including primary bile acid biosynthesis, tryptophan metabolism, and bile secretion, thereby influencing the renal cortex and exerting antihypertensive effects (Yang L et al., 2022; Yang et al., 2022b; Zhang, et al., 2022). Furthermore, studies have identified differences between EA and MA concerning afferent pathways, mechanisms of action, targets, and clinical efficacy (Johansson et al., 2013; Stener-Victorin et al., 2006; Zhao, 2008). To our knowledge, there is a paucity of studies investigating the antihypertensive mechanisms of EA and MA in hypertension with metabolomics and gut microbiota analysis techniques. Consequently, SHRs were chosen as the experimental model for this study. The research involved analyzing the intestinal microbiota, serum, and fecal metabolites utilizing 16S rDNA sequencing and liquid chromatography-mass spectrometry (LC-MS) non-targeted metabolomics. The objective was to examine the effects of EA and MA on the intestinal microbiota and metabolites of SHRs, as well as to compare the similarities and differences between these two acupuncture modalities. Ultimately, this study aims to elucidate the role of intestinal microbiota and metabolites in the acupuncture-based treatment of hypertension.

## 2 Materials and methods

### 2.1 Animals

A total of 30 male SHRs (aged 14 weeks, weighing 280 ± 20 g) and 10 male Wistar Kyoto (WKY) rats (aged 14 weeks, weighing 280 ± 20 g) were procured from Beijing Vital River Laboratory Animal Technology Co., Ltd. [License No. SCXK (Jing) 2016-0006]. The animals were maintained in a specific pathogen-free (SPF) facility at Beijing University of Chinese Medicine under controlled environmental conditions: a temperature of 24°C ± 1°C, humidity at 55% ± 5%, and a 12-h light/dark cycle with lights on at 8:00 a.m. Food and water were provided *ad libitum* throughout the experimental period.

### 2.2 Experimental Procedures

**FIGURE 1:**
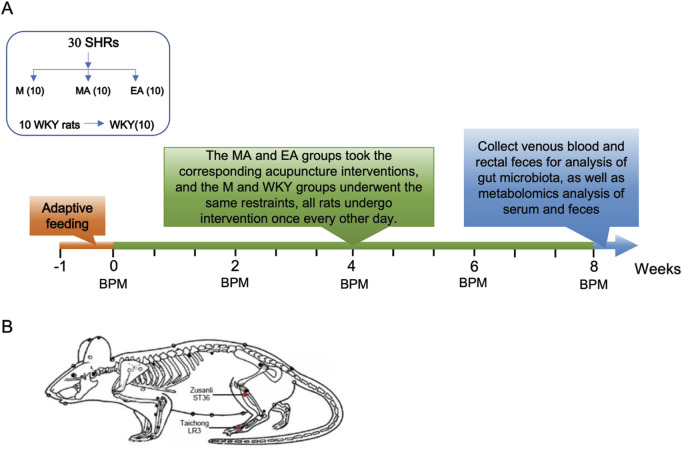
Experimental Procedures and Acupoint Selection. **(A)** Experimental flowchart. **(B)** Treatment acupoint diagram. Red dots indicate the location of Zusanli (ST36), Taichong (LR3).

The experimental design is depicted in Figure 1A. Following a 1-week acclimatization period, the 30 SHRs were randomly assigned into three groups (n = 10 per group): the M group (control), the EA group (electroacupuncture stimulation), and the MA group (manual acupuncture stimulation). Ten age-matched WKY rats served as the normotensive control group. All rats underwent interventions on alternate days for a duration of 8 weeks, with blood pressure measurements conducted biweekly. At the conclusion of the 8-week intervention period, eight rats were randomly selected from each group, and after anesthesia, blood was taken from the abdominal aorta, and feces were taken from the rectum. Serum and fecal metabolomic analyses were conducted, and the fecal samples underwent additional examination for gut microbiota composition.

### 2.3 Blood pressure measurement

Blood pressure measurements were conducted by two technicians between 8:00 and 11:00 a.m. on the 0th, 14th, 28th, 42nd, and 56th days of the intervention cycle. A small animal blood pressure monitoring device (Beijing Softron Technology Development Co., Ltd., Beijing, China) was employed to assess blood pressure in the tail artery of unanesthetized rats. The rats were placed into the temperature-controlled sleeve of the device for a preheating period of 10 min, with the thermostat set to 36°C. Once the rats were calm and acclimatized, their tails were inserted into the ring-shaped blood pressure measurement receptors. Blood pressure was recorded three times for each rat, and the mean of these three measurements was calculated to obtain the final blood pressure value.

### 2.4 Interventional methods

Following the measurement of baseline blood pressure, all unanaesthetized rats were secured on the rat plate. Identical acupoints were selected for both the EA and MA groups, specifically LR3 (located anteriorly in the depression between the first and second metatarsal bones) and ST36 (situated 2 mm lateral to the anterior tibial tuberosity and 5 mm below the fibular head beneath the knee), as illustrated in Figure 1B. A disposable sterile stainless-steel needle (0.18 mm × 13 mm) (Beijing Zhongyan Taihe Medical Instrument Co., Ltd., Beijing, China) was inserted bilaterally into the LR3 of the rats at a depth of 2 mm and an angle of 30°, while the ST36 was punctured vertically to a depth of 5 mm. In the EA group, the acupoints were connected to the Korean Acupuncture Nerve Stimulation Instrument (Nanjing Jisheng Medical Science and Technology Co., Ltd., Jiangsu, China) to administer electrical stimulation with an intensity of 1 mA at a frequency of 2 Hz. In contrast, the MA group received manual stimulation, wherein each acupuncture needle was rotated bi-directionally within a 90-degree range at a speed of 180° per second, performed every 5 min for a duration of 15 s. The EA and MA groups received treatment for 20 min per session, while the M and WKY groups did not receive treatment; however, they underwent the same grasping and fixation stimulation for 20 min per session. These interventions were administered every other day between 13:00 and 16:00, over a period of 8 weeks.

### 2.5 Metabolomic analysis

#### 2.5.1 Metabolite extraction

Eight rats were randomly selected from each group. After anesthetizing the rats, the abdominal cavity was surgically opened, and blood was collected from the abdominal aorta. A volume of 100 μL of the collected blood was transferred into a 1.5 mL centrifuge tube, followed by the addition of 400 μL of an extraction solution composed of acetonitrile and methanol in a 1:1 ratio. This solution contained four internal standards, including L-2-chlorophenylalanine and its three isotopes at a concentration of 0.02 mg/mL. The mixture was subjected to vortex mixing for 30 s and subsequently underwent low-temperature ultrasonic extraction at 5°C and 40 KHz for 30 min. The samples were then allowed to stand at −20°C for 30 min. Centrifugation was conducted at 4°C for 15 min at 13,000 g. The supernatant was carefully removed and evaporated to dryness under a stream of nitrogen gas. The residue was reconstituted in 100 µL of a re-solution consisting of acetonitrile and water in a 1:1 ratio. This reconstituted solution was subjected to low-temperature ultrasonic extraction for 5 min at 5°C and 40 kHz, followed by centrifugation at 13,000 g for 10 min at 4°C. The resulting supernatant was transferred to a vial equipped with an internal cannula for subsequent analysis.

The mid-rectal feces were collected from rats after blood sampling, and 100 mg of solid sample was taken in a 2 mL centrifuge tube, to which a 6 mm diameter grinding bead was added. Metabolite extraction was conducted using 800 μL of an extraction solution composed of methanol and water in a 4:1 volume ratio, which included four internal standards such as L-2-chlorophenylalanine and its three isotopes at a concentration of 0.02 mg/mL. The sample solutions were subjected to grinding using a frozen tissue grinder for 6 min at −10°C and 50 Hz, followed by low-temperature ultrasonic extraction for 30 min at 5°C and 40 kHz. Subsequently, the samples were allowed to stand at −20°C for 30 min, centrifuged at 4°C and 13,000 g for 15 min, and the resulting supernatant was transferred into a vial equipped with an internal cannula for further analysis.

#### 2.5.2 Quality control

A 20ul equivalent volume of each sample metabolite was combined to create a quality control (QC) sample. During instrumental analysis, a QC sample was interspersed every 5 to 10 samples to assess the reproducibility of the entire analytical procedure.

#### 2.5.3 LC-MS analysis

The LC-MS analysis was conducted utilizing an ultra-high performance liquid chromatography tandem Fourier transform mass spectrometry system, specifically the UHPLC-Q Exactive HF-X model. A 3 μL aliquot of the sample was separated on an HSS T3 column (dimensions: 100 mm × 2.1 mm i.d., 1.8 µm particle size) prior to mass spectrometric detection. The mobile phase A comprised a solution of 0.1% formic acid in a water/acetonitrile mixture (95/5, v/v), while mobile phase B consisted of an acetonitrile/isopropanol/water mixture (47.5/47.5/5, v/v/v) with 0.1% formic acid. The chromatographic separation was carried out at a flow rate of 0.40 mL/min and a column temperature of 40°C. Mass spectrometric data acquisition was executed in both positive and negative ion scanning modes, covering a mass-to-charge ratio (m/z) range of 70–1050. The ion spray voltages were set at 3500 V for positive ions and −3000 V for negative ions. Additional parameters included a sheath gas flow rate of 50 arbitrary units, an auxiliary heated gas flow rate of 13 arbitrary units, an ion source heating temperature of 450°C, and cyclic collision energies of 20, 40, and 60 V.

#### 2.5.4 Data processing

Following on-boarding, LC-MS raw data were processed using Progenesis QI (Waters Corporation, Milford, United States) for baseline filtering, peak identification, integration, retention time correction, and alignment, resulting in a data matrix comprising retention time, mass-to-charge ratio, and peak intensities. Concurrently, MS and MSMS data were matched with public databases HMDB (http://www.hmdb.ca/), Metlin (https://metlin.scripps.edu/), and the Majorbio Database (MJDB) from Majorbio Biotechnology Co., Ltd. (Shanghai, China).

The data matrix, following the library search, was uploaded to the Majorbio cloud platform (https://cloud.majorbio.com) for further analysis. Initial pre-processing of the data matrix involved applying the 80% rule to address missing values; specifically, variables with more than 80% non-zero values in at least one set of samples were retained. Subsequently, missing values were imputed using the smallest values present in the original matrix. To mitigate errors arising from sample preparation and instrument instability, the response intensities of the sample mass spectrometry peaks were normalized using the sum-normalization method, resulting in normalized data matrices. Concurrently, variables exhibiting a relative standard deviation (RSD) greater than 30% in quality control (QC) samples were excluded. The remaining data were then subjected to log10 transformation to produce the final data matrix for subsequent analysis.

### 2.6 Intestinal flora analysis

#### 2.6.1 Sample DNA extraction

Genomic DNA was extracted from microbial communities utilizing the E.Z.N.A.® Soil DNA Kit (Omega Bio-tek, Norcross, GA, United States) following the manufacturer’s protocol. The integrity of the extracted DNA was assessed via agarose gel electrophoresis using a 1% agarose gel. DNA concentration and purity were quantified with a NanoDrop 2000 spectrophotometer (Thermo Scientific, United States).

#### 2.6.2 PCR amplification and sequencing library construction

The extracted DNA was employed as a template for the polymerase chain reaction (PCR) amplification of the V3-V4 variable region of the 16S rRNA gene, utilizing the primer pairs 338F (5′-ACTCCTACGGGGAGGCAGCAG-3′) and 806R (5′-GGACTACHVGGGTWTCTAAT-3′) (Liu et al., 2016). These primers incorporate a Barcode sequence. The PCR reaction mixture consisted of 4 μL of 5× TransStart FastPfu buffer, 2 μL of 2.5 mM deoxynucleotide triphosphates (dNTPs), 0.8 μL of each primer (5 μM), 0.4 μL of FastPfu polymerase, 10 ng of template DNA, and double-distilled water (ddH2O) to achieve a final volume of 20 μL. The amplification protocol included an initial denaturation at 95°C for 3 min, followed by 27 cycles of denaturation at 95°C for 30 s, annealing at 55°C for 30 s, and extension at 72°C for 30 s, with a final extension at 72°C for 10 min. The PCR products were subsequently stored at 4°C. The amplification was conducted using an ABI GeneAmp® 9,700 thermal cycler. The PCR products were resolved on a 2% agarose gel, purified using a DNA gel recovery and purification kit (PCR Clean-Up Kit, China), and quantified using a Qubit 4.0 fluorometer (Thermo Fisher Scientific, United States).

The purified PCR products were utilized for library construction employing the NEXTFLEX Rapid DNA-Seq Kit, following a series of steps: (1) junction linkage, (2) elimination of self-ligated junction fragments through magnetic bead screening, (3) enrichment of the library template via PCR amplification, and (4) recovery of PCR products using magnetic beads to obtain the final library. Sequencing was conducted on the Illumina Nextseq2000 platform in accordance with the standard protocols provided by Majorbio Bio-Pharm Technology Co. Ltd. (Shanghai, China).

#### 2.6.3 High-throughput sequencing data analysis

The paired-end raw sequencing reads were subjected to quality control using the fastp software (https://github.com/OpenGene/fastp, version 0.19.6) and FLASH software (http://www.cbcb.umd.edu/software/flash, version 1.2.11) for read merging. The quality control process involved the following steps: (1) Bases with a quality score of 20 or lower at the ends of the reads were filtered out. A sliding window of 10 bp was applied, and if the average quality score within this window was below 20, the bases from the window onwards were truncated. Reads with a quality score of 50 bp or lower after quality control were discarded, as were reads containing ambiguous ‘N' bases. (2) Based on the overlap between paired-end reads, pairs of reads were merged into a single sequence, requiring a minimum overlap length of 10 bp. (3) The maximum allowable mismatch ratio in the overlap region of a merged sequence was set at 0.2, and sequences not meeting this criterion were excluded. (4) Samples were differentiated by barcodes and primers located at the beginning and end of the sequences, with sequence orientation adjusted accordingly. The barcode allowed for zero mismatches, while the maximum number of allowable primer mismatches was set at two. Chimeric sequences were eliminated, and Operational Taxonomic Units (OTUs) were clustered at a 97% similarity threshold utilizing UPARSE (http://drive5.com/uparse/, version 11.0.667). Sequences identified as having chloroplast or mitochondrial origins were excluded, as recommended in cases of contamination. To mitigate the effects of sequencing depth on alpha and beta diversity analyses, all samples underwent rarefaction to 20,000 sequences per sample, a recommended practice, resulting in an average Good’s coverage of 99.09% following normalization. OTU taxonomic annotation was conducted using the Silva 16S rRNA database (version 138.2) with the RDP classifier (http://rdp.cme.msu.edu/, version 2.11) at a 70% confidence threshold. Subsequent community composition analysis was performed across multiple taxonomic levels. Functional prediction of the 16S data was carried out using PICRUSt2 (version 2.2.0).

### 2.7 Statistical analysis

The experimental data obtained were analyzed utilizing SPSS version 20.0 software (IBM, Armonk, NY). Initially, the data underwent normality testing, followed by an assessment of homogeneity. The systolic and diastolic blood pressure measurements in rats were subsequently analyzed using a two-factor repeated measures ANOVA, with statistical significance set at *p* < 0.05. Data visualization, including line and bar graphs, was performed using GraphPad Prism version 8 (GraphPad Software, San Diego, CA). Metabolomics and gut microbiota data analyses were conducted using the Majorbio Cloud platform (https://cloud.majorbio.com) as detailed below: The mothur software (http://www.mothur.org/wiki/Calculators) was employed to compute the Alpha diversity indices, including Chao 1 and Shannon. The Wilcoxon rank-sum test was utilized to assess intergroup differences in Alpha diversity. The similarity of microbial community structures between samples was evaluated using Principal Coordinate Analysis (PCoA) based on the Bray-Curtis distance algorithm, in conjunction with the PERMANOVA nonparametric test, to determine the significance of differences in microbial community structures between sample groups. Additionally, differences in microbial community structures between sample groups were analyzed using LEfSe (Linear Discriminant Analysis Effect Size) (http://huttenhower.sph.harvard.edu/LEfSe), with criteria set at LDA >1 and *p* < 0.05, to identify bacterial taxa exhibiting significant differences in abundance at the genus level between groups.

## 3 Results

### 3.1 Effects of EA and MA on blood pressure in SHRs

**FIGURE 2:**
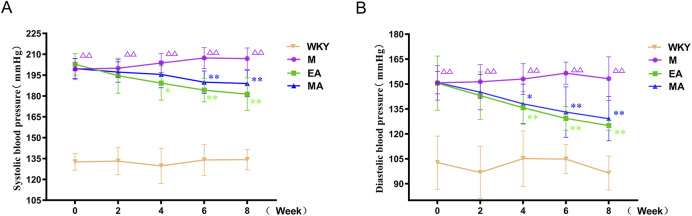
Blood Pressure of Rats in Each Group. **(A)** Systolic blood pressure **(B)** Diastolic blood pressure. Values are expressed as Means ± SD (n = 10 per group). ^ΔΔ^ *p* < 0.01 vs. the WKY group; *p < 0.05 and **p < 0.01 vs. the M group. WKY, normal control group; M, model group; EA, electroacupuncture group; MA, manual acupuncture group.

As illustrated in Figure 2, the analysis of rat systolic blood pressure data revealed significant treatment effects (F = 517.323, *p* < 0.01), time effects (F = 2.599, *p* < 0.01), and a significant interaction between treatment and time (F = 3.88, *p* < 0.01). Similarly, the diastolic blood pressure data demonstrated significant treatment effects (F = 97.645, *p* < 0.01), time effects (F = 7.508, *p* < 0.01), and a significant interaction between treatment and time (F = 3.456, *p* < 0.01). These findings indicate a difference in both systolic and diastolic blood pressure across different groups and time periods, suggesting that the blood pressure of SHRs varied with acupuncture treatment. In comparison to the WKY group, the systolic and diastolic blood pressures in the M group exhibited a gradual increase and stabilized at elevated levels during weeks 0–8 of the intervention (*p* < 0.01). Relative to the M group, systolic blood pressure began to decrease in the EA and MA groups at weeks 4 and 6 of measurement, respectively (*p* < 0.05, *p* < 0.01). Furthermore, compared to the M group, the systolic blood pressure in the EA and MA groups consistently decreased from week 6 to week 8 of the intervention (both *p* < 0.01). Diastolic blood pressure demonstrated a significant reduction in the EA and MA groups starting at week 4 of measurement compared to the M group (*p* < 0.01, *p* < 0.05). Furthermore, a consistent decrease in diastolic blood pressure was observed from week 6 to week 8 of the intervention in the EA and MA groups relative to the M group (both *p* < 0.01). The findings from these studies indicate that both EA and MA are effective in reducing systolic and diastolic blood pressure in SHRs. Moreover, the efficacy of acupuncture in lowering blood pressure appears to have a cumulative effect over time. The analysis also revealed that EA induces a more rapid onset of blood pressure reduction compared to MA.

### 3.2 Effects of EA and MA on intestinal flora in SHRs

#### 3.2.1 Analysis of the diversity of rat intestinal flora in each group

**FIGURE 3:**
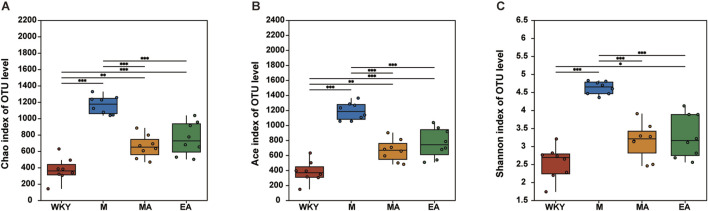
Alpha Diversity Analysis. **(A)** Chao index. **(B)** Ace index. **(C)** Shannon index. The data are presented as the mean ± SD, n = 8. *p < 0.05; **p < 0.01; ***p < 0.001. WKY, normal control group; M, model group; EA, electroacupuncture group; MA, manual acupuncture group.

Following the acquisition of the OTUs abundance array, the gut microbiota of all rats was subjected to analysis for both Alpha and Beta diversity. The richness and diversity of the rat gut microbiota composition were evaluated using three principal ecological indices: ACE, Chao, and Shannon. Notably, the ACE and Chao indices exhibited a positive correlation with microbial species richness, while the Shannon index was positively correlated with microbial species diversity. Our findings revealed that the ACE, Chao, and Shannon indices were significantly elevated in the M group compared to the WKY group (*p* < 0.001), indicating a greater diversity and abundance of gut microbiota in the M group. Conversely, the diversity and abundance of the gut microbiota were markedly diminished in the EA and MA groups relative to the M group (*p* < 0.001), as illustrated in Figure 3.

**FIGURE 4:**
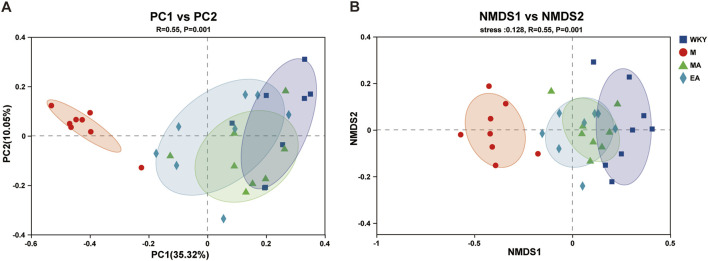
Beta Diversity Analysis. **(A)** Principal Coordinate Analysis (PCOA) is used to investigate the community similarity of the gut microbiota. Each point in the graph represents one sample. The closer the distance between two points, the higher the similarity of gut microbiota composition. **(B)** Nonmetric multidimensional scaling analysis (NMDS) was used to compare differences between sample groups. The closer the distance between two points, the smaller the difference in gut microbiota composition. WKY, normal control group; M, model group; EA, electroacupuncture group; MA, manual acupuncture group.

In this study, principal coordinate analysis (PCoA) and non-metric multidimensional scaling (NMDS) were conducted utilizing the Bray-Curtis distance metric. These analytical methods effectively assessed alterations in gut microbiota structure across samples, with inter-sample distances elucidating the similarities and differences in community composition. The findings reveal that the majority of samples from the WKY and M groups were distinctly separated, indicating substantial differences in gut microbiota composition between these two groups. Conversely, the relatively small distances among samples from the WKY, MA, and EA groups suggest a high degree of similarity in microbial composition within these groups (ANOSIM, R = 0.55, *p* = 0.001). These results indicate that the gut microbiota in the EA and MA groups underwent modifications post-treatment, aligning more closely with the microbiota composition observed in the WKY group, as illustrated in Figure 4.

#### 3.2.2 Analysis of relative abundance of rat intestinal flora in each group

**FIGURE 5:**
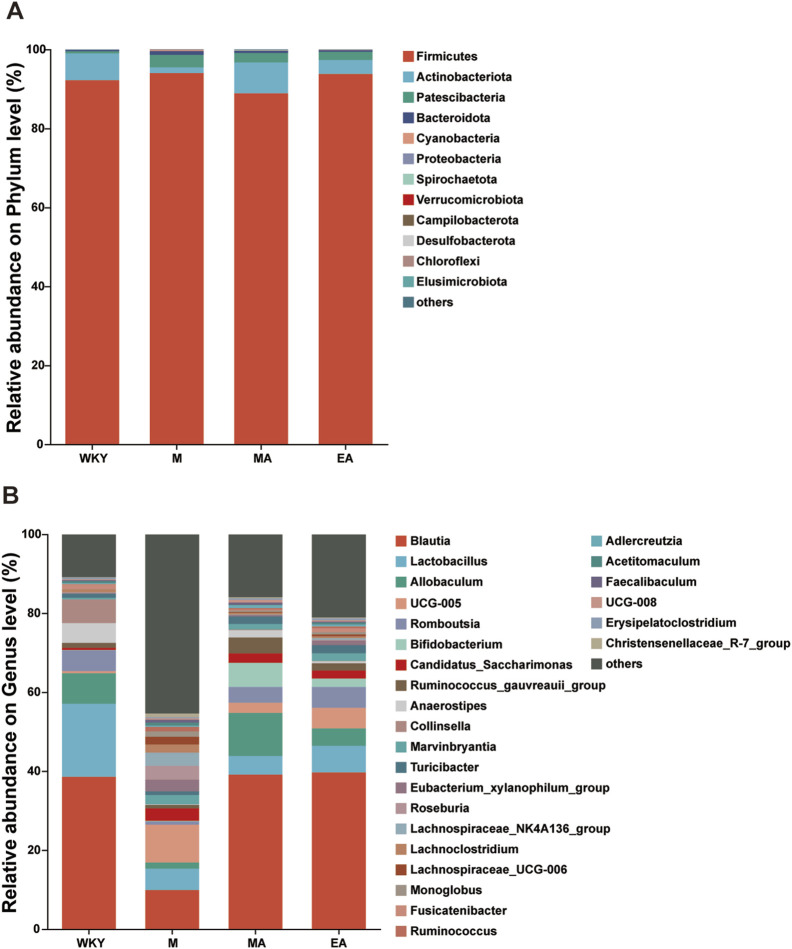
Analysis of the Composition of the Intestinal Flora in Each Group of Rats. **(A)** For phylum level flora. **(B)** Genus level flora. Horizontal coordinates are the sample name, vertical coordinates are the proportion of the species in that sample, different colored bars represent different species, and the length of the bar represents the size of the proportion of the species. WKY, normal control group; M, model group; EA, electroacupuncture group; MA, manual acupuncture group.

As illustrated in Figure 5, the bar charts depict the average abundance of highly prevalent abundance at both the phylum and genus levels for samples from the WKY, M, MA, and EA groups. The findings indicate that the predominant phyla in rat fecal samples from the WKY, MA, and EA groups were Firmicutes and Actinobacteriota, whereas the M group was primarily dominated by Firmicutes and Patescibacteria. At the genus level, the samples across all groups were chiefly dominated by Blautia, *Lactobacillus*, Allobaculum, UCG-005, and Romboutsia.

#### 3.2.3 Analysis of iconic rat intestinal flora in each group

**FIGURE 6:**
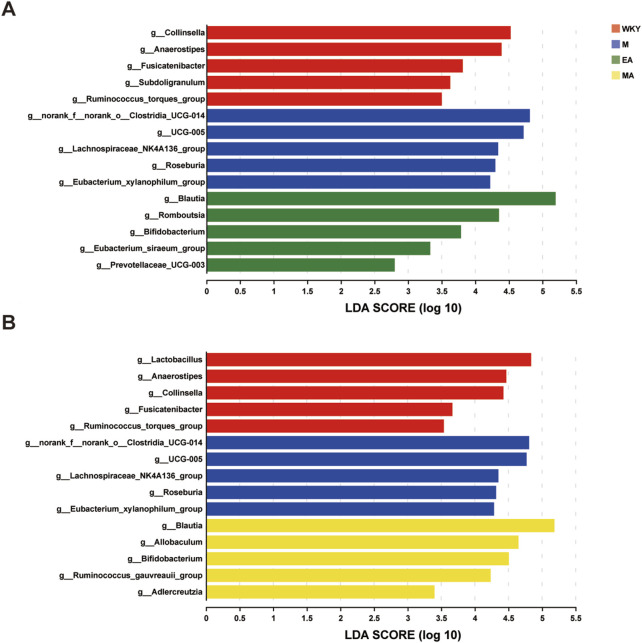
LEfSe Analysis of Differential Flora. **(A)** Bacterial genera with significant difference in abundance among WKY, M and EA groups. **(B)** Bacterial genera with significant difference in abundance among WKY, M and MA groups. The LDA discriminant bar chart counts the microbial taxa with significant effects in multiple groups, and the LDA scores obtained by LDA analysis (linear regression analysis); the larger the LDA score, the greater the effect of species abundance on the differential effect. WKY, normal control group; M, model group; EA, electroacupuncture group; MA, manual acupuncture group.

LEfSe analyses were conducted to identify key species that exhibited significant differences between groups, and a Linear Discriminant Analysis (LDA) score was calculated to distinguish signature microorganisms within each group that significantly influenced variations in colony structure. The findings indicated that microorganisms such as Collinsella, Anaerostipes, and Fusicatenibacter exerted a substantial influence on the structural differences of the bacterial flora in the normotensive control group. In group M, UCG-005 and Roseburia were identified as the signature microorganisms affecting the flora structure in this cohort of rats. In the MA group, the dominant bacteria that significantly impacted the differences in colony structure included Blautia, Allobaculum, Bifidobacterium, and Ruminococcus, among others. Similarly, in the EA group, Blautia, Romboutsia, and Bifidobacterium were the dominant bacteria influencing the structural differences in the flora, as illustrated in Figure 6.

#### 3.2.4 Analysis of the intestinal flora of differences between groups

In comparison to the WKY group, the M group exhibited a significantly reduced abundance of Blautia and *Lactobacillus*, alongside a significantly increased abundance of UCG-005, *Bacteroides*, and *Helicobacter*. Conversely, the EA and MA groups demonstrated a significantly higher abundance of Blautia, Bifidobacterium, and Romboutsia, as well as a significantly lower abundance of UCG-005, *Bacteroides*, and *Helicobacter*, relative to the M group. These findings suggest that both EA and MA have the potential to ameliorate gut flora disturbances in SHRs and restore balance to the gut microbiota. For detailed data, refer to Table 1 and Supplementary Tables S1-S3.

**TABLE 1:** The abundance of the six most abundant genera in the gut microbiota in rats.

Genus	WKY	M	MA	EA
Blautia (%)	38.55 ± 13.84	9.89 ± 5.89^a^	39.11 ± 9.62^b^	39.66 ± 17.67^b^
*Lactobacillus* (%)	18.50 ± 14.42	5.41 ± 5.68^a^	4.72 ± 5.58	6.75 ± 6.86
Allobaculum (%)	7.74 ± 8.90	1.56 ± 1.74	10.91 ± 8.37^b^	4.44 ± 4.35
UCG-005 (%)	0.50 ± 0.51	9.56 ± 3.96^a^	2.58 ± 3.09^b^	5.20 ± 5.90^b^
Romboutsia (%)	5.29 ± 5.79	0.84 ± 1.34	3.97 ± 4.04^b^	5.26 ± 5.80^b^
Bifidobacterium (%)	0.06 ± 0.11	0.12 ± 0.32	6.14 ± 6.32^b^	2.16 ± 4.54^b^

The data are presented as the mean ± SD, n = 8.

^a^ *p* < 0.05 vs. WKY.

^b^ *p* < 0.05 vs. untreated SHRs.

### 3.3 Results of serum and fecal metabolomics analysis

#### 3.3.1 Principal Component Analysis and system stability investigation of serum and stool samples

**FIGURE 7:**
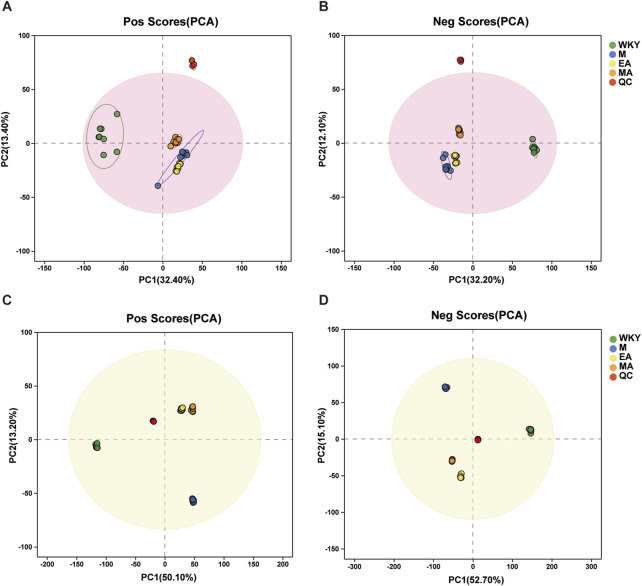
Graph of PCA Scores for Serum and Fecal Samples. **(A-B)** Serum samples; **(C-D)** Fecal samples. After the samples are analyzed by dimensionality reduction, there are relative coordinate points on the principal components p1 and p2, and the distance of each coordinate point represents the degree of aggregation and dispersion between the samples, the closer the distance indicates the higher similarity between the samples, and the further the distance indicates the higher difference between the samples. The confidence ellipse indicates that the ‘true’ samples in this group are distributed within this region at a 95% confidence level; exceeding this region indicates that the samples may be abnormal. WKY, normal control group; M, model group; EA, electroacupuncture group; MA, manual acupuncture group. QC, quality control sample.

A metabolomic analysis of serum and fecal samples from four groups of rats was conducted utilizing two modes of mass spectrometry, anionic and cationic, to identify various metabolites at distinct ionic termini. Principal Component Analysis (PCA) was employed to elucidate the most accurate differences in metabolism among the groups. Figures 7A,B present the PCA score plots for the serum samples, where the motifs associated with the WKY group are distinctly separated from those of the other groups, indicating a greater metabolic divergence. In contrast, the closer proximity of the MA and EA groups suggests that the impacts of MA and EA on the serum metabolic profiles of rats are more similar. Correspondingly, the metabolic outcomes for the fecal samples, depicted in Figures 7C,D, mirror those observed in the serum samples. The model developed for the analysis of serum and fecal metabolism was validated, with an R^2^X (cum) value exceeding 0.5, signifying a well-fitted model, as detailed in Table 2.

**TABLE 2:** Parameters of the principal component analysis model for serum and fecal samples.

A	Positive		A	Negative	
	R^2^X	R^2^X (cum)		R^2^X	R^2^X (cum)
*p*1	0.324	0.324	*p*1	0.322	0.322
*p*2	0.134	0.459	*p*2	0.121	0.443
*p*3	0.0744	0.533	*p*3	0.102	0.545

B	R^2^X	R^2^X (cum)	B	R^2^X	R^2^X (cum)
*p*1	0.501	0.501	*p*1	0.527	0.527
*p*2	0.132	0.633	*p*2	0.151	0.678
*p*3	0.102	0.735	*p*3	0.11	0.788

A is serum samples and B is fecal samples. R^2^X denotes the explanatory power of the model for differences in the X variables, and R^2^X (cum) denotes the cumulative explanatory power of the differences, with values closer to 1 indicating a better model, and lower values indicating a poorer accuracy of the model’s fit. *p*1, *p*2, and *p*3 denote the values of the first, second, and third principal component contributions, respectively.

#### 3.3.2 Serum and fecal differential metabolite analysis

**FIGURE 8:**
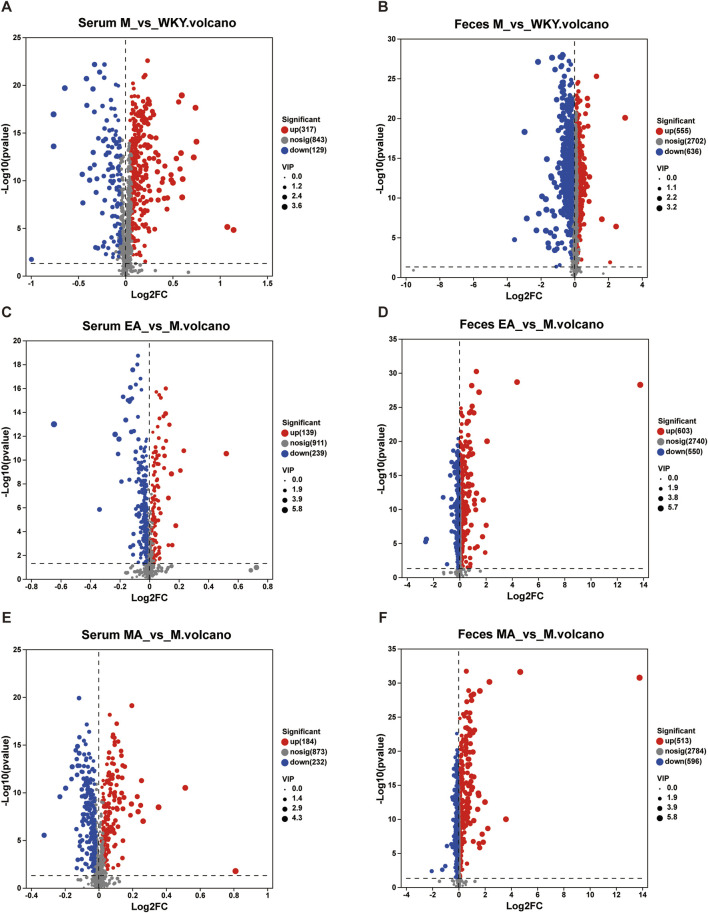
Volcano Plot of Serum and Fecal Metabolite Differences. **(A,C,E)** Serum samples; **(B,D,F)** Fecal samples. The horizontal coordinate is the value of the fold change in the difference in metabolite expression between the two groups, i.e., log_2_FC. The vertical coordinate is the value of the statistical test for differences in metabolite expression, i.e., log10 (*p*-value). The higher the *p*-value, the more significant the expression difference. The values of the horizontal and vertical coordinates are expressed as logarithms. Each dot in the graph represents a specific metabolite and the size of the dot represents the VIP value. The blue dots on the left are metabolites that are differentially downregulated in expression, and the red dots on the right are metabolites that are upregulated in expression. WKY, normal control group; M, model group; EA, electroacupuncture group; MA, manual acupuncture group.

**FIGURE 9:**
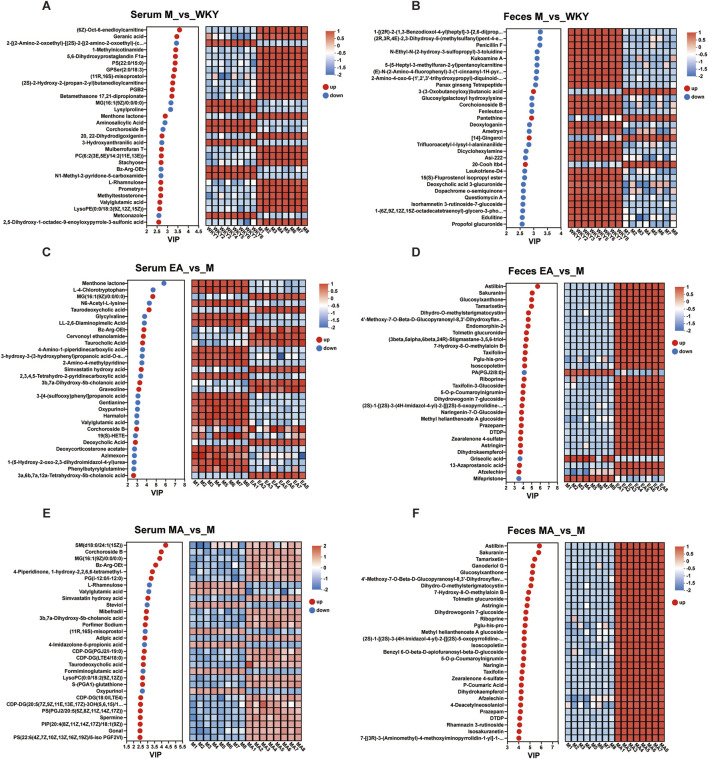
Plot of Metabolite VIP Value Analysis. **(A,C,E)** Serum samples; **(B,D,F)** Fecal samples. The metabolite VIP bubble chart is shown on the left, with the Y-axis representing the metabolites and the X-axis the VIP values, and the metabolites are arranged according to the size of the VIP values, from top to bottom. On the right side is a heat map of metabolite expression, each column represents a sample with the sample name below; each row represents a metabolite, and the color indicates the magnitude of the relative expression of that metabolite in that group of samples; the correspondence between the color gradient and the magnitude of the value is shown in the gradient color block. WKY, normal control group; M, model group; EA, electroacupuncture group; MA, manual acupuncture group.

The analysis of serum and fecal differential metabolites between groups was conducted using OPLS-DA VIP >1 and *p* < 0.05. In the M group, compared to the WKY group, 221 serum differential metabolites were identified at the cationic end and 225 at the anionic end. The top 30 metabolites, based on VIP values, were selected, including Geranic acid, PGB2, and 5,6-Dihydroxyprostaglandin F1a (Figures 8A, 9A). Similarly, fecal differential metabolites were also identified and ranked by the top 30 VIP values (Figures 8B, 9B). These findings suggest that SHRs exhibit more pronounced *in vivo* metabolite differences compared to normal rats. In the EA group, relative to the M group, 189 serum differential metabolites were identified at both the cationic and anionic termini. The top 30 metabolites, based on VIP values, were selected, including Taurodeoxycholic acid, Taurocholic Acid, and Deoxycholic Acid (Figures 8C, 9C). Similarly, fecal differential metabolites were identified and ranked by the top 30 VIP values (Figures 8D, 9D). In the MA group, compared to the M group, 226 serum differential metabolites were identified at the cationic end and 190 at the anionic end. The metabolites with the highest 30 VIP values, including Corchoroside B, Bz-Arg-OEt, and Mibefradil. (Figures 8E, 9E). Similarly, fecal differential metabolites were identified and screened based on the top 30 VIP values (Figures 8F, 9F). These findings suggest that both EA and MA influence the metabolite profiles in the serum and feces of SHRs.

#### 3.3.3 Analysis of Callback Metabolites of EA and MA

**FIGURE 10:**
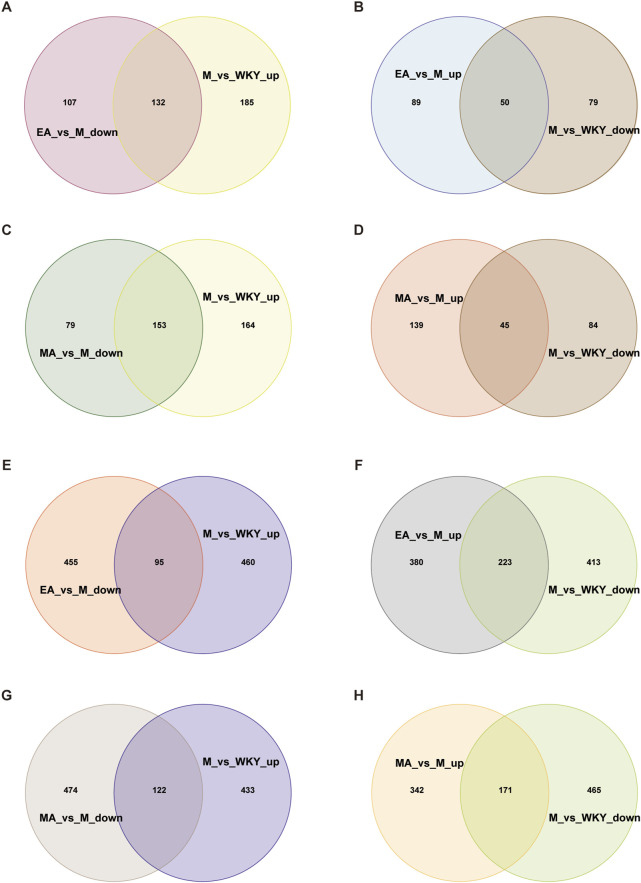
Venn Diagram of the Callback Metabolites of EA and MA. **(A,B)** Serum metabolites of EA callback. **(C,D)** Serum metabolites of MA callback. **(E,F)** Fecal metabolites of electroacupuncture callback. **(G,H)** Fecal metabolites of electroacupuncture callback. ‘Up’ for upward and ‘down’ for downward adjustments. WKY, normal control group; M, model group; EA, electroacupuncture group; MA, manual acupuncture group.

By examining the intersection of serum metabolites, we identified those that were decreased in group M compared to WKY and increased in group EA compared to group M, as well as those that were increased in group M compared to WKY and decreased in group EA compared to group M. This analysis yielded a total of 182 callback serum metabolites from the EA group (Figures 10A,B; Supplementary Table S4). Similarly, 198 callback serum metabolites were identified for the MA group (Figures 10C,D; Supplementary Table S5), of which 136 were common to both group EA and group MA, including 19(S)-HETE, PGB2, and 1-Methylnicotinamide (Supplementary Table S6). These findings indicate that manual acupuncture results in a greater number of callback serum metabolites and that there is a substantial overlap in the metabolites affected by both EA and MA.

By intersecting the fecal metabolites that were decreased in the M group compared to the WKY group and increased in the EA group compared to the M group, as well as those that were increased in the M group compared to the WKY group and decreased in the EA group compared to the M group, we identified a total of 318 callback fecal metabolites associated with EA (Figures 10E,F; Supplementary Table S7). Similarly, 293 callback fecal metabolites were identified for MA (Figures 10G,H; Supplementary Table S8). Among these, 160 fecal metabolites, such as 6-Keto-prostaglandin F1a, Indole-3-Carboxaldehyde, 7-α,27-Dihydroxycholesterol, 5-Hydroxy-N-formylkynurenine, were common to both EA and MA (Supplementary Table S9). These findings indicate that EA results in a greater number of callback fecal metabolites and that both EA and MA share a subset of these metabolites.

#### 3.3.4 Metabolic pathways involved in EA and MA

**FIGURE 11:**
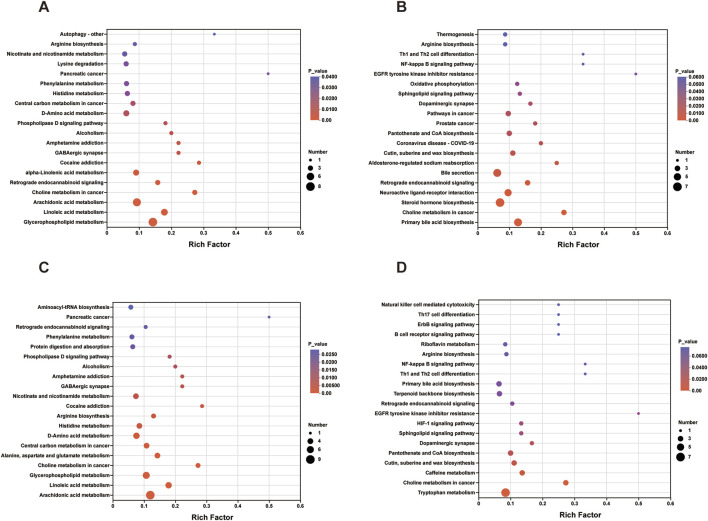
Enrichment Analysis of KEGG Pathway for Metabolites Regulated by EA and MA. **(A)** KEGG pathway enrichment analysis of serum metabolites regulated by EA. **(B)** KEGG pathway enrichment analysis of fecal metabolites regulated by EA. **(C)** KEGG pathway enrichment analysis of serum metabolites regulated by MA. **(D)** KEGG pathway enrichment analysis of fecal metabolites regulated by MA. The horizontal coordinate is the enrichment rate; the vertical coordinate is the KEGG pathway. The size of the bubbles in the graph represents how much of the pathway is enriched to the metabolic set of compounds, and the color of the bubbles indicates the magnitude of the *p*-value for different enrichment significance.

The serum and fecal metabolites from the EA callback were independently subjected to KEGG pathway enrichment analysis. The metabolic pathways implicated in the callback of serum metabolites associated with hypertension included glycerophospholipid metabolism, linoleic acid metabolism, arachidonic acid metabolism, along with 11 additional pathways (Figure 11A; Table 3). In contrast, the primary metabolic pathways involved in the callback of fecal metabolites pertinent to hypertension encompassed steroid hormone biosynthesis, aldosterone-regulated sodium reabsorption, and dopaminergic synapses, among 7 other pathways (Figure 11B; Table 3).

**TABLE 3:** Listing of selected KEGG-enriched pathways for metabolite KEGG callback by EA and MA associated with hypertension.

Pathway description	Pathway ID	Num	*p* value	Samples	Treatment
Glycerophospholipid metabolism	map00564	12	4.12E-07	serum	electroacupuncture
Linoleic acid metabolism	map00591	5	2.36E-05	serum	electroacupuncture
Arachidonic acid metabolism	map00590	7	4.14E-05	serum	electroacupuncture
alpha-Linolenic acid metabolism	map00592	4	0.002311	serum	electroacupuncture
GABAergic synapse	map04727	2	0.005563	serum	electroacupuncture
Alcoholism	map05034	2	0.006896	serum	electroacupuncture
Phospholipase D signaling pathway	map04072	2	0.008359	serum	electroacupuncture
Arginine biosynthesis	map00220	2	0.03483	serum	electroacupuncture
Autophagy - other	map04136	2	0.03824	serum	electroacupuncture
cAMP signaling pathway	map04024	2	0.04063	serum	electroacupuncture
Alanine, aspartate and glutamate metabolism	map00250	2	0.04996	serum	electroacupuncture
Primary bile acid biosynthesis	map00120	6	0.0001279	fece	electroacupuncture
Steroid hormone biosynthesis	map00140	6	0.001388	fece	electroacupuncture
Neuroactive ligand-receptor interaction	map04080	5	0.001797	fece	electroacupuncture
Retrograde endocannabinoid signaling	map04723	6	0.003849	fece	electroacupuncture
Aldosterone-regulated sodium reabsorption	map04960	2	0.007593	fece	electroacupuncture
Dopaminergic synapse	map04728	5	0.01712	fece	electroacupuncture
Sphingolipid signaling pathway	map04071	5	0.02635	fece	electroacupuncture
Arachidonic acid metabolism	map00590	9	2.49E-07	serum	manual acupuncture
Linoleic acid metabolism	map00591	5	1.98E-05	serum	manual acupuncture
Glycerophospholipid metabolism	map00564	10	5.73E-05	serum	manual acupuncture
Alanine, aspartate and glutamate metabolism	map00250	5	0.0003549	serum	manual acupuncture
Arginine biosynthesis	map00220	4	0.002722	serum	manual acupuncture
GABAergic synapse	map04727	3	0.005194	serum	manual acupuncture
Alcoholism	map05034	3	0.00644	serum	manual acupuncture
Phospholipase D signaling pathway	map04072	3	0.007809	serum	manual acupuncture
Neuroactive ligand-receptor interaction	map04080	4	0.02635	serum	manual acupuncture
cAMP signaling pathway	map04024	2	0.03811	serum	manual acupuncture
Tryptophan metabolism	map00380	7	0.0004447	fece	manual acupuncture
Caffeine metabolism	map00232	3	0.005684	fece	manual acupuncture
Dopaminergic synapse	map04728	5	0.0167	fece	manual acupuncture
Sphingolipid signaling pathway	map04071	5	0.02572	fece	manual acupuncture
Primary bile acid biosynthesis	map00120	6	0.04433	fece	manual acupuncture
Th1 and Th2 cell differentiation	map04658	4	0.0499	fece	manual acupuncture
NF-kappa B signaling pathway	map04064	4	0.0499	fece	manual acupuncture

Table colour meanings: blue indicates serum metabolites in the EA, group; red indicates fecal metabolites in the EA, group; green indicates serum metabolites in the MA, group; purple indicates fecal metabolites in the MA, group. Pathway ID, the number given for each KEGG, pathway in the KEGG, database. Num, the number of metabolites annotated to the pathway by the metabolic set; metabolites. The *p* value for different enrichment significance.

Similarly, serum and fecal metabolites from the MA callback were analyzed separately for KEGG pathway enrichment. The metabolic pathways involved in the callback of serum metabolites related to hypertension included linoleic acid metabolism, arachidonic acid metabolism, arginine biosynthesis, and 10 additional pathways (Figure 11C; Table 3). The principal metabolic pathways involved in the callback of fecal metabolites associated with hypertension comprised tryptophan metabolism, primary bile acid biosynthesis, dopaminergic synapses, and 7 other pathways (Figure 11D; Table 3).

## 4 Discussion

In a prior investigation conducted by our research team (Liu et al., 2023), it was demonstrated that acupuncture applied to the Taichong and Zusanli in SHRs enhanced both the functional activities and the functional connectivity between SHRs and brain regions associated with blood pressure regulation, thereby effectively reducing systolic blood pressure in these rats. The present study corroborates these findings by confirming that acupuncture significantly decreases both systolic and diastolic blood pressure in SHRs. Moreover, it was observed that EA induces a more rapid antihypertensive effect compared to MA when contrasted with the model group. Additionally, the study revealed that the systolic blood pressure of rats was more sensitive to acupuncture than the diastolic blood pressure. It has been suggested that the mechanism of action for acupuncture involves multiple pathways (Lai et al., 2012). Consequently, building upon our group’s previous findings regarding central mechanisms, the present study extends the investigation to explore the peripheral mechanisms underlying acupuncture-induced hypotension, employing microbial diversity analysis and untargeted metabolomics techniques.

The relationship between intestinal microecological disturbances and hypertension has been evidenced in both hypertensive patients and animal models (Li et al., 2017; Lv et al., 2023; Wang T. et al., 2021; Yan D et al., 2022). Dysbiosis of gut microbiota contributes to elevated blood pressure through mechanisms involving SCFAs, TMAO (Yang et al., 2023), hormonal regulation including gaseous signaling molecules, gut bacteria-derived bioactive peptides, serotonin, steroid hormones, and immune responses (Ferguson et al., 2019; Iwaniak et al., 2014; Kang and Cai, 2018). The findings of the current study indicate that the microbial composition in SHRs became more similar to that of normotensive WKY rats following EA and MA interventions, suggesting that acupuncture may ameliorate intestinal flora imbalances in SHRs. Notably, this study observed a greater diversity of gut microbiota in SHRs compared to WKY rats. EA and MA interventions were observed to decrease flora diversity, leading us to hypothesize that needling may reduce the abundance of harmful bacteria. Some studies have found that EA treatment can reduce the abundance of intestinal flora in hypertensive patients compared with the hypertensive control group (Wang J et al., 2021), which is consistent with our results. Furthermore, acupuncture was shown to increase the presence of beneficial bacteria, such as Blautia, Bifidobacterium, and the Ruminococcus_gauvreauii_group, which were reduced in abundance in group M, while harmful bacteria like *Helicobacter* were more prevalent in group M. Additionally, acupuncture inhibited the overgrowth of conditionally pathogenic bacteria such as UCG-005. LEfSe analyses further revealed that both EA and MA enhanced the influence of Blautia and Bifidobacterium, thereby modulating the gut flora structure in SHRs. Notably, Blautia was significantly elevated in both the EA and MA groups, which is associated with the promotion of SCFAs production, particularly increasing levels of acetate, butyrate, and propionate (Huang et al., 2023). Emerging evidence suggests that SCFAs are integral to the regulation of hypertension (Hu et al., 2022). An increase in Bifidobacterium levels has been linked to the secretion of anti-inflammatory cytokines, which mitigate the chronic inflammation associated with hypertension (Michels et al., 2022). The diminution of UCG-005 may contribute to blood pressure reduction by enhancing vascular endothelial function through the decreased production of metabolites such as TMAO (Min et al., 2023). Furthermore, LEfSe analysis identified Romboutsia as the signature bacterium in the EA group, which is correlated with butyrate production (Jiang et al., 2025). Conversely, the MA group was characterized by the presence of Allobaculum, associated with tryptophan metabolism (Chen et al., 2024). These findings suggest that the two acupuncture modalities influence blood pressure through distinct microbial pathways. Specifically, EA may exert its effects by ameliorating insulin resistance via increased butyrate production (Chen Z. et al., 2021; Rosell-Díaz et al., 2024), while MA may regulate blood pressure by modulating neurotransmitter levels, such as 5-hydroxytryptamine, through alterations in tryptophan metabolic pathways (Ciuclan et al., 2013; Watts, 2005). The findings of this study indicate that the antihypertensive effects of EA and MA may be associated with the amelioration of gut microbial dysbiosis.

Serum metabolomics analysis revealed that 182 serum metabolites in the EA group and 198 in the MA group were enriched across nine metabolic pathways, including those related to glycerophospholipids, linoleic acid, and arachidonic acid metabolism. Glycerophospholipids, which are essential components of cell membranes, play a crucial role in lipid transport and metabolism. Dysregulation in glycerophospholipid metabolism can result in dyslipidemia, characterized by elevated plasma triglycerides and cholesterol levels. This condition can lead to increased blood viscosity, atherosclerosis, reduced vascular elasticity, heightened peripheral resistance, and elevated blood pressure (Chen H et al., 2021; Tian et al., 2020). In this study, we observed that both EA and MA effectively reduced the elevated levels of phosphatidylcholine (PC) in SHRs when compared to WKY rats. PC is integral to the metabolic processes associated with hypertension, primarily due to its specific catalysis by various phospholipase A (PLA) enzymes during catabolism, leading to the production of lysophosphatidylcholine. These metabolites are implicated in inflammation and lipid signaling, and the elevation of inflammatory lipids, such as lysophosphatidylcholine and long-chain fatty acids, can induce endothelial dysfunction, potentially predisposing individuals to hypertensive conditions (Huynh et al., 2002). Furthermore, our findings indicate that acupuncture can increase linoleic acid levels in SHRs, which is significant as linoleic acid has been shown to reduce vascular reactivity, thereby enhancing vascular function in patients with hypertension (Nunes et al., 2018). Linoleic acid is metabolized through a series of desaturase and elongase enzymes to yield arachidonic acid, an essential fatty acid (Fox et al., 2000). Metabolites of arachidonic acid are crucial in the regulation of vascular tone and hypertension (Zhou Y et al., 2021). Our findings suggest that EA may influence blood pressure regulation via the linolenic acid metabolic pathway. α-Linolenic acid, another essential fatty acid, exhibits significant physiological activities (Folino et al., 2015). It has been shown to reduce inflammatory markers such as TNF-α, IL-6, and hs-CRP, thereby enhancing endothelial cell-mediated vasodilation (Li et al., 2020).

The fecal metabolomics analysis revealed that 318 fecal metabolites modulated by EA and 293 metabolites influenced by MA were collectively implicated in three metabolic pathways: primary bile acid biosynthesis, dopaminergic synapses, and sphingolipid signaling. A prior investigation conducted by our research group demonstrated that EA could exert antihypertensive effects by modulating primary bile acid biosynthesis, tryptophan metabolism, and other metabolic pathways in SHRs (Yang et al., 2022a). The metabolic pathway of primary bile acid biosynthesis plays a crucial role in maintaining blood pressure stability through various mechanisms (Sánchez, 2018; Shulpekova et al., 2022; Xia et al., 2021). Primary bile acids contribute to the improvement of intestinal microecological balance by promoting the proliferation of beneficial bacteria and inhibiting the colonization of pathogenic bacteria (Qi et al., 2022; Ramírez-Pérez et al., 2017). Bile acids function as signaling molecules that activate various receptors, such as the farnesol X receptor (FXR), pregnane X receptor (PXR), and vitamin D receptor (VDR), thereby influencing blood pressure through multiple pathways (Porez et al., 2012). For instance, bile acids can enhance hepatic and intestinal circulation by modulating FXR to mitigate inflammatory responses (Guizoni et al., 2020; Porez et al., 2012; Zhang et al., 2008). Additionally, research has demonstrated that the microbiota may influence steroid hormone metabolism in individuals with hypertension (Mishima and Abe, 2022). In the current study, EA was observed to have a more pronounced effect on modulating the fecal steroid hormone biosynthesis pathway compared to MA. Furthermore, MA impacts the crucial pathway of tryptophan metabolism in feces, with the kynurenine metabolic pathway serving as the primary route for tryptophan catabolism in most mammalian cells (Song et al., 2017). Kynurenine and its metabolites, including 3-hydroxykynurenine, possess neuroactive properties and are capable of traversing the blood-brain barrier, thereby influencing neurotransmitter homeostasis and modulating sympathetic nervous system activity (Hsu and Tain, 2020; Kennedy et al., 2017). Enhanced sympathetic activity subsequently elevates heart rate, induces vasoconstriction, and increases peripheral resistance, cumulatively contributing to elevated blood pressure. Additionally, certain intermediates within the kynurenine pathway exhibit immunomodulatory properties, with dysregulated immune responses potentially inciting vascular inflammation, damaging endothelial cells, impairing vascular function, and playing a role in the pathogenesis of hypertension (Irsik et al., 2024). Furthermore, in the presence of intestinal microbiota, tryptophan is converted into indole and its derivatives, such as indole-3-propionic acid. These indoles have the capacity to activate certain receptors within the gastrointestinal tract, including the aromatic hydrocarbon receptor (AhR), thereby modulating intestinal immunity and barrier integrity (Nicolas and Chang, 2019; Sun et al., 2023). Compromise of the intestinal barrier function facilitates the translocation of endotoxins and other deleterious substances into the bloodstream, which can initiate an inflammatory response. This inflammatory cascade may result in vascular endothelial dysfunction, subsequently impairing the vasodilatory and vasoconstrictive functions of blood vessels, and consequently influencing blood pressure regulation (Paeslack et al., 2022).

This study has several limitations in the following areas. First of all, SHRs were employed as an experimental model. Although SHRs offer slightly less clinical relevance than human hypertensive patients, compared with hypertensive patients, SHRs generally have a single disease, hypertensive patients often have other diseases. The intestinal flora and metabolites of SHRs are less affected by other diseases. We will study the effects of acupuncture on the gut microbiota and metabolites in other animal models, and then proceed to clinical trials. Secondly, We did not extract specific values from fecal microbiota and metabolites for correlation analysis. In our discussion, we elaborated on the relationship between key microbiota and metabolites based on previous literature studies. In future studies targeting metabolites, we will extract values from intestinal products and microbiota for correlation analysis.

## 5 Conclusion

In this study, we observed that both EA and MA exhibit antihypertensive effects through the modulation of gut microbiota composition and alterations in metabolite profiles in SHRs. Acupuncture was found to enhance the abundance of beneficial bacteria, such as Blautia, which are typically diminished in SHRs, while reducing the abundance of harmful bacteria like *Helicobacter*, thereby contributing to the normalization of the gut flora structure. Furthermore, both EA and MA were shown to influence serum glycerophospholipids, linoleic acid, arachidonic acid, and other metabolic pathways in SHRs, thereby facilitating blood pressure regulation. In terms of fecal metabolic pathways, these acupuncture modalities primarily modulate primary bile acid biosynthesis and tryptophan pathways. Notably, it was also identified that EA and MA do not target the same microbial taxa and metabolites, which may account for the observed differences in their therapeutic efficacy. Untargeted metabolomics is a hypothesis-generating approach, consider presenting it as a first step of a set of studies. In future studies, we will further apply targeted metabolomics techniques in clinical studies to investigate the changes in metabolites, so as to identify the key targets for blood pressure regulation by acupuncture.

## Data Availability

The data presented in the study are deposited in the BioProject database, accession number PRJNA1284070.
