# Supplementary material for: Differential effects of electroacupuncture and manual acupuncture on spontaneously hypertensive rats: insights from intestinal microbiota and metabolomics
Source: Front Mol Biosci. 2025 Jul 8;12:1619356. doi: 10.3389/fmolb.2025.1619356 (PMC12279516; doi:10.3389/fmolb.2025.1619356)
Supplement: Supplementary file 1 [file Supplementaryfile1.docx]

Supplementary Material

Differential Effects of Electroacupuncture and Manual Acupuncture on Hypertension: Insights from Intestinal Microbiota and Metabolomics

**Ji-peng Liu^1^**^✝^**, Long-teng Tu^1^**^✝^**, Ke-zhen Yang^2^, Yin-yin Li^1,3^, Yu Gong^1^, Bing-xuan Han^1^, Yu Liu^1^, Xiao-min Hao^1^, Bing-nan Yue^1^, Jing Zhang^1^, Gui-rong Luo^1^, Meng Xu^4*^, Qing-guo Liu^1*^**

^✝^These authors have contributed equally to this work.

**Co-first author:**Ji-peng Liu
1033376969@qq.com
Long-teng Tu
tulongteng888@163.com

*** Correspondence:**Qing-guo Liu
liuqingguo888@vip.sina.com
Meng Xu
chilli.xu@163.com

**Supplementary Table 1. Statistical table for the test of difference in intestinal flora between group MA and group M**

| Species name | MA-Mean  (%) | MA-Sd  (%) | | M-Mean  (%) | M-Sd  (%) | *p* value |
| --- | --- | --- | --- | --- | --- | --- |
| g__Alistipes | 0.000234 | 0.0006618 | 0.0124 | | 0.02574 | 0.03944 |
| g__Coriobacteriaceae_UCG-002 | 0.03159 | 0.02853 | 0.008423 | | 0.01866 | 0.03852 |
| g__Pygmaiobacter | 0.0004679 | 0.001324 | 0.01334 | | 0.01545 | 0.03452 |
| g__Anaerofilum | 0 | 0 | 0.004679 | | 0.007878 | 0.03247 |
| g__Ruminiclostridium | 0 | 0 | 0.004679 | | 0.007074 | 0.03247 |
| g__Candidatus_Stoquefichus | 0.02035 | 0.02938 | 0.00117 | | 0.001985 | 0.03239 |
| g__Romboutsia | 3.974 | 4.039 | 0.8371 | | 1.34 | 0.03132 |
| g__CAG-56 | 0.003275 | 0.005174 | 0.03486 | | 0.06224 | 0.02862 |
| g__Globicatella | 0.001872 | 0.002451 | 0.01029 | | 0.01119 | 0.02754 |
| g__Monoglobus | 0.4101 | 0.2328 | 1.376 | | 1.856 | 0.02731 |
| g__Ruminococcus | 0.3643 | 0.3985 | 1.127 | | 0.8764 | 0.02395 |
| g__Candidatus_Soleaferrea | 0.0009359 | 0.001 | 0.008423 | | 0.006406 | 0.01071 |
| g__Collinsella | 0.2012 | 0.3357 | 0.001638 | | 0.002108 | 0.0104 |
| g__Lachnoclostridium | 0.4523 | 0.766 | 2.034 | | 1.171 | 0.01008 |
| g__Eubacterium_brachy_group | 0.1083 | 0.106 | 0.2634 | | 0.09477 | 0.01008 |
| g__Eubacterium_oxidoreducens_group | 0.0358 | 0.04585 | 0.1097 | | 0.04396 | 0.01008 |
| g__Bacteroides_pectinophilus_group | 0.000234 | 0.0006618 | 0.1787 | | 0.1667 | 0.009218 |
| g__UCG-009 | 0.001404 | 0.00218 | 0.01521 | | 0.01448 | 0.007469 |
| g__Streptococcus | 0.09125 | 0.05296 | 0.2415 | | 0.113 | 0.007406 |
| g__Anaerostipes | 1.834 | 1.77 | 0.1596 | | 0.3813 | 0.007232 |
| g__Lachnospiraceae_UCG-006 | 0.1525 | 0.1943 | 1.958 | | 1.696 | 0.005385 |
| g__Coprococcus | 0.03322 | 0.04495 | 0.1771 | | 0.1341 | 0.005385 |
| g__Anaerovorax | 0.001404 | 0.002785 | 0.02948 | | 0.02316 | 0.00518 |
| g__Defluviitaleaceae_UCG-011 | 0.003275 | 0.004772 | 0.02129 | | 0.0168 | 0.004698 |
| g__UCG-005 | 2.579 | 3.091 | 9.56 | | 3.958 | 0.003876 |
| g__Ruminococcus_gauvreauii_group | 4.004 | 1.655 | 0.8834 | | 0.5433 | 0.003876 |
| g__Colidextribacter | 0.07159 | 0.08767 | 0.2887 | | 0.1131 | 0.003876 |
| g__Eubacterium_siraeum_group | 0.0241 | 0.02421 | 0.2031 | | 0.2289 | 0.003824 |
| g__UCG-003 | 0.002106 | 0.005956 | 0.01217 | | 0.009332 | 0.003642 |
| g__Papillibacter | 0.000234 | 0.0006618 | 0.01123 | | 0.01201 | 0.002904 |
| g__Intestinimonas | 0.00234 | 0.005898 | 0.02386 | | 0.02115 | 0.002494 |
| g__Helicobacter | 0.000234 | 0.0006618 | 0.01287 | | 0.01013 | 0.002455 |
| g__Oscillibacter | 0.003275 | 0.005077 | 0.02457 | | 0.01413 | 0.002056 |
| g__Allobaculum | 10.91 | 8.365 | 1.559 | | 1.736 | 0.001948 |
| g__Christensenellaceae_R-7_group | 0.2375 | 0.1367 | 0.9735 | | 0.4304 | 0.001948 |
| g__Bifidobacterium | 6.135 | 6.323 | 0.1179 | | 0.3177 | 0.001933 |
| g__NK4A214_group | 0.06691 | 0.1139 | 0.9211 | | 0.8439 | 0.001933 |
| g__Eubacterium_nodatum_group | 0.01123 | 0.0168 | 0.08516 | | 0.05619 | 0.001888 |
| g__Dubosiella | 0.4139 | 0.4108 | 0.0241 | | 0.04579 | 0.001787 |
| g__Prevotellaceae_UCG-003 | 0.01614 | 0.01864 | 0 | | 0 | 0.001446 |
| g__Eubacterium_xylanophilum_group | 0.314 | 0.3054 | 2.964 | | 2.147 | 0.001359 |
| g__Acetitomaculum | 0.1184 | 0.1205 | 0.7311 | | 0.6871 | 0.001359 |
| g__A2 | 0.00234 | 0.00527 | 0.2176 | | 0.2829 | 0.001206 |
| g__Blautia | 39.11 | 9.62 | 9.887 | | 5.888 | 0.0009391 |
| g__Lachnospiraceae_NK4A136_group | 0.1137 | 0.1246 | 3.37 | | 3.282 | 0.0009391 |
| g__Roseburia | 0.05264 | 0.06161 | 3.5 | | 4.05 | 0.000931 |
| g__UBA1819 | 0.003275 | 0.00371 | 0.1032 | | 0.1004 | 0.0008295 |
| g__UCG-007 | 0.000234 | 0.0006618 | 0.01872 | | 0.01368 | 0.0007949 |
| g__GCA-900066575 | 0.003042 | 0.005095 | 0.1453 | | 0.1398 | 0.000771 |
| g__Family_XIII_UCG-001 | 0.0004679 | 0.001324 | 0.04258 | | 0.0473 | 0.0005535 |

Notes: Species name is the name of the species; Mean (%) is the mean relative abundance of the species in different subgroups; Sd (%) is the standard deviation; *p* value is the probability of false positives, which is a commonly used decision value in statistics. M, model group; MA, manual acupuncture group.

**Supplementary Table 2. Statistical table for the test of difference in intestinal flora between group EA and group M**

| Species name | M-Mean  (%) | M-Sd  (%) | EA-Mean  (%) | EA-Sd  (%) | *p* value |
| --- | --- | --- | --- | --- | --- |
| g__Dubosiella | 0.0241 | 0.04579 | 0.2129 | 0.3903 | 0.04843 |
| g__CAG-56 | 0.03486 | 0.06224 | 0.003977 | 0.004632 | 0.04409 |
| g__Ruminococcus | 1.127 | 0.8764 | 0.4459 | 0.4493 | 0.04057 |
| g__Anaerofilum | 0.004679 | 0.007878 | 0 | 0 | 0.03247 |
| g__Clostridium_sensu_stricto_1 | 0.02667 | 0.03582 | 0.1015 | 0.0734 | 0.03058 |
| g__Defluviitaleaceae_UCG-011 | 0.02129 | 0.0168 | 0.006785 | 0.004895 | 0.02862 |
| g__Oscillibacter | 0.02457 | 0.01413 | 0.009125 | 0.01574 | 0.02607 |
| g__Eubacterium_xylanophilum_group | 2.964 | 2.147 | 1.098 | 0.9142 | 0.02395 |
| g__Helicobacter | 0.01287 | 0.01013 | 0.002574 | 0.002635 | 0.02191 |
| g__Bacteroides_pectinophilus_group | 0.1787 | 0.1667 | 0.004913 | 0.01179 | 0.02147 |
| g__Bifidobacterium | 0.1179 | 0.3177 | 2.158 | 4.537 | 0.02067 |
| g__Butyricicoccus | 0.0117 | 0.0117 | 0.0007019 | 0.001393 | 0.01829 |
| g__Globicatella | 0.01029 | 0.01119 | 0.001638 | 0.003931 | 0.01823 |
| g__UCG-005 | 9.56 | 3.958 | 5.189 | 5.898 | 0.01813 |
| g__Erysipelotrichaceae_UCG-003 | 0.1102 | 0.09455 | 0.3355 | 0.205 | 0.01813 |
| g__Colidextribacter | 0.2887 | 0.1131 | 0.09639 | 0.1574 | 0.01813 |
| g__Candidatus_Soleaferrea | 0.008423 | 0.006406 | 0.001404 | 0.002599 | 0.01417 |
| g__Romboutsia | 0.8371 | 1.34 | 5.258 | 5.796 | 0.01359 |
| g__Fusicatenibacter | 0.009593 | 0.0116 | 0.9674 | 2.156 | 0.01331 |
| g__Jeotgalicoccus | 0.011 | 0.01489 | 0 | 0 | 0.01278 |
| g__Parvibacter | 0.01029 | 0.00557 | 0.002808 | 0.004585 | 0.01075 |
| g__UCG-003 | 0.01217 | 0.009332 | 0.002106 | 0.003528 | 0.008699 |
| g__GCA-900066575 | 0.1453 | 0.1398 | 0.02386 | 0.05931 | 0.006935 |
| g__UCG-009 | 0.01521 | 0.01448 | 0.001638 | 0.003384 | 0.006934 |
| g__Collinsella | 0.001638 | 0.002108 | 0.09569 | 0.1071 | 0.006521 |
| g__UCG-007 | 0.01872 | 0.01368 | 0.00234 | 0.004332 | 0.005887 |
| g__A2 | 0.2176 | 0.2829 | 0.02035 | 0.05085 | 0.005051 |
| g__Rothia | 0.1034 | 0.07719 | 0.0124 | 0.02021 | 0.003569 |
| g__Anaerovorax | 0.02948 | 0.02316 | 0.0007019 | 0.001985 | 0.002928 |
| g__Lachnospiraceae_NK4A136_group | 3.37 | 3.282 | 0.4387 | 0.4802 | 0.002762 |
| g__Roseburia | 3.5 | 4.05 | 0.2302 | 0.4789 | 0.002762 |
| g__Lachnoclostridium | 2.034 | 1.171 | 0.4789 | 0.3908 | 0.001948 |
| g__Intestinimonas | 0.02386 | 0.02115 | 0.00117 | 0.002223 | 0.001455 |
| g__Prevotellaceae_UCG-003 | 0 | 0 | 0.09148 | 0.1259 | 0.001446 |
| g__Blautia | 9.887 | 5.888 | 39.66 | 17.67 | 0.001359 |
| g__Christensenellaceae_R-7_group | 0.9735 | 0.4304 | 0.2131 | 0.09865 | 0.001359 |
| g__UBA1819 | 0.1032 | 0.1004 | 0.005381 | 0.009619 | 0.001251 |
| g__Eubacterium_nodatum_group | 0.08516 | 0.05619 | 0.005615 | 0.005294 | 0.0009148 |
| g__Family_XIII_UCG-001 | 0.04258 | 0.0473 | 0.0009359 | 0.001733 | 0.0006754 |

Notes: Species name is the name of the species; Mean (%) is the mean relative abundance of the species in different subgroups; Sd (%) is the standard deviation; *p* value is the probability of false positives, which is a commonly used decision value in statistics. M, model group; EA, electroacupuncture group.

**Supplementary Table 3. Statistical table for the test of difference in intestinal flora between group WKY and group M**

| Species name | M-Mean  (%) | M-Sd  (%) | WKY-Mean  (%) | WKY-Sd  (%) | *p* value |
| --- | --- | --- | --- | --- | --- |
| g__Dubosiella | 0.0241 | 0.04579 | 0.1027 | 0.1184 | 0.04997 |
| g__Acinetobacter | 0.000234 | 0.0006618 | 0.001638 | 0.001562 | 0.04414 |
| g__Lachnospiraceae_ND3007_group | 0.08914 | 0.1322 | 0.01497 | 0.02954 | 0.03983 |
| g__Collinsella | 0.001638 | 0.002108 | 5.996 | 6.73 | 0.03535 |
| g__Parvibacter | 0.01029 | 0.00557 | 0.004211 | 0.003971 | 0.03461 |
| g__Lachnospira | 0.01193 | 0.02043 | 0 | 0 | 0.03247 |
| g__Acetatifactor | 0.01029 | 0.02264 | 0 | 0 | 0.03247 |
| g__Ruminiclostridium | 0.004679 | 0.007074 | 0 | 0 | 0.03247 |
| g__Lachnospiraceae_NK4B4_group | 0.01076 | 0.02309 | 0 | 0 | 0.03225 |
| g__Akkermansia | 0.02691 | 0.06126 | 0.0009359 | 0.002647 | 0.03197 |
| g__Lactobacillus | 5.411 | 5.678 | 18.5 | 14.42 | 0.02395 |
| g__Ruminococcus_torques_group | 0.1993 | 0.2347 | 0.8144 | 0.641 | 0.02067 |
| g__DNF00809 | 0.04211 | 0.03161 | 0.01334 | 0.0174 | 0.0203 |
| g__CAG-56 | 0.03486 | 0.06224 | 0.004445 | 0.01257 | 0.01849 |
| g__Erysipelotrichaceae_UCG-003 | 0.1102 | 0.09455 | 0.6832 | 0.7388 | 0.01813 |
| g__Lachnospiraceae_FCS020_group | 0.01942 | 0.01357 | 0.003042 | 0.006479 | 0.01299 |
| g__Acetitomaculum | 0.7311 | 0.6871 | 0.5112 | 1.367 | 0.01291 |
| g__Dorea | 0.05381 | 0.04716 | 0.008657 | 0.01861 | 0.01284 |
| g__Pygmaiobacter | 0.01334 | 0.01545 | 0 | 0 | 0.01278 |
| g__Prevotellaceae_NK3B31_group | 0.007019 | 0.01187 | 0 | 0 | 0.01268 |
| g__Eubacterium | 0 | 0 | 0.009125 | 0.02133 | 0.01258 |
| g__Quinella | 0 | 0 | 0.004913 | 0.005828 | 0.01258 |
| g__Alloprevotella | 0.000234 | 0.0006618 | 0.004211 | 0.004446 | 0.01252 |
| g__Bacteroides | 0.01193 | 0.01044 | 0.004679 | 0.0118 | 0.01102 |
| g__CHKCI002 | 0.05171 | 0.06232 | 0.219 | 0.132 | 0.009862 |
| g__Bacteroides_pectinophilus_group | 0.1787 | 0.1667 | 0.000234 | 0.0006618 | 0.009218 |
| g__Prevotella | 0.000234 | 0.0006618 | 0.02644 | 0.03776 | 0.009218 |
| g__Helicobacter | 0.01287 | 0.01013 | 0.00117 | 0.001985 | 0.008145 |
| g__Eubacterium_ventriosum_group | 0.2248 | 0.3131 | 0.02176 | 0.03095 | 0.007232 |
| g__Lachnoclostridium | 2.034 | 1.171 | 0.6942 | 0.5023 | 0.005385 |
| g__Corynebacterium | 0.08586 | 0.05154 | 0.02503 | 0.01533 | 0.005317 |
| g__Gordonibacter | 0.01497 | 0.0188 | 0 | 0 | 0.00453 |
| g__Globicatella | 0.01029 | 0.01119 | 0.0004679 | 0.001324 | 0.004197 |
| g__Subdoligranulum | 0.0009359 | 0.002001 | 0.7948 | 1.035 | 0.00367 |
| g__Defluviitaleaceae_UCG-011 | 0.02129 | 0.0168 | 0.001872 | 0.005294 | 0.003561 |
| g__Anaerovorax | 0.02948 | 0.02316 | 0.0007019 | 0.001985 | 0.002928 |
| g__Papillibacter | 0.01123 | 0.01201 | 0.000234 | 0.0006618 | 0.002904 |
| g__Candidatus_Soleaferrea | 0.008423 | 0.006406 | 0.000234 | 0.0006618 | 0.002833 |
| g__Roseburia | 3.5 | 4.05 | 0.2176 | 0.4924 | 0.002762 |
| g__Candidatus_Saccharimonas | 3.184 | 1.491 | 0.5606 | 0.8451 | 0.002762 |
| g__Streptococcus | 0.2415 | 0.113 | 0.06972 | 0.04653 | 0.002762 |
| g__UCG-003 | 0.01217 | 0.009332 | 0.00117 | 0.003309 | 0.002108 |
| g__Anaerostipes | 0.1596 | 0.3813 | 5.023 | 6.616 | 0.001888 |
| g__Rothia | 0.1034 | 0.07719 | 0.008189 | 0.00985 | 0.001565 |
| g__Prevotellaceae_UCG-003 | 0 | 0 | 0.04188 | 0.03963 | 0.001446 |
| g__Colidextribacter | 0.2887 | 0.1131 | 0.02948 | 0.05709 | 0.001359 |
| g__Coprococcus | 0.1771 | 0.1341 | 0.01544 | 0.02105 | 0.001337 |
| g__Fusicatenibacter | 0.009593 | 0.0116 | 1.205 | 2.315 | 0.001326 |
| g__Intestinimonas | 0.02386 | 0.02115 | 0.0009359 | 0.002647 | 0.0009868 |
| g__Blautia | 9.887 | 5.888 | 38.55 | 13.84 | 0.0009391 |
| g__Marvinbryantia | 2.338 | 1.23 | 0.4029 | 0.2642 | 0.0009391 |
| g__Monoglobus | 1.376 | 1.856 | 0.06457 | 0.08309 | 0.0009391 |
| g__Ruminococcus | 1.127 | 0.8764 | 0.08048 | 0.08571 | 0.0009391 |
| g__Christensenellaceae_R-7_group | 0.9735 | 0.4304 | 0.09312 | 0.05972 | 0.0009391 |
| g__Eubacterium_brachy_group | 0.2634 | 0.09477 | 0.04445 | 0.05461 | 0.0009391 |
| g__UCG-005 | 9.56 | 3.958 | 0.5023 | 0.5094 | 0.000931 |
| g__Enterorhabdus | 0.3764 | 0.2991 | 0.02223 | 0.01359 | 0.000931 |
| g__Eubacterium_xylanophilum_group | 2.964 | 2.147 | 0.09499 | 0.2004 | 0.0009069 |
| g__NK4A214_group | 0.9211 | 0.8439 | 0.01731 | 0.03723 | 0.0009069 |
| g__Lachnospiraceae_NK4A136_group | 3.37 | 3.282 | 0.06738 | 0.1704 | 0.0008989 |
| g__Eubacterium_siraeum_group | 0.2031 | 0.2289 | 0.003042 | 0.003867 | 0.0008599 |
| g__Eubacterium_oxidoreducens_group | 0.1097 | 0.04396 | 0.01053 | 0.01372 | 0.0008599 |
| g__Eubacterium_nodatum_group | 0.08516 | 0.05619 | 0.003042 | 0.004792 | 0.0008446 |
| g__UCG-009 | 0.01521 | 0.01448 | 0.000234 | 0.0006618 | 0.0007949 |
| g__GCA-900066575 | 0.1453 | 0.1398 | 0.001872 | 0.003318 | 0.0007781 |
| g__UBA1819 | 0.1032 | 0.1004 | 0.003977 | 0.006203 | 0.0007781 |
| g__Lachnospiraceae_UCG-006 | 1.958 | 1.696 | 0.0004679 | 0.0008664 | 0.0006754 |
| g__Oscillibacter | 0.02457 | 0.01413 | 0.0007019 | 0.001393 | 0.0006754 |
| g__A2 | 0.2176 | 0.2829 | 0 | 0 | 0.0004099 |
| g__Family_XIII_UCG-001 | 0.04258 | 0.0473 | 0 | 0 | 0.0004099 |
| g__UCG-007 | 0.01872 | 0.01368 | 0 | 0 | 0.0004054 |

Notes: Species name is the name of the species; Mean (%) is the mean relative abundance of the species in different subgroups; Sd (%) is the standard deviation; *p* value is the probability of false positives, which is a commonly used decision value in statistics. M, model group; WKY, normal control group.

**Supplementary Table 4. Callback serum metabolites of EA**

| Metabolite | Formula | Mode | M/Z |
| --- | --- | --- | --- |
| 3'-Hydroxyamobarbital | C11H18N2O4 | pos | 243.1344019 |
| Glycocholic Acid | C26H43NO6 | pos | 430.2961079 |
| GPCho(20:5/2:0) | C30H50NO8P | pos | 584.3361757 |
| 4-Amino-1-piperidinecarboxylic acid | C6H12N2O2 | pos | 109.0765383 |
| 2-Amino-4-methylpyridine | C6H8N2 | pos | 109.0765337 |
| Glycylvaline | C7H14N2O3 | pos | 157.0974372 |
| L-Rhamnulose | C6H12O5 | pos | 392.1496002 |
| Dimboa glucoside | C15H19NO10 | pos | 338.0880749 |
| Benzoic Acid | C7H6O2 | pos | 105.0340026 |
| Hovenoside D | C57H92O26 | pos | 597.2963547 |
| 5-Hydroxymethyl-4-methyluracil | C6H8N2O3 | pos | 157.061039 |
| PG(i-12:0/i-12:0) | C30H59O10P | pos | 652.4140403 |
| L-4-Chlorotryptophan | C11H11ClN2O2 | pos | 239.0586717 |
| 4-Ethylphenylsulfate | C8H10O4S | pos | 247.0016666 |
| Azimexon | C9H14N4O | pos | 239.0891228 |
| Patulin | C7H6O4 | pos | 187.0604614 |
| 2,5-Furandicarboxaldehyde | C6H4O3 | pos | 125.0236865 |
| Hexyl glucoside | C12H24O6 | pos | 247.1545204 |
| 6-Hydroxyoctadecanoic acid | C18H36O3 | pos | 318.3009524 |
| Phenylbutyrylglutamine | C15H20N2O4 | pos | 315.1342628 |
| Methyltestosterone | C20H30O2 | pos | 303.2325571 |
| Anonaine | C17H15NO2 | pos | 569.1852156 |
| Suspensolide F | C21H34O12 | pos | 501.1977178 |
| Moexipril | C27H34N2O7 | pos | 481.233863 |
| 1-Arachidonoylglycerol | C23H38O4 | pos | 361.2743238 |
| PS(22:0/15:0) | C43H84NO10P | pos | 844.548036 |
| PC(20:2(11Z,14Z)/15:0) | C43H82NO8P | pos | 794.5719877 |
| (2R,5R)-2-[6-(Cyclopentylamino)-8-(methylamino)purin-9-yl]-5-(hydroxymethyl)oxolane-3,4-diol | C16H24N6O4 | pos | 397.2172816 |
| PC(O-18:0/0:0) | C26H56NO6P | pos | 532.3748828 |
| 4-Piperidinone, 1-hydroxy-2,2,6,6-tetramethyl- | C9H17NO2 | pos | 172.1333043 |
| 19(S)-HETE | C20H32O3 | pos | 343.2247957 |
| 5,6-Methylenedioxy-2-aminoindane | C10H11NO2 | pos | 219.1132746 |
| 8(R)-HETE | C20H32O3 | pos | 303.2320876 |
| Octanol | C8H18O | pos | 172.1697829 |
| PC(6:2(3E,5E)/14:2(11E,13E)) | C28H48NO8P | pos | 558.3197014 |
| LysoPA(18:2(9Z,12Z)/0:0) | C21H39O7P | pos | 457.2335514 |
| PGB2 | C20H30O4 | pos | 335.2224435 |
| 3,4-dihydroxyphenylacetic Acid | C8H8O4 | pos | 169.0497943 |
| Estrone | C18H22O2 | pos | 315.134645 |
| 3,4-Dihydroxymandelic Acid | C8H8O5 | pos | 167.0341538 |
| 1-(5-Hydroxy-2-oxo-2,3-dihydroimidazol-4-yl)urea | C4H6N4O3 | pos | 222.0587614 |
| Epsilon-Caprolactone | C6H10O2 | pos | 229.1438951 |
| Phenyl-Alanine | C9H11NO2 | pos | 198.112917 |
| Sophoramine | C15H20N2O | pos | 227.154849 |
| 4-Imidazolone-5-propionic acid | C6H8N2O3 | pos | 157.061039 |
| Formiminoglutamic acid | C6H10N2O4 | pos | 157.0610554 |
| Astromicin | C17H35N5O6 | pos | 388.2554083 |
| 3-Hydroxymonoethylglycinexylidide | C12H18N2O2 | pos | 264.1712346 |
| N6-Acetyl-L-lysine | C8H16N2O3 | pos | 171.1132792 |
| 2,3,4,5-Tetrahydro-2-pyridinecarboxylic acid | C6H9NO2 | pos | 169.0974149 |
| Lotaustralin | C11H19NO6 | pos | 244.1182779 |
| Oxypurinol | C5H4N4O2 | pos | 153.0408933 |
| Steviol | C20H30O3 | pos | 319.2272607 |
| LL-2,6-Diaminopimelic Acid | C7H14N2O4 | pos | 155.0816889 |
| Niacinamide | C6H6N2O | pos | 123.0556831 |
| (2,5-Dihydroxy-1H-pyrrol-3-yl) prop-2-enoate | C7H7NO4 | pos | 169.0358387 |
| Stachyose | C24H42O21 | pos | 689.213038 |
| L-Glutamic Acid | C5H9NO4 | pos | 148.0605495 |
| 1-Methylnicotinamide | C7H9N2O+ | pos | 137.0711024 |
| Ineketone | C20H30O3 | pos | 336.251459 |
| N-[2-(3,4-Dihydroxyphenyl)ethyl]icosa-5,8,11,14-tetraenamide | C28H41NO3 | pos | 440.3146747 |
| PC(18:0/20:4(5Z,8Z,11Z,14Z)-OH(19S)) | C46H84NO9P | pos | 848.5792899 |
| 1-(6Z,9Z,12Z-octadecatrienoyl)-glycero-3-phosphate | C21H37O7P | pos | 433.2335223 |
| PS(20:0/20:4(5Z,8Z,11Z,14Z)) | C46H82NO10P | pos | 822.5671468 |
| Traumatic acid | C12H20O4 | pos | 229.1438533 |
| Threonic Acid | C4H8O5 | neg | 135.0293839 |
| Valylglutamic acid | C10H18N2O5 | neg | 283.0688903 |
| 2-Methoxyacetaminophen sulfate | C9H11NO6S | neg | 242.0131467 |
| Methionyl-Histidine | C11H18N4O3S | neg | 321.0768077 |
| Pimonidazole | C11H18N4O3 | neg | 275.1143168 |
| Citrinin | C13H14O5 | neg | 249.0771846 |
| 5-Phenyl-1,3-oxazinane-2,4-dione | C10H9NO3 | neg | 226.0278144 |
| Aminofructose 6-phosphate | C6H14NO8P | neg | 294.0158237 |
| Albifylline | C13H20N4O3 | neg | 301.1298754 |
| 3-Butylidene-1(3H)-isobenzofuranone | C12H12O2 | neg | 233.0820764 |
| Ethyl maltol | C7H8O3 | neg | 185.0452612 |
| 3-Allylphenol sulfate | C9H10O4S | neg | 213.0226443 |
| 9,10,13-TriHOME | C18H34O5 | neg | 329.233884 |
| (11R,16S)-misoprostol | C22H38O5 | neg | 381.264838 |
| Cannabidiolic acid | C22H30O4 | neg | 403.2108823 |
| PA(i-14:0/20:3(6,8,11)-OH(5)) | C37H67O9P | neg | 685.4475114 |
| Allylestrenol | C21H32O | neg | 345.243859 |
| PE-NMe2(18:2(9Z,12Z)/20:3(8Z,11Z,14Z)) | C45H80NO8P | neg | 838.5633953 |
| Carboprost | C21H36O5 | neg | 389.2317364 |
| Isolithocholic acid | C24H40O3 | neg | 375.2909981 |
| PA(8:0/10:0) | C21H41O8P | neg | 473.2322027 |
| Prostaglandin G2 2-glyceryl Ester | C23H38O8 | neg | 477.2246492 |
| Docosanedioic acid | C22H42O4 | neg | 369.3016059 |
| Menthone lactone | C10H18O2 | neg | 339.2544022 |
| Octadecyl fumarate | C22H40O4 | neg | 349.2752411 |
| Manoalide | C25H36O5 | neg | 415.2474946 |
| 20, 22-Dihydrodigoxigenin | C23H36O5 | neg | 413.2319477 |
| Deoxycorticosterone acetate | C23H32O4 | neg | 353.2126888 |
| Mevastatin | C23H34O5 | neg | 411.2160961 |
| Cannabigerolate | C22H32O4 | neg | 341.2116679 |
| Dihydrocortisol | C21H32O5 | neg | 385.2003321 |
| Prostaglandin A1 | C20H32O4 | neg | 335.2233333 |
| (+/-)-11,12-Dihydroxy-5Z,8Z,14Z,17Z-eicosatetraenoic acid | C20H32O4 | neg | 381.226723 |
| 9,10-Dihome | C18H34O4 | neg | 313.2389472 |
| 20-Hydroxy-leukotriene B4 | C20H32O5 | neg | 333.2075512 |
| (5Z)-7-[(1R,2E)-2-[(3S)-3-hydroxyoctylidene]-3-oxocyclopentyl]hept-5-enoylcarnitine | C27H45NO6 | neg | 516.274116 |
| Cromakalim | C16H18N2O3 | neg | 267.1143577 |
| Arg-Thr-Lys-Arg | C22H45N11O6 | neg | 580.3276621 |
| Polyporusterone G | C28H44O5 | neg | 505.3183642 |
| Contignasterol | C29H48O7 | neg | 507.3340176 |
| (3S,5R,6R,7E)-3,5,6-Trihydroxy-7-megastigmen-9-one | C13H22O4 | neg | 287.1504715 |
| (+)-Lysergic acid | C16H16N2O2 | neg | 313.1196574 |
| 4alpha-carboxy-5alpha-cholesta-8,24-dien-3beta-ol | C28H43O3- | neg | 462.2874977 |
| Anisatin | C15H20O8 | neg | 349.0888641 |
| Alpha-Carboxy-delta-decalactone | C11H18O4 | neg | 259.1192572 |
| Pimelic Acid | C7H12O4 | neg | 141.0552335 |
| 3,5-Dichloro-2-hydroxybenzenesulfonic acid | C6H4Cl2O4S | neg | 240.9138229 |
| Nafazatrom | C16H16N2O2 | neg | 313.1201129 |
| 3-ethylphenyl Sulfate | C8H10O4S | neg | 201.0226062 |
| Gentianine | C10H9NO2 | neg | 210.0326088 |
| Harmalol | C12H12N2O | neg | 237.0438981 |
| TETRAHYDROURIDINE | C9H16N2O6 | neg | 283.0688273 |
| HBOA trihexose | C8H15NO2 | neg | 202.1083472 |
| Nalidixic acid | C12H12N2O3 | neg | 231.0776724 |
| 5,6-Dihydroxyprostaglandin F1a | C20H36O7 | neg | 387.2398972 |
| Marindinin | C14H16O3 | neg | 253.0835742 |
| Humilixanthin | C14H18N2O7 | neg | 307.091776 |
| Xanthosine | C10H12N4O6 | neg | 283.069071 |
| Icariside B8 | C19H32O8 | neg | 387.203175 |
| Methylmalonic Acid | C4H6O4 | neg | 117.0188005 |
| Succinic Acid | C4H6O4 | neg | 117.0187919 |
| Glucomannan | C24H42O21 | neg | 701.1937772 |
| 12-KETE | C20H30O3 | neg | 317.2126248 |
| 13-HODE | C18H32O3 | neg | 295.2281361 |
| 10-HDoHE | C22H32O3 | neg | 343.2282719 |
| GPSer(2:0/18:3) | C26H44NO10P | neg | 560.2644092 |
| Prostaglandin E3 | C20H30O5 | neg | 331.1917542 |
| PS(PGJ2/20:5(5Z,8Z,11Z,14Z,17Z)) | C46H70NO12P | pos | 860.4752927 |
| PI(6 keto-PGF1alpha/18:3(9Z,12Z,15Z)) | C47H79O17P | pos | 474.257631 |
| PI(TXB2/18:3(9Z,12Z,15Z)) | C47H79O17P | pos | 474.2579157 |
| Icofungipen | C7H11NO2 | pos | 106.0656208 |
| Bz-Arg-OEt | C15H22N4O3 | pos | 676.3532056 |
| 4-Methylumbelliferone | C10H8O3 | pos | 194.0816369 |
| Porfimer Sodium | C68H74N8O11 | pos | 601.2691642 |
| CDP-DG(PGJ2/i-19:0) | C51H85N3O17P2 | pos | 537.7710845 |
| SM(d18:1/14:0) | C37H75N2O6P | pos | 675.5454486 |
| SM(d16:1/PGF1alpha) | C41H79N2O9P | pos | 792.5910331 |
| LysoPC(24:0/0:0) | C32H66NO7P | pos | 608.466874 |
| PS(18:1(11Z)/22:5(7Z,10Z,13Z,16Z,19Z)) | C46H78NO10P | pos | 858.5258537 |
| N-Acetyl-L-glutamic acid | C7H11NO5 | pos | 231.0970793 |
| PS(18:1(12Z)-O(9S,10R)/22:0) | C46H86NO11P | pos | 898.5573965 |
| CDP-DG(20:5(7Z,9Z,11E,13E,17Z)-3OH(5,6,15)/18:2(9Z,11Z)) | C50H79N3O18P2 | pos | 558.7308842 |
| PGP(22:5(4Z,7Z,10Z,13Z,16Z)/22:5(7Z,10Z,13Z,16Z,19Z)) | C50H80O13P2 | pos | 487.247219 |
| Corchoroside B | C29H42O8 | pos | 518.2836679 |
| Mepartricin | C60H88N2O19 | pos | 582.2959407 |
| PI(LTE4/18:1(9Z)) | C50H86NO16PS | pos | 532.7613559 |
| S-(PGA1)-glutathione | C30H49N3O10S | pos | 676.3535292 |
| Thiodiacetic acid | C4H6O4S | pos | 192.0327361 |
| PE(15:0/22:4(7Z,10Z,13Z,16Z)) | C42H76NO8P | pos | 776.5216516 |
| (3s)-3-(Benzyloxy)-L-Aspartic Acid | C11H13NO5 | neg | 260.0545254 |
| PIP(20:4(8Z,11Z,14Z,17Z)/18:1(9Z)) | C47H82O16P2 | neg | 945.4931507 |
| Mibefradil | C29H38FN3O3 | neg | 516.2686969 |
| CDP-DG(20:4(8Z,11Z,14Z,17Z)-2OH(5S,6R)/18:0) | C50H85N3O17P2 | neg | 529.7562972 |
| CDP-DG(PGJ2/20:2(11Z,14Z)) | C52H83N3O17P2 | neg | 540.7473562 |
| CTAP-III | C51H71N13O11S2 | neg | 551.7385216 |
| Vanilloyl glucose | C14H18O9 | neg | 351.0704057 |
| (3Z,6Z)-3,6-Nonadienal | C9H14O | neg | 183.1022455 |
| 12,13-Epoxy-9-Octadecenoic Acid | C18H32O3 | neg | 295.2282361 |
| 7-Epijasmonic acid | C12H18O3 | neg | 209.1165838 |
| 3a,6b,7a,12a-Tetrahydroxy-5b-cholanoic acid | C24H40O6 | neg | 405.2654412 |
| 2-Nonen-1-ol | C9H18O | neg | 141.1279555 |
| PE-NMe2(20:4(5Z,8Z,11Z,14Z)/16:0) | C43H78NO8P | neg | 766.5420993 |
| PE(18:1(9Z)/20:1(11Z)) | C43H82NO8P | neg | 792.5573316 |
| PI(6 keto-PGF1alpha/18:3(6Z,9Z,12Z)) | C47H79O17P | neg | 945.4935773 |
| PS(20:5(5Z,8Z,11Z,14Z,17Z)/PGJ2) | C46H70NO12P | neg | 858.4616058 |
| 3b,7a-Dihydroxy-5b-cholanoic acid | C24H40O4 | neg | 391.2861144 |
| MG(16:1(9Z)/0:0/0:0) | C19H36O4 | neg | 309.244041 |
| Alpha-Carboxy-delta-nonalactone | C10H16O4 | neg | 181.0866111 |
| 3-[4-(1-Imidazolylmethyl)phenyl]-2-propenoic acid | C13H12N2O2 | neg | 263.0600403 |
| Quillaic acid 3-[galactosyl-(1->2)-[rhamnosyl-(1->3)]-glucuronide] 28-[xylosyl-(1->4)-rhamnosyl-(1->2)-[rhamnosyl-(1->3)]-4acetyl-fucosyl] ester | C73H114O37 | neg | 790.3464939 |
| P-Cresol glucuronide | C13H16O7 | neg | 283.0828658 |
| CDP-DG(20:4(6Z,8E,10E,14Z)-2OH(5S,12R)/18:0) | C50H85N3O17P2 | neg | 529.7563177 |
| CDP-DG(20:2(11Z,14Z)/PGJ2) | C52H83N3O17P2 | neg | 540.7474079 |
| PS(22:6(4Z,7Z,10Z,13Z,16Z,19Z)/5-iso PGF2VI) | C46H72NO13P | neg | 858.4613055 |
| Phenylacetylglycine | C10H11NO3 | neg | 192.0663869 |
| Eptifibatide | C35H49N11O9S2 | neg | 868.2672652 |
| (1alpha,3beta,20S,22R,24S,25S)-Pubescenin | C34H52O10 | neg | 619.3485523 |

Notes: Metabolite: name of the identified metabolite; Formula: chemical formula of the metabolite; Mode: ionic mode, pos positive, neg negative mode; M/Z: mass-to-charge ratio, the ratio of the mass of a charged ion to its charge.

**Supplementary Table 5. Callback serum metabolites of MA**

| Metabolite | Formula | Mode | M/Z |
| --- | --- | --- | --- |
| 3'-Hydroxyamobarbital | C11H18N2O4 | pos | 243.1344019 |
| GPCho(18:3/2:0) | C28H50NO8P | pos | 560.3365972 |
| GPCho(20:5/2:0) | C30H50NO8P | pos | 584.3361757 |
| Gaboxadol | C6H8N2O2 | pos | 123.0557077 |
| L-Rhamnulose | C6H12O5 | pos | 392.1496002 |
| PI(5-iso PGF2VI/22:5(7Z,10Z,13Z,16Z,19Z)) | C49H79O16P | pos | 489.2581332 |
| Benzoic Acid | C7H6O2 | pos | 105.0340026 |
| Hovenoside D | C57H92O26 | pos | 597.2963547 |
| 5-Hydroxymethyl-4-methyluracil | C6H8N2O3 | pos | 157.061039 |
| 4-Ethylphenylsulfate | C8H10O4S | pos | 247.0016666 |
| Azimexon | C9H14N4O | pos | 239.0891228 |
| Patulin | C7H6O4 | pos | 187.0604614 |
| Nornicotine | C9H12N2 | pos | 314.2332612 |
| 2,5-Furandicarboxaldehyde | C6H4O3 | pos | 125.0236865 |
| Phenylbutyrylglutamine | C15H20N2O4 | pos | 315.1342628 |
| 2-O-ethyl PAF C-16 | C26H56NO6P | pos | 510.3930757 |
| PE-NMe2(20:4(8Z,11Z,14Z,17Z)/20:5(5Z,8Z,11Z,14Z,17Z)) | C47H76NO8P | pos | 846.5630626 |
| Methyltestosterone | C20H30O2 | pos | 303.2325571 |
| PC(16:0/0:0) | C24H50NO7P | pos | 518.3235135 |
| Anonaine | C17H15NO2 | pos | 569.1852156 |
| Suspensolide F | C21H34O12 | pos | 501.1977178 |
| Moexipril | C27H34N2O7 | pos | 481.233863 |
| 1-Arachidonoylglycerol | C23H38O4 | pos | 361.2743238 |
| PS(22:0/15:0) | C43H84NO10P | pos | 844.548036 |
| PC(20:2(11Z,14Z)/15:0) | C43H82NO8P | pos | 794.5719877 |
| PC(O-18:0/0:0) | C26H56NO6P | pos | 532.3748828 |
| 19(S)-HETE | C20H32O3 | pos | 343.2247957 |
| 5,6-Methylenedioxy-2-aminoindane | C10H11NO2 | pos | 219.1132746 |
| 8(R)-HETE | C20H32O3 | pos | 303.2320876 |
| CDP-DG(22:6(4Z,7Z,10Z,12E,16Z,19Z)-OH(14)/i-19:0) | C53H87N3O16P2 | pos | 553.7801065 |
| 2,5-Dihydroxy-1-octadec-9-enoyloxypyrrole-3-sulfonic acid | C22H37NO7S | pos | 523.2447965 |
| PC(6:2(3E,5E)/14:2(11E,13E)) | C28H48NO8P | pos | 558.3197014 |
| LysoPA(18:2(9Z,12Z)/0:0) | C21H39O7P | pos | 457.2335514 |
| Decanoylcarnitine | C17H33NO4 | pos | 316.2489943 |
| PGB2 | C20H30O4 | pos | 335.2224435 |
| Cantharidin | C10H12O4 | pos | 229.1074863 |
| 3,4-dihydroxyphenylacetic Acid | C8H8O4 | pos | 169.0497943 |
| Estrone | C18H22O2 | pos | 315.134645 |
| 5-Methoxyindoleacetate | C11H11NO3 | pos | 188.0709581 |
| 3,4-Dihydroxymandelic Acid | C8H8O5 | pos | 167.0341538 |
| L-Octanoylcarnitine | C15H29NO4 | pos | 288.2173621 |
| 1-(5-Hydroxy-2-oxo-2,3-dihydroimidazol-4-yl)urea | C4H6N4O3 | pos | 222.0587614 |
| Epsilon-Caprolactone | C6H10O2 | pos | 229.1438951 |
| Phenyl-Alanine | C9H11NO2 | pos | 198.112917 |
| Sophoramine | C15H20N2O | pos | 227.154849 |
| N-Lauroyl Glutamine | C17H32N2O4 | pos | 328.2338813 |
| N(2)-phenylacetyl-L-glutaminate | C13H16N2O4 | pos | 287.0984437 |
| CDP-DG(i-19:0/22:6(4Z,7Z,10Z,12E,16Z,19Z)-OH(14)) | C53H87N3O16P2 | pos | 553.7798993 |
| 4-Imidazolone-5-propionic acid | C6H8N2O3 | pos | 157.061039 |
| Formiminoglutamic acid | C6H10N2O4 | pos | 157.0610554 |
| 3-Hydroxymonoethylglycinexylidide | C12H18N2O2 | pos | 264.1712346 |
| N6-Acetyl-L-lysine | C8H16N2O3 | pos | 171.1132792 |
| Lotaustralin | C11H19NO6 | pos | 244.1182779 |
| Oxypurinol | C5H4N4O2 | pos | 153.0408933 |
| Steviol | C20H30O3 | pos | 319.2272607 |
| LL-2,6-Diaminopimelic Acid | C7H14N2O4 | pos | 155.0816889 |
| Niacinamide | C6H6N2O | pos | 123.0556831 |
| (2,5-Dihydroxy-1H-pyrrol-3-yl) prop-2-enoate | C7H7NO4 | pos | 169.0358387 |
| Stachyose | C24H42O21 | pos | 689.213038 |
| L-Glutamic Acid | C5H9NO4 | pos | 148.0605495 |
| 1-Methylnicotinamide | C7H9N2O+ | pos | 137.0711024 |
| Inolin | C19H23NO5 | pos | 363.1912539 |
| PC(P-18:0/0:0) | C26H54NO6P | pos | 508.3772606 |
| PC(18:1(6Z)/0:0) | C26H52NO7P | pos | 544.3398188 |
| PC(18:0/20:4(5Z,8Z,11Z,14Z)-OH(19S)) | C46H84NO9P | pos | 848.5792899 |
| 1-(6Z,9Z,12Z-octadecatrienoyl)-glycero-3-phosphate | C21H37O7P | pos | 433.2335223 |
| PS(20:0/20:4(5Z,8Z,11Z,14Z)) | C46H82NO10P | pos | 822.5671468 |
| 2,4,6(1H,3H,5H)-Pyrimidinetrione, 5-ethyl-5-(3-hydroxy-1-methylbutyl)- | C11H18N2O4 | pos | 287.0984653 |
| Valylglutamic acid | C10H18N2O5 | neg | 283.0688903 |
| 2-Methoxyacetaminophen sulfate | C9H11NO6S | neg | 242.0131467 |
| Methionyl-Histidine | C11H18N4O3S | neg | 321.0768077 |
| B-Raf inhibitor | C29H31F3N6O2 | neg | 573.2172933 |
| Pimonidazole | C11H18N4O3 | neg | 275.1143168 |
| Citrinin | C13H14O5 | neg | 249.0771846 |
| P-Toluenesulfonic acid | C7H8O3S | neg | 217.0176038 |
| 12(R)-HETE | C20H32O3 | neg | 319.2283327 |
| Albifylline | C13H20N4O3 | neg | 301.1298754 |
| 1-(2,2-Difluoroethyl)pyrrolidine-3,4-dicarboxylic acid | C8H11F2NO4 | neg | 491.127321 |
| 3-Butylidene-1(3H)-isobenzofuranone | C12H12O2 | neg | 233.0820764 |
| Ethyl maltol | C7H8O3 | neg | 185.0452612 |
| 3-Allylphenol sulfate | C9H10O4S | neg | 213.0226443 |
| 11b-PGF2a | C20H34O5 | neg | 353.2339765 |
| 9,10,13-TriHOME | C18H34O5 | neg | 329.233884 |
| Prostaglandin F1a | C20H36O5 | neg | 337.2391242 |
| (11R,16S)-misoprostol | C22H38O5 | neg | 381.264838 |
| PA(i-14:0/20:3(6,8,11)-OH(5)) | C37H67O9P | neg | 685.4475114 |
| Allylestrenol | C21H32O | neg | 345.243859 |
| PE(20:4(5Z,8Z,11Z,14Z)-OH(16R)/P-18:0) | C43H78NO8P | neg | 812.5477005 |
| PE-NMe2(18:2(9Z,12Z)/20:3(8Z,11Z,14Z)) | C45H80NO8P | neg | 838.5633953 |
| Carboprost | C21H36O5 | neg | 389.2317364 |
| Isolithocholic acid | C24H40O3 | neg | 375.2909981 |
| Olvanil | C26H43NO3 | neg | 438.299891 |
| 10-Hydroxyheptadecanoylcarnitine | C24H47NO5 | neg | 464.3157013 |
| PA(8:0/10:0) | C21H41O8P | neg | 473.2322027 |
| Eicosapentaenoic Acid | C20H30O2 | neg | 301.2177937 |
| Prostaglandin G2 2-glyceryl Ester | C23H38O8 | neg | 477.2246492 |
| Octadecyl fumarate | C22H40O4 | neg | 349.2752411 |
| Manoalide | C25H36O5 | neg | 415.2474946 |
| 20, 22-Dihydrodigoxigenin | C23H36O5 | neg | 413.2319477 |
| Deoxycorticosterone acetate | C23H32O4 | neg | 353.2126888 |
| 4-(4-Methylcyclohexyl)-4-oxobutanoic acid | C11H18O3 | neg | 179.1073007 |
| Mevastatin | C23H34O5 | neg | 411.2160961 |
| Dihydrocortisol | C21H32O5 | neg | 385.2003321 |
| Strophanthidin | C23H32O6 | neg | 403.2109866 |
| Prostaglandin A1 | C20H32O4 | neg | 335.2233333 |
| (+/-)-11,12-Dihydroxy-5Z,8Z,14Z,17Z-eicosatetraenoic acid | C20H32O4 | neg | 381.226723 |
| 9,10-Dihome | C18H34O4 | neg | 313.2389472 |
| Jurubine | C33H57NO8 | neg | 632.3585633 |
| (+/-)-Menthyl acetate | C12H22O2 | neg | 243.1602166 |
| 20-Hydroxy-leukotriene B4 | C20H32O5 | neg | 333.2075512 |
| (5Z)-7-[(1R,2E)-2-[(3S)-3-hydroxyoctylidene]-3-oxocyclopentyl]hept-5-enoylcarnitine | C27H45NO6 | neg | 516.274116 |
| Cromakalim | C16H18N2O3 | neg | 267.1143577 |
| 2-Hydroxycampholonic acid | C10H16O4 | neg | 399.2030612 |
| Arg-Thr-Lys-Arg | C22H45N11O6 | neg | 580.3276621 |
| Alprostadil | C20H34O5 | neg | 335.2233836 |
| (3S,5R,6R,7E)-3,5,6-Trihydroxy-7-megastigmen-9-one | C13H22O4 | neg | 287.1504715 |
| (+)-Lysergic acid | C16H16N2O2 | neg | 313.1196574 |
| Senkyunolide N | C12H18O4 | neg | 271.119236 |
| Anisatin | C15H20O8 | neg | 349.0888641 |
| Alpha-Carboxy-delta-decalactone | C11H18O4 | neg | 259.1192572 |
| Pimelic Acid | C7H12O4 | neg | 141.0552335 |
| 3,5-Dichloro-2-hydroxybenzenesulfonic acid | C6H4Cl2O4S | neg | 240.9138229 |
| Nafazatrom | C16H16N2O2 | neg | 313.1201129 |
| Eremopetasin sulfoxide | C19H26O4S | neg | 331.1405822 |
| 2-(4-(3-(4-Acetyl-3-hydroxy-2-propylphenoxy)propoxy)phenoxy)acetic acid | C22H26O7 | neg | 401.1575994 |
| 3-ethylphenyl Sulfate | C8H10O4S | neg | 201.0226062 |
| TETRAHYDROURIDINE | C9H16N2O6 | neg | 283.0688273 |
| HBOA trihexose | C8H15NO2 | neg | 202.1083472 |
| 5,6-Dihydroxyprostaglandin F1a | C20H36O7 | neg | 387.2398972 |
| Thyrotropin-releasing factor | C16H22N6O4 | neg | 769.3395722 |
| Carboxyibuprofen | C13H16O4 | neg | 235.0976474 |
| Dimerum acid | C22H36N4O8 | neg | 505.2293971 |
| S-(Formylmethyl)glutathione | C12H19N3O7S | neg | 386.0422365 |
| Valine-betaxanthin | C14H18N2O6 | neg | 309.1074368 |
| Fasoracetam | C10H16N2O2 | neg | 241.1196676 |
| N-Acetylaspartylglutamic acid | C11H16N2O8 | neg | 303.0848506 |
| Marindinin | C14H16O3 | neg | 253.0835742 |
| Humilixanthin | C14H18N2O7 | neg | 307.091776 |
| Xanthosine | C10H12N4O6 | neg | 283.069071 |
| Icariside B8 | C19H32O8 | neg | 387.203175 |
| Methylmalonic Acid | C4H6O4 | neg | 117.0188005 |
| MG(5-iso PGF2VI/0:0/0:0) | C21H36O7 | neg | 421.2220907 |
| Succinic Acid | C4H6O4 | neg | 117.0187919 |
| Glucomannan | C24H42O21 | neg | 701.1937772 |
| 12-KETE | C20H30O3 | neg | 317.2126248 |
| 13-HODE | C18H32O3 | neg | 295.2281361 |
| 10-HDoHE | C22H32O3 | neg | 343.2282719 |
| GPSer(2:0/18:3) | C26H44NO10P | neg | 560.2644092 |
| Prostaglandin E3 | C20H30O5 | neg | 331.1917542 |
| Aspartic Acid | C4H7NO4 | neg | 132.0297089 |
| L-Glutamate | C5H9NO4 | neg | 146.0453802 |
| N-Acetylneuraminic Acid | C11H19NO9 | neg | 308.099313 |
| Tetrahydrocortisol | C21H34O5 | neg | 387.2161734 |
| PS(PGJ2/20:5(5Z,8Z,11Z,14Z,17Z)) | C46H70NO12P | pos | 860.4752927 |
| PI(6 keto-PGF1alpha/18:3(9Z,12Z,15Z)) | C47H79O17P | pos | 474.257631 |
| PI(TXB2/18:3(9Z,12Z,15Z)) | C47H79O17P | pos | 474.2579157 |
| Bz-Arg-OEt | C15H22N4O3 | pos | 676.3532056 |
| 4-Methylumbelliferone | C10H8O3 | pos | 194.0816369 |
| Porfimer Sodium | C68H74N8O11 | pos | 601.2691642 |
| CDP-DG(PGJ2/i-19:0) | C51H85N3O17P2 | pos | 537.7710845 |
| Beta-Alanyl-L-arginine | C9H19N5O3 | pos | 245.1498984 |
| PS(18:1(11Z)/22:5(7Z,10Z,13Z,16Z,19Z)) | C46H78NO10P | pos | 858.5258537 |
| Gonal | C18H28O | pos | 278.248179 |
| N-Acetyl-L-glutamic acid | C7H11NO5 | pos | 231.0970793 |
| CDP-DG(20:5(7Z,9Z,11E,13E,17Z)-3OH(5,6,15)/18:2(9Z,11Z)) | C50H79N3O18P2 | pos | 558.7308842 |
| PGP(22:5(4Z,7Z,10Z,13Z,16Z)/22:5(7Z,10Z,13Z,16Z,19Z)) | C50H80O13P2 | pos | 487.247219 |
| Corchoroside B | C29H42O8 | pos | 518.2836679 |
| Mepartricin | C60H88N2O19 | pos | 582.2959407 |
| CDP-DG(LTE4/18:0) | C53H90N4O18P2S | pos | 583.2853906 |
| PI(LTE4/18:1(9Z)) | C50H86NO16PS | pos | 532.7613559 |
| S-(PGA1)-glutathione | C30H49N3O10S | pos | 676.3535292 |
| CDP-DG(18:0/LTE4) | C53H90N4O18P2S | neg | 581.2704432 |
| 4-Vinylphenol sulfate | C8H8O4S | neg | 245.0128268 |
| (3s)-3-(Benzyloxy)-L-Aspartic Acid | C11H13NO5 | neg | 260.0545254 |
| PIP(20:4(8Z,11Z,14Z,17Z)/18:1(9Z)) | C47H82O16P2 | neg | 945.4931507 |
| Mibefradil | C29H38FN3O3 | neg | 516.2686969 |
| CDP-DG(20:4(8Z,11Z,14Z,17Z)-2OH(5S,6R)/18:0) | C50H85N3O17P2 | neg | 529.7562972 |
| CDP-DG(PGJ2/20:2(11Z,14Z)) | C52H83N3O17P2 | neg | 540.7473562 |
| CTAP-III | C51H71N13O11S2 | neg | 551.7385216 |
| Vanilloyl glucose | C14H18O9 | neg | 351.0704057 |
| O-Cresol | C7H8O | neg | 107.0496525 |
| P-Tolyl Sulfate | C7H8O4S | neg | 187.0067906 |
| 12,13-Epoxy-9-Octadecenoic Acid | C18H32O3 | neg | 295.2282361 |
| 3a,6b,7a,12a-Tetrahydroxy-5b-cholanoic acid | C24H40O6 | neg | 405.2654412 |
| PI(6 keto-PGF1alpha/18:3(6Z,9Z,12Z)) | C47H79O17P | neg | 945.4935773 |
| PS(20:5(5Z,8Z,11Z,14Z,17Z)/PGJ2) | C46H70NO12P | neg | 858.4616058 |
| 3b,7a-Dihydroxy-5b-cholanoic acid | C24H40O4 | neg | 391.2861144 |
| MG(16:1(9Z)/0:0/0:0) | C19H36O4 | neg | 309.244041 |
| Gamma-Glutamylglutamine | C10H17N3O6 | neg | 274.1049311 |
| Quillaic acid 3-[galactosyl-(1->2)-[rhamnosyl-(1->3)]-glucuronide] 28-[xylosyl-(1->4)-rhamnosyl-(1->2)-[rhamnosyl-(1->3)]-4acetyl-fucosyl] ester | C73H114O37 | neg | 790.3464939 |
| P-Cresol glucuronide | C13H16O7 | neg | 283.0828658 |
| CDP-DG(20:4(6Z,8E,10E,14Z)-2OH(5S,12R)/18:0) | C50H85N3O17P2 | neg | 529.7563177 |
| CDP-DG(20:2(11Z,14Z)/PGJ2) | C52H83N3O17P2 | neg | 540.7474079 |
| PS(22:6(4Z,7Z,10Z,13Z,16Z,19Z)/5-iso PGF2VI) | C46H72NO13P | neg | 858.4613055 |
| 3-hydroxy-3-(3-hydroxyphenyl)propanoic acid-O-sulphate | C9H10O7S | neg | 242.9970755 |
| Phenylacetylglycine | C10H11NO3 | neg | 192.0663869 |
| Sorbinil | C11H9FN2O3 | neg | 273.0080838 |
| 3-[4-(sulfooxy)phenyl]propanoic acid | C9H10O6S | neg | 245.0128725 |

Notes: Metabolite: name of the identified metabolite; Formula: chemical formula of the metabolite; Mode: ionic mode, pos positive, neg negative mode; M/Z: mass-to-charge ratio, the ratio of the mass of a charged ion to its charge.

**Supplementary Table 6. Callback serum metabolites of EA and MA**

| Metabolite | Formula | Mode | M/Z |
| --- | --- | --- | --- |
| 3'-Hydroxyamobarbital | C11H18N2O4 | pos | 243.1344019 |
| PS(PGJ2/20:5(5Z,8Z,11Z,14Z,17Z)) | C46H70NO12P | pos | 860.4752927 |
| PI(6 keto-PGF1alpha/18:3(9Z,12Z,15Z)) | C47H79O17P | pos | 474.257631 |
| PI(TXB2/18:3(9Z,12Z,15Z)) | C47H79O17P | pos | 474.2579157 |
| GPCho(20:5/2:0) | C30H50NO8P | pos | 584.3361757 |
| L-Rhamnulose | C6H12O5 | pos | 392.1496002 |
| Bz-Arg-OEt | C15H22N4O3 | pos | 676.3532056 |
| Benzoic Acid | C7H6O2 | pos | 105.0340026 |
| 4-Methylumbelliferone | C10H8O3 | pos | 194.0816369 |
| Hovenoside D | C57H92O26 | pos | 597.2963547 |
| 5-Hydroxymethyl-4-methyluracil | C6H8N2O3 | pos | 157.061039 |
| Porfimer Sodium | C68H74N8O11 | pos | 601.2691642 |
| CDP-DG(PGJ2/i-19:0) | C51H85N3O17P2 | pos | 537.7710845 |
| 4-Ethylphenylsulfate | C8H10O4S | pos | 247.0016666 |
| Azimexon | C9H14N4O | pos | 239.0891228 |
| Patulin | C7H6O4 | pos | 187.0604614 |
| 2,5-Furandicarboxaldehyde | C6H4O3 | pos | 125.0236865 |
| Phenylbutyrylglutamine | C15H20N2O4 | pos | 315.1342628 |
| Methyltestosterone | C20H30O2 | pos | 303.2325571 |
| PS(18:1(11Z)/22:5(7Z,10Z,13Z,16Z,19Z)) | C46H78NO10P | pos | 858.5258537 |
| Anonaine | C17H15NO2 | pos | 569.1852156 |
| Suspensolide F | C21H34O12 | pos | 501.1977178 |
| Moexipril | C27H34N2O7 | pos | 481.233863 |
| 1-Arachidonoylglycerol | C23H38O4 | pos | 361.2743238 |
| PS(22:0/15:0) | C43H84NO10P | pos | 844.548036 |
| PC(20:2(11Z,14Z)/15:0) | C43H82NO8P | pos | 794.5719877 |
| PC(O-18:0/0:0) | C26H56NO6P | pos | 532.3748828 |
| 19(S)-HETE | C20H32O3 | pos | 343.2247957 |
| 5,6-Methylenedioxy-2-aminoindane | C10H11NO2 | pos | 219.1132746 |
| 8(R)-HETE | C20H32O3 | pos | 303.2320876 |
| PC(6:2(3E,5E)/14:2(11E,13E)) | C28H48NO8P | pos | 558.3197014 |
| N-Acetyl-L-glutamic acid | C7H11NO5 | pos | 231.0970793 |
| LysoPA(18:2(9Z,12Z)/0:0) | C21H39O7P | pos | 457.2335514 |
| PGB2 | C20H30O4 | pos | 335.2224435 |
| 3,4-dihydroxyphenylacetic Acid | C8H8O4 | pos | 169.0497943 |
| Estrone | C18H22O2 | pos | 315.134645 |
| 3,4-Dihydroxymandelic Acid | C8H8O5 | pos | 167.0341538 |
| 1-(5-Hydroxy-2-oxo-2,3-dihydroimidazol-4-yl)urea | C4H6N4O3 | pos | 222.0587614 |
| Epsilon-Caprolactone | C6H10O2 | pos | 229.1438951 |
| Phenyl-Alanine | C9H11NO2 | pos | 198.112917 |
| Sophoramine | C15H20N2O | pos | 227.154849 |
| CDP-DG(20:5(7Z,9Z,11E,13E,17Z)-3OH(5,6,15)/18:2(9Z,11Z)) | C50H79N3O18P2 | pos | 558.7308842 |
| PGP(22:5(4Z,7Z,10Z,13Z,16Z)/22:5(7Z,10Z,13Z,16Z,19Z)) | C50H80O13P2 | pos | 487.247219 |
| Corchoroside B | C29H42O8 | pos | 518.2836679 |
| Mepartricin | C60H88N2O19 | pos | 582.2959407 |
| 4-Imidazolone-5-propionic acid | C6H8N2O3 | pos | 157.061039 |
| Formiminoglutamic acid | C6H10N2O4 | pos | 157.0610554 |
| PI(LTE4/18:1(9Z)) | C50H86NO16PS | pos | 532.7613559 |
| 3-Hydroxymonoethylglycinexylidide | C12H18N2O2 | pos | 264.1712346 |
| N6-Acetyl-L-lysine | C8H16N2O3 | pos | 171.1132792 |
| S-(PGA1)-glutathione | C30H49N3O10S | pos | 676.3535292 |
| Lotaustralin | C11H19NO6 | pos | 244.1182779 |
| Oxypurinol | C5H4N4O2 | pos | 153.0408933 |
| Steviol | C20H30O3 | pos | 319.2272607 |
| LL-2,6-Diaminopimelic Acid | C7H14N2O4 | pos | 155.0816889 |
| Niacinamide | C6H6N2O | pos | 123.0556831 |
| (2,5-Dihydroxy-1H-pyrrol-3-yl) prop-2-enoate | C7H7NO4 | pos | 169.0358387 |
| Stachyose | C24H42O21 | pos | 689.213038 |
| L-Glutamic Acid | C5H9NO4 | pos | 148.0605495 |
| 1-Methylnicotinamide | C7H9N2O+ | pos | 137.0711024 |
| PC(18:0/20:4(5Z,8Z,11Z,14Z)-OH(19S)) | C46H84NO9P | pos | 848.5792899 |
| 1-(6Z,9Z,12Z-octadecatrienoyl)-glycero-3-phosphate | C21H37O7P | pos | 433.2335223 |
| PS(20:0/20:4(5Z,8Z,11Z,14Z)) | C46H82NO10P | pos | 822.5671468 |
| Valylglutamic acid | C10H18N2O5 | neg | 283.0688903 |
| 2-Methoxyacetaminophen sulfate | C9H11NO6S | neg | 242.0131467 |
| Methionyl-Histidine | C11H18N4O3S | neg | 321.0768077 |
| (3s)-3-(Benzyloxy)-L-Aspartic Acid | C11H13NO5 | neg | 260.0545254 |
| PIP(20:4(8Z,11Z,14Z,17Z)/18:1(9Z)) | C47H82O16P2 | neg | 945.4931507 |
| Mibefradil | C29H38FN3O3 | neg | 516.2686969 |
| CDP-DG(20:4(8Z,11Z,14Z,17Z)-2OH(5S,6R)/18:0) | C50H85N3O17P2 | neg | 529.7562972 |
| CDP-DG(PGJ2/20:2(11Z,14Z)) | C52H83N3O17P2 | neg | 540.7473562 |
| CTAP-III | C51H71N13O11S2 | neg | 551.7385216 |
| Vanilloyl glucose | C14H18O9 | neg | 351.0704057 |
| Pimonidazole | C11H18N4O3 | neg | 275.1143168 |
| Citrinin | C13H14O5 | neg | 249.0771846 |
| Albifylline | C13H20N4O3 | neg | 301.1298754 |
| 3-Butylidene-1(3H)-isobenzofuranone | C12H12O2 | neg | 233.0820764 |
| Ethyl maltol | C7H8O3 | neg | 185.0452612 |
| 3-Allylphenol sulfate | C9H10O4S | neg | 213.0226443 |
| 12,13-Epoxy-9-Octadecenoic Acid | C18H32O3 | neg | 295.2282361 |
| 9,10,13-TriHOME | C18H34O5 | neg | 329.233884 |
| 3a,6b,7a,12a-Tetrahydroxy-5b-cholanoic acid | C24H40O6 | neg | 405.2654412 |
| (11R,16S)-misoprostol | C22H38O5 | neg | 381.264838 |
| PA(i-14:0/20:3(6,8,11)-OH(5)) | C37H67O9P | neg | 685.4475114 |
| Allylestrenol | C21H32O | neg | 345.243859 |
| PE-NMe2(18:2(9Z,12Z)/20:3(8Z,11Z,14Z)) | C45H80NO8P | neg | 838.5633953 |
| Carboprost | C21H36O5 | neg | 389.2317364 |
| Isolithocholic acid | C24H40O3 | neg | 375.2909981 |
| PA(8:0/10:0) | C21H41O8P | neg | 473.2322027 |
| Prostaglandin G2 2-glyceryl Ester | C23H38O8 | neg | 477.2246492 |
| PI(6 keto-PGF1alpha/18:3(6Z,9Z,12Z)) | C47H79O17P | neg | 945.4935773 |
| PS(20:5(5Z,8Z,11Z,14Z,17Z)/PGJ2) | C46H70NO12P | neg | 858.4616058 |
| Octadecyl fumarate | C22H40O4 | neg | 349.2752411 |
| Manoalide | C25H36O5 | neg | 415.2474946 |
| 20, 22-Dihydrodigoxigenin | C23H36O5 | neg | 413.2319477 |
| Deoxycorticosterone acetate | C23H32O4 | neg | 353.2126888 |
| 3b,7a-Dihydroxy-5b-cholanoic acid | C24H40O4 | neg | 391.2861144 |
| Mevastatin | C23H34O5 | neg | 411.2160961 |
| Dihydrocortisol | C21H32O5 | neg | 385.2003321 |
| Prostaglandin A1 | C20H32O4 | neg | 335.2233333 |
| (+/-)-11,12-Dihydroxy-5Z,8Z,14Z,17Z-eicosatetraenoic acid | C20H32O4 | neg | 381.226723 |
| 9,10-Dihome | C18H34O4 | neg | 313.2389472 |
| 20-Hydroxy-leukotriene B4 | C20H32O5 | neg | 333.2075512 |
| (5Z)-7-[(1R,2E)-2-[(3S)-3-hydroxyoctylidene]-3-oxocyclopentyl]hept-5-enoylcarnitine | C27H45NO6 | neg | 516.274116 |
| Cromakalim | C16H18N2O3 | neg | 267.1143577 |
| Arg-Thr-Lys-Arg | C22H45N11O6 | neg | 580.3276621 |
| MG(16:1(9Z)/0:0/0:0) | C19H36O4 | neg | 309.244041 |
| (3S,5R,6R,7E)-3,5,6-Trihydroxy-7-megastigmen-9-one | C13H22O4 | neg | 287.1504715 |
| (+)-Lysergic acid | C16H16N2O2 | neg | 313.1196574 |
| Anisatin | C15H20O8 | neg | 349.0888641 |
| Alpha-Carboxy-delta-decalactone | C11H18O4 | neg | 259.1192572 |
| Pimelic Acid | C7H12O4 | neg | 141.0552335 |
| 3,5-Dichloro-2-hydroxybenzenesulfonic acid | C6H4Cl2O4S | neg | 240.9138229 |
| Nafazatrom | C16H16N2O2 | neg | 313.1201129 |
| 3-ethylphenyl Sulfate | C8H10O4S | neg | 201.0226062 |
| TETRAHYDROURIDINE | C9H16N2O6 | neg | 283.0688273 |
| Quillaic acid 3-[galactosyl-(1->2)-[rhamnosyl-(1->3)]-glucuronide] 28-[xylosyl-(1->4)-rhamnosyl-(1->2)-[rhamnosyl-(1->3)]-4acetyl-fucosyl] ester | C73H114O37 | neg | 790.3464939 |
| HBOA trihexose | C8H15NO2 | neg | 202.1083472 |
| 5,6-Dihydroxyprostaglandin F1a | C20H36O7 | neg | 387.2398972 |
| P-Cresol glucuronide | C13H16O7 | neg | 283.0828658 |
| CDP-DG(20:4(6Z,8E,10E,14Z)-2OH(5S,12R)/18:0) | C50H85N3O17P2 | neg | 529.7563177 |
| CDP-DG(20:2(11Z,14Z)/PGJ2) | C52H83N3O17P2 | neg | 540.7474079 |
| PS(22:6(4Z,7Z,10Z,13Z,16Z,19Z)/5-iso PGF2VI) | C46H72NO13P | neg | 858.4613055 |
| Marindinin | C14H16O3 | neg | 253.0835742 |
| Humilixanthin | C14H18N2O7 | neg | 307.091776 |
| Phenylacetylglycine | C10H11NO3 | neg | 192.0663869 |
| Xanthosine | C10H12N4O6 | neg | 283.069071 |
| Icariside B8 | C19H32O8 | neg | 387.203175 |
| Methylmalonic Acid | C4H6O4 | neg | 117.0188005 |
| Succinic Acid | C4H6O4 | neg | 117.0187919 |
| Glucomannan | C24H42O21 | neg | 701.1937772 |
| 12-KETE | C20H30O3 | neg | 317.2126248 |
| 13-HODE | C18H32O3 | neg | 295.2281361 |
| 10-HDoHE | C22H32O3 | neg | 343.2282719 |
| GPSer(2:0/18:3) | C26H44NO10P | neg | 560.2644092 |
| Prostaglandin E3 | C20H30O5 | neg | 331.1917542 |

Notes: Metabolite: name of the identified metabolite; Formula: chemical formula of the metabolite; Mode: ionic mode, pos positive, neg negative mode; M/Z: mass-to-charge ratio, the ratio of the mass of a charged ion to its charge.

**Supplementary Table 7. Callback fecal metabolites of EA**

| Metabolite | Formula | Mode | M/Z |
| --- | --- | --- | --- |
| LY 235959 | C11H20NO5P | pos | 300.095884 |
| PE(PGF1alpha/20:4(8Z,11Z,14Z,17Z)) | C45H78NO11P | pos | 862.519963 |
| Pseudouridine | C9H12N2O6 | pos | 245.0771178 |
| Levamisole | C11H12N2S | pos | 472.1591443 |
| Ribalinium | C16H20NO4+ | pos | 323.1718199 |
| Oleoyltaurine | C20H39NO4S | pos | 372.2600681 |
| 5,8-Dihydroxy-1,4-naphthoquinone | C10H6O4 | pos | 208.0607683 |
| 1-Benzylpiperidine-4-carboxylic acid | C13H17NO2 | pos | 202.1229318 |
| 20-Cooh ltb4 | C21H32O6 | pos | 425.1897629 |
| 1-[[(Z)-[5-(Hydroxymethyl)indol-3-ylidene]methyl]amino]-2-pentylguanidine | C16H23N5O | pos | 266.1755222 |
| Indole-3-Carboxaldehyde | C9H7NO | pos | 146.0602015 |
| 1,1,1-Trifluorohenicosa-6,9,12,15-tetraene | C21H33F3 | pos | 387.2254407 |
| Pyropheophorbide a | C33H34N4O3 | pos | 567.2977502 |
| 9H-Fluorene-9-carboxamide, 9-(3-aminopropyl)- | C17H18N2O | pos | 533.2863222 |
| Delta8,14-Sterol | C29H46O | pos | 411.362593 |
| Soyasapogenol C | C30H48O2 | pos | 504.3780471 |
| LysoPG(18:1(9Z)/0:0) | C24H47O9P | pos | 533.2812541 |
| Porphobilinogen | C10H14N2O4 | pos | 470.2263325 |
| Contignasterol | C29H48O7 | pos | 491.335969 |
| Alpha-Dimorphecolic acid | C18H32O3 | pos | 261.2214698 |
| 7alpha,24-dihydroxycholest-4-en-3-one | C27H44O3 | pos | 439.3177213 |
| Val-Phe-Val-Tyr | C28H38N4O6 | pos | 559.3105146 |
| Byakangelicol | C17H16O6 | pos | 334.1294703 |
| FLAVOKAWAIN B | C17H16O4 | pos | 285.112568 |
| Hesperetin | C16H14O6 | pos | 320.1134518 |
| Gamma-Glutamylglutamic acid | C10H16N2O7 | pos | 299.0854016 |
| N-Hydroxy-L-tyrosine | C9H11NO4 | pos | 162.055096 |
| Netilmicin | C21H41N5O7 | pos | 476.3077643 |
| (-)-Usnic acid | C18H16O7 | pos | 345.0974116 |
| Noreugenin | C10H8O4 | pos | 210.0763706 |
| Cortisol | C21H30O5 | pos | 363.2174435 |
| Dehydrosafynol | C13H10O2 | pos | 221.0560156 |
| Panthenol | C9H19NO4 | pos | 206.1390437 |
| Nivalenol | C15H20O7 | pos | 354.1529955 |
| Mefloquine | C17H16F6N2O | pos | 379.1219171 |
| ARGININOSUCCINATE | C10H18N4O6 | pos | 335.095444 |
| 4-Methyl-5-Thiazoleethanol | C6H9NOS | pos | 144.047922 |
| Thiamine | C12H17N4OS+ | pos | 265.1119775 |
| Coumafuryl | C17H14O5 | neg | 343.0828189 |
| Gossyvertin | C16H18O4 | neg | 273.1138007 |
| S-sulfanylglutathione | C10H16N3O6S2- | neg | 337.0395475 |
| Fasoracetam | C10H16N2O2 | neg | 241.1194856 |
| TWS119 | C18H14N4O2 | neg | 317.1040611 |
| Salicylic Acid | C7H6O3 | neg | 137.0239029 |
| Cajanol | C17H16O6 | neg | 361.0937167 |
| (E)-8-Hydroxy-2-octene-4,6-diynoic acid | C8H6O3 | neg | 359.0784069 |
| Theobromine | C7H8N4O2 | neg | 161.0479579 |
| P-Toluenesulfonic Acid | C7H8O3S | neg | 171.0117121 |
| 3-(3-Oxobutanoyloxy)butanoic acid | C8H12O5 | neg | 223.0377266 |
| TRIAZIQUONE | C12H13N3O2 | neg | 276.0993761 |
| 10-Formyldihydrofolate | C20H21N7O7 | neg | 470.1442292 |
| 5-Hydroxy-N-formylkynurenine | C11H12N2O5 | neg | 251.06843 |
| Glucose pyruvate | C9H14O9 | neg | 265.0569666 |
| Gentiobioside | C13H24O11 | neg | 391.1023644 |
| 9,10-Epoxyoctadecanoic acid | C18H34O4 | neg | 313.2388469 |
| Durandro | C27H40O3 | neg | 457.2957433 |
| Ile-Ile-Ile-Pro | C23H42N4O5 | neg | 491.2643848 |
| S-(6-Purinyl)cysteine | C8H9N5O2S | neg | 275.9942466 |
| 3,4-dihydroxyphenylacetic Acid | C8H8O4 | neg | 167.0346458 |
| Salicyluric acid | C9H9NO4 | neg | 176.0349032 |
| Toluene diisocyanate | C9H6N2O2 | neg | 219.0410614 |
| 3-Hydroxyoctanoic acid | C8H16O3 | neg | 319.2131339 |
| Neotussilagine | C10H17NO3 | neg | 244.1192241 |
| 3-Piperidinecarboxylic acid, 1-(4,4-bis(3-methyl-2-thienyl)-3-butenyl)-, (S)- | C20H25NO2S2 | neg | 374.128084 |
| 7-Sulfocholic acid | C24H40O8S | neg | 487.2382725 |
| Floionolic acid | C18H36O5 | neg | 331.2492954 |
| 5-Hydroxylysine | C6H14N2O3 | neg | 485.2919159 |
| PE(12:0/0:0) | C17H36NO7P | neg | 396.2159375 |
| PA(12:0/18:1(12Z)-2OH(9,10)) | C33H63O10P | neg | 685.3826558 |
| SM(d19:0/PGD2) | C44H83N2O9P | neg | 849.5529723 |
| Hex-3-enedioylcarnitine | C13H21NO6 | neg | 322.1051068 |
| 3a,7a-Dihydroxy-5b-cholestane | C27H48O2 | neg | 449.3646221 |
| Nuatigenin | C27H42O4 | neg | 429.3016814 |
| PA(18:1(12Z)-2OH(9,10)/a-13:0) | C34H65O10P | neg | 699.3982087 |
| N-Oleoyl Cysteine | C21H39NO3S | neg | 769.5286822 |
| Cholestane-3,7,12,25-tetrol-3-glucuronide | C33H56O10 | neg | 593.3708115 |
| N-Palmitoyl Methionine | C21H41NO3S | neg | 408.2527762 |
| Arginyl-glycyl-aspartic acid | C12H22N6O6 | neg | 345.1498627 |
| N-Lauroylglycine | C14H27NO3 | neg | 256.1919993 |
| (17alpha,23S)-Epoxy-28,29-dihydroxy-27-norlanost-8-ene-3,24-dione | C29H44O5 | neg | 517.3175445 |
| Euscaphic acid | C30H48O5 | neg | 487.3435662 |
| Cuminaldehyde | C10H12O | neg | 193.0865902 |
| Ganodosterone | C28H40O2 | neg | 875.6269208 |
| Cyasterone | C29H44O8 | neg | 501.2868759 |
| 3-hydroxypristanic acid | C19H38O3 | neg | 359.2807838 |
| (11S,12S,13S)-Epoxy-hydroxyoctadeca-cis-9-cis-15-dien-1-oic acid | C18H30O4 | neg | 331.1920731 |
| 3,4-Dihydroxymandelic Acid | C8H8O5 | neg | 205.0116473 |
| 9,10,16-trihydroxy palmitic acid | C16H32O5 | neg | 303.2177835 |
| Subaphylline | C14H20N2O3 | neg | 573.293188 |
| Pantethine | C22H42N4O8S2 | neg | 553.2389378 |
| N-Lauroyl Lysine | C18H36N2O3 | neg | 363.2395408 |
| 11b-Hydroxyprogesterone | C20H28O4 | neg | 377.197761 |
| 3-[[5-Methyl-2-(1-methylethyl)cyclohexyl]oxy]-1,2-propanediol | C13H26O3 | neg | 275.1867617 |
| 4,4'-Sulfonyldiphenol | C12H10O4S | neg | 249.0229369 |
| MG(0:0/20:4(5Z,8Z,11Z,14Z)/0:0) | C23H38O4 | neg | 423.2763853 |
| N,N'-Di-1,2,3,4-Tetrahydroacridin-9-Ylheptane-1,7-Diamine | C33H40N4 | pos | 510.3564288 |
| 1-Hydroxypropan-2-yl 2-isopropyl-5-methylcyclohexyl carbonate | C14H26O4 | pos | 281.172494 |
| Undecylenic acid | C11H20O2 | pos | 185.1537 |
| Benzotest | C26H38O3 | pos | 381.2793864 |
| Lacto-N-biose I | C14H25NO11 | pos | 366.1403479 |
| 18-Nor-4(19),8,11,13-abietatetraene | C19H26 | pos | 255.2109736 |
| Dihydroceramide | C19H39NO3 | pos | 330.3006232 |
| Ovalicin | C16H24O5 | pos | 593.3334891 |
| 5-NITRO-2-PHENYLPROPYLAMINOBENZOIC ACID [NPPB] | C16H16N2O4 | pos | 301.1184075 |
| Cilengitide | C27H40N8O7 | pos | 589.3037542 |
| Avadomide | C14H14N4O3 | pos | 287.1143086 |
| Ethenoadenosine | C12H13N5O4 | pos | 274.0926619 |
| Gly Ile Arg | C14H28N6O4 | pos | 345.2252754 |
| Phe Val Lys | C20H32N4O4 | pos | 197.1287758 |
| 7-Hydroxy-8-O-methylaloin B | C22H24O10 | pos | 471.1277318 |
| Linalyl propionate | C13H22O2 | pos | 175.1483306 |
| Lupinic acid | C10H17NO2 | pos | 389.2401511 |
| Gly Ile Lys | C14H28N4O4 | pos | 317.2189169 |
| Ala Val Val | C13H25N3O4 | pos | 288.1919762 |
| Valganciclovir, (S)- | C14H22N6O5 | pos | 319.1520722 |
| DG(20:4(7E,9E,11Z,13E)-3OH(5S,6R,15S)/0:0/8:0) | C31H52O8 | pos | 288.179845 |
| Gly Ile Val | C13H25N3O4 | pos | 288.1921203 |
| Kinetensin 4-7 | C26H37N9O6 | pos | 613.3251599 |
| LysoPE(16:1(9Z)/0:0) | C21H42NO7P | pos | 452.2780462 |
| CDP-DG(22:6(4Z,7Z,10Z,12E,16Z,19Z)-OH(14)/20:3(8Z,11Z,14Z)) | C54H83N3O16P2 | pos | 546.7714369 |
| Piperdial | C15H22O3 | pos | 289.1187255 |
| Ala Tyr Ile | C18H27N3O5 | pos | 366.2027176 |
| 6-Keto-prostaglandin F1a | C20H34O6 | pos | 370.2346065 |
| PG(20:3(8Z,11Z,14Z)/PGF1alpha) | C46H81O13P | pos | 459.2594099 |
| 7-Hydroxy-6-(methoxyacetyl)-2,2-dimethyl-2H-1-benzopyran | C14H16O4 | pos | 249.1123427 |
| Ile Glu Leu | C17H31N3O6 | pos | 374.2292905 |
| Cervonoyl ethanolamide | C24H36O3 | pos | 355.2635075 |
| 2,4,5,7alpha-Tetrahydro-1,4,4,7a-tetramethyl-1H-inden-2-ol | C13H20O | pos | 175.1482782 |
| 17-Hydroxy-3,11,20-trioxopregn-4-en-21-yl acetate | C23H30O6 | pos | 402.2007822 |
| 5'-Deoxy-5-fluorocytidine | C9H12FN3O4 | pos | 287.1141905 |
| Oxyacanthine | C37H40N2O6 | pos | 626.3172845 |
| Edulitine | C11H11NO3 | pos | 170.0601222 |
| DG(14:1(9Z)/16:1(9Z)/0:0) | C33H60O5 | pos | 536.4437614 |
| Melanettin | C16H12O5 | pos | 302.1027912 |
| Dicyclohexylamine | C12H23N | pos | 226.1551514 |
| 1-{2-[(3-Ethylphenyl)amino]-2-oxoethyl}-6-oxo-1,6-dihydropyridine-3-carboxylic acid | C16H16N2O4 | pos | 301.1186407 |
| (5Z,7S,8E,10Z,13Z,15E,17S,19Z)-7,17-Dihydroxydocosa-5,8,10,13,15,19-hexaenoylcarnitine | C29H45NO6 | pos | 542.2907794 |
| Fluvoxamino acid | C14H17F3N2O3 | pos | 301.1187163 |
| Tragopogonsaponin J | C57H84O22 | pos | 583.2566936 |
| N-Linoleoyl Histidine | C24H39N3O3 | pos | 462.268473 |
| Sulfolithocholylglycine | C26H43NO7S | pos | 531.3115993 |
| 7-a,27-Dihydroxycholesterol | C27H46O3 | pos | 401.3420682 |
| Alpha-Muricholic Acid | C24H40O5 | pos | 426.3223852 |
| Phenacyl 5-[(3aR,4S,5R,6aR)-5-hydroxy-4-[(3R,4S)-3-hydroxy-4-methyloct-1-en-6-ynyl]-3,3a,4,5,6,6a-hexahydro-1H-pentalen-2-ylidene]pentanoate | C30H38O5 | pos | 461.273246 |
| Benzoylmesaconine | C31H43NO10 | pos | 628.2517436 |
| Endomorphin-2 | C32H37N5O5 | pos | 610.2409471 |
| DG(2:0/0:0/PGD1) | C25H42O8 | pos | 471.2935038 |
| Adrenic acid | C22H36O2 | pos | 355.2635821 |
| Nandrolone | C18H26O2 | pos | 239.1793998 |
| Fumagillol | C16H26O4 | pos | 247.1693437 |
| (1R,7S,13S,15S)-2,15-Dihydroxy-7-methyl-6-oxabicyclo[11.3.0]hexadeca-3,11-dien-5-one | C16H24O4 | pos | 245.1537145 |
| (4E,7E,10E,13E)-Hexadeca-4,7,10,13-tetraenoic acid | C16H24O2 | pos | 213.1638687 |
| 7-Ketodeoxycholic acid | C24H38O5 | pos | 424.3065874 |
| Nonadecanoic acid | C19H38O2 | pos | 316.3212835 |
| Andrastin D | C26H36O5 | pos | 470.2891558 |
| PC(20:2(11Z,14Z)/TXB2) | C48H86NO12P | pos | 922.5723274 |
| 1-Phenyl-1-cyclohexene | C12H14 | pos | 317.226421 |
| 4-oxo-Retinoic acid | C20H26O3 | pos | 297.1850898 |
| Estriol | C18H24O3 | pos | 271.169378 |
| 4-Hydroxyestrone-2-S-glutathione | C28H37N3O9S | pos | 633.2570703 |
| N-Eicosapentaenoyl Arginine | C26H42N4O3 | pos | 500.3594512 |
| (2xi,4xi)-2,4-Nonadien-1-ol | C9H16O | pos | 158.1540954 |
| Deoxyspergualin | C17H37N7O3 | pos | 370.2957044 |
| Violet-leaf aldehyde | C9H14O | pos | 156.1384665 |
| Estrone | C18H22O2 | pos | 315.134187 |
| DG(8:0/i-19:0/0:0) | C30H58O5 | pos | 537.3912696 |
| Citpressine II | C17H17NO5 | pos | 333.1446927 |
| Metoprolol | C15H25NO3 | pos | 535.3756039 |
| DG(8:0/19:0/0:0) | C30H58O5 | pos | 537.3914475 |
| DG(16:1(9Z)/14:1(9Z)/0:0) | C33H60O5 | pos | 536.4437862 |
| (2,5-Dihydroxypyrrol-1-yl) 4-[(2,5-dioxopyrrol-1-yl)methyl]cyclohexane-1-carboxylate | C16H18N2O6 | pos | 379.0852452 |
| N-Acetylneuraminic Acid | C11H19NO9 | pos | 310.113847 |
| Herniarin | C10H8O3 | pos | 177.0547438 |
| Aflatoxin M1 | C17H12O7 | pos | 367.0225574 |
| Urobilinogen | C33H42N4O6 | pos | 613.301268 |
| W Hydroxy testosterone | C19H28O3 | pos | 343.1653141 |
| 5-p-Coumaroylquinic acid | C16H18O8 | pos | 339.1082304 |
| Dolichyl b-D-glucosyl phosphate | C21H39O9P | pos | 466.2347361 |
| (3-Aminopropyl)(n-butyl)phosphinic acid | C7H18NO2P | pos | 144.0935289 |
| Questiomycin A | C12H8N2O2 | pos | 245.0922543 |
| (4As,5aR,6R,12aR)-1,10,11,12a-tetrahydroxy-6-methyl-3,12-dioxo-4a,5,5a,6-tetrahydro-4H-tetracene-2-carboxamide | C20H19NO7 | pos | 386.12438 |
| Prazepam | C19H17ClN2O | pos | 342.134296 |
| Angiotensin II | C50H71N13O12 | pos | 534.7607018 |
| Thr Leu Leu | C16H31N3O5 | pos | 346.234284 |
| Noopept | C17H22N2O4 | pos | 360.1931295 |
| 5-Hydroxydecanedioylcarnitine | C17H31NO7 | pos | 326.1970462 |
| N-Eicosapentaenoyl Phenylalanine | C29H39NO3 | pos | 488.2551066 |
| Monoethylglycinexylidide | C12H18N2O | pos | 451.2481671 |
| EPIMESTROL | C19H26O3 | pos | 347.1606822 |
| Sulfoglycolithocholate(2-) | C26H43NO7S | pos | 496.2737493 |
| Glucosylgalactosyl hydroxylysine | C18H34N2O13 | pos | 504.2388041 |
| Gly Leu Tyr | C17H25N3O5 | pos | 352.1872109 |
| Ser Val Leu | C14H27N3O5 | pos | 318.2027397 |
| Asn Val Leu | C15H28N4O5 | pos | 345.2140922 |
| (4R,6S)-p-Menth-1-ene-4,6-diol 4-glucoside | C16H28O7 | pos | 374.2181037 |
| Taurocholic acid | C26H45NO7S | pos | 480.2790308 |
| Asn Ile Val | C15H28N4O5 | pos | 345.2141512 |
| Fumagillin | C26H34O7 | pos | 423.216964 |
| Ubiquinone-1 | C14H18O4 | pos | 501.2465014 |
| Taurodeoxycholic acid | C26H45NO6S | pos | 464.2846675 |
| PS(TXB2/18:2(9Z,12Z)) | C44H76NO14P | pos | 459.7389568 |
| 5'-Methylthioadenosine | C11H15N5O3S | pos | 298.0972928 |
| Phe Ile Lys | C21H34N4O4 | pos | 407.2662171 |
| Kanamycin | C18H36N4O11 | pos | 449.225437 |
| Ile Leu Lys | C18H36N4O4 | pos | 373.2817343 |
| Ala Ile Arg | C15H30N6O4 | pos | 359.2407337 |
| Asp Ile Lys | C16H30N4O6 | pos | 375.224314 |
| Ile Ala Lys | C15H30N4O4 | pos | 331.2345442 |
| Ala Leu Lys | C15H30N4O4 | pos | 331.2345074 |
| Ala Leu Phe | C18H27N3O4 | pos | 350.2080847 |
| METHACHOLINE | C8H17NO2 | pos | 160.1333775 |
| L-Aspartic Acid | C4H7NO4 | pos | 134.0449394 |
| Sulfonamidoethanol | C2H7NO3S | pos | 126.0222527 |
| 2,7-dihydroxy-4'-methoxyisoflavanone | C16H14O5 | pos | 287.091941 |
| Cortisol lactate | C24H34O8 | neg | 431.2116839 |
| Cyanidin 3-O-alpha-L-arabinoside | C20H19O10+ | neg | 400.0834516 |
| Riboprine | C15H21N5O4 | neg | 372.1095078 |
| P-Tolyl Sulfate | C7H8O4S | neg | 187.0066985 |
| 5-(5-Heptyl-3-methylfuran-2-yl)pentanoylcarnitine | C24H41NO5 | neg | 460.2498298 |
| 3-Hydroxycarbamazepine | C15H12N2O2 | neg | 289.0393305 |
| O-methoxycatechol-O-sulphate | C7H8O5S | neg | 203.0017215 |
| Gibberellin A37 | C20H26O5 | neg | 345.1727241 |
| Glycerophosphoinositol | C9H19O11P | neg | 333.0622952 |
| Dihydrowogonin 7-glucoside | C22H24O10 | neg | 447.1309116 |
| Deoxyloganin | C17H26O9 | neg | 373.1511969 |
| Cis-Ferulic acid 4-sulfate | C10H10O7S | neg | 273.0080299 |
| 3-Methoxy-4-hydroxyphenylglycol glucuronide | C15H20O10 | neg | 341.0883764 |
| Dihydrokaempferol | C15H12O6 | neg | 287.0567251 |
| 3-hydroxy-3-(3-hydroxyphenyl)propanoic acid-O-sulphate | C9H10O7S | neg | 261.0077788 |
| 4-Ethylphenylsulfate | C8H10O4S | neg | 247.0284451 |
| Phenol sulphate | C6H6O4S | neg | 172.9910371 |
| 4-Morpholinepropanesulfonic acid | C7H15NO4S | neg | 208.0648308 |
| Mono-(2-ethyl-5-hydroxyhexyl) phthalate | C16H22O5 | neg | 587.2892161 |
| 3-(3,5-dihydroxyphenyl)-1-propanoic acid sulphate | C9H10O7S | neg | 261.0079024 |
| 2,5-Dihydroxybenzenesulfonic Acid | C6H6O5S | neg | 188.9860179 |
| 6-Hydroxynorketamine | C12H14ClNO2 | neg | 274.039656 |
| Esmolol | C16H25NO4 | neg | 316.1518129 |
| Aminocatechol N-acetate sulfate | C8H9NO6S | neg | 227.9972751 |
| 7-Methylguanosine 5'-phosphate | C11H17N5O8P+ | neg | 377.0707954 |
| Cyclopropanecarboxamide, 1,2,2,3,3-pentamethyl- | C9H17NO | neg | 200.1289692 |
| (2R,4S)-4-Carbamimidamido-3-acetamido-2-((1R,2R)-2-hydroxy-1-methoxy-3-(octanoyloxy)propyl)-3,4-dihydro-2H-pyran-6-carboxylic acid | C21H36N4O8 | neg | 471.2429239 |
| Allocholic acid | C24H40O5 | neg | 407.280894 |
| Amastatin | C21H38N4O8 | neg | 473.2593038 |
| Apiole | C12H14O4 | neg | 443.1750053 |
| D-Glucuronic acid 1-phosphate | C6H11O10P | neg | 273.0003983 |
| Taurine | C2H7NO3S | neg | 124.0068753 |
| Methionine sulfone | C5H11NO4S | neg | 180.0332791 |
| (1S,2S,3S,4R)-3-(1-Acetamido-2-ethylbutyl)-4-(diaminomethylideneamino)-2-hydroxycyclopentane-1-carboxylic acid | C15H28N4O4 | neg | 373.2097484 |
| Norketamine | C12H14ClNO | neg | 260.0238786 |
| Indole carboxylic acid sulfate | C9H7NO5S | neg | 239.9973463 |
| 3-ureido-isobutyrate | C5H10N2O3 | neg | 291.1282138 |
| 7,8-Dichloro-1,2,3,4-tetrahydroisoquinoline | C9H9Cl2N | neg | 246.0080153 |
| 2-Hydroxy-4-methoxyacetophenone 5-sulfate | C9H10O7S | neg | 261.0078591 |
| Lersivirine | C17H18N4O2 | neg | 291.1277234 |
| Acridonecarboxamide | C14H10N2O2 | neg | 275.023603 |
| Cis-Melilotoside | C15H18O8 | neg | 325.0937842 |
| 3-Methoxy-4-Hydroxyphenylglycol sulfate | C9H12O7S | neg | 245.0126756 |
| 2-Methoxyacetaminophen sulfate | C9H11NO6S | neg | 242.013053 |
| LysoPC(14:1(9Z)/0:0) | C22H44NO7P | neg | 486.2580132 |
| 2-Propenamide, 2-cyano-3-(4-hydroxy-3,5-bis(1-methylethyl)phenyl)- | C16H20N2O2 | neg | 307.1224275 |
| (E)-4,4'-(Hex-3-ene-3,4-diyl)bis(4,1-phenylene) bis(dihydrogen phosphate) | C18H22O8P2 | neg | 409.0606908 |
| Methylthiomethyl hexanoate | C8H16O2S | neg | 221.0853476 |
| N-[4-Cloro-3-(T-butyloxome)phenyl-2-methyl-3-furan-carbothiamide | C17H19ClN2O2S | neg | 395.0813295 |
| Pioglitazone | C19H20N2O3S | neg | 393.0659406 |
| Phospho-ibuprofen | C13H17O4P | neg | 303.0548193 |
| 5'-Phosphoribosyl-N-formylglycinamide | C8H15N2O9P | neg | 335.0236979 |
| [(S)-1-Carboxy-2-phenyl-ethyl]-carbamoyl-Arg-Val-Arg-aldehyde | C27H44N10O6 | neg | 625.3225471 |
| 3-Methylpimelic acid | C8H14O4 | neg | 155.0708593 |
| Glycosminine | C15H12N2O | neg | 273.0443476 |
| Phenyl salicylate | C13H10O3 | neg | 259.0615443 |
| 6b-Hydroxy-8a-methoxy-7(11)-eremophilen-12,8-olide | C16H24O4 | neg | 317.1149816 |
| Hydroxypioglitazone | C19H20N2O4S | neg | 407.0814637 |
| 3-Hydroxyhalazepam | C17H12ClF3N2O2 | neg | 389.0319338 |
| 3-ethylphenyl Sulfate | C8H10O4S | neg | 201.0223901 |
| Austin | C27H32O9 | neg | 499.2016325 |
| Cambendazole | C14H14N4O2S | neg | 323.0597817 |
| 9-(3-Imidazol-1-yl-2,6,6-trimethylcyclohexen-1-yl)-3,7-dimethylnona-2,4,6,8-tetraenoic acid | C23H30N2O2 | neg | 403.1802338 |
| Isoaustin | C27H32O9 | neg | 499.2011175 |
| Benzamide, N-[2-[4-(cyclopropylcarbonyl)-3-methyl-1-piperazinyl]-1-[[7-(1,1-dimethylethyl)-1H-indol-3-yl]methyl]-2-oxoethyl]-4-nitro- | C31H37N5O5 | neg | 580.2575532 |
| 3a,6b,7a,12a-Tetrahydroxy-5b-cholanoic acid | C24H40O6 | neg | 405.2653816 |
| Ketamine | C13H16ClNO | neg | 274.0396278 |
| Prednisolone hemisuccinate | C25H32O8 | neg | 481.1889121 |
| Sambacin | C26H36O12 | neg | 575.1923802 |
| Atorvastatin lactone | C33H33FN2O4 | neg | 561.2131666 |
| 7-O-Succinyl macrolactin A | C28H38O8 | neg | 483.2421726 |
| Taurochenodeoxycholate-7-sulfate | C26H45NO9S2 | neg | 288.6199962 |
| Cholic Acid | C24H40O5 | neg | 815.5706096 |
| 1b,3a,7b-Trihydroxy-5b-cholanoic acid | C24H40O6 | neg | 405.2656086 |
| Gibberellin A38 glucosyl ester | C26H36O11 | neg | 559.1972432 |
| 4-Hydroxy-2,6,6-trimethyl-3-oxo-1,4-cyclohexadiene-1-carboxaldehyde | C10H12O3 | neg | 539.2311709 |
| PC(18:1(11Z)/18:3(9Z,12Z,15Z)) | C44H80NO8P | neg | 762.5372927 |
| Pinometostat | C30H42N8O3 | neg | 599.288913 |
| Dihydrobiopterin | C9H13N5O3 | neg | 537.2151772 |
| Bazedoxifene | C30H34N2O3 | neg | 507.2043104 |
| Cinncassiol C1 19-glucoside | C26H38O12 | neg | 577.2080358 |
| [(3R,4S)-1,1-Difluoro-3-(hexadecanoylamino)-4-hydroxy-4-phenylbutyl]phosphonic acid | C26H44F2NO5P | neg | 500.2695543 |
| 3-Dehydrocholic Acid | C24H38O5 | neg | 811.5392314 |
| 3-Hydroxybenzyl alcohol glucoside | C13H18O7 | neg | 285.0997999 |
| Amobarbital | C11H18N2O3 | neg | 225.1243886 |
| Cinobufagin | C26H34O6 | neg | 487.2373862 |
| Ceanothine C | C26H38N4O4 | neg | 515.2891698 |
| Dodecyl-beta-D-maltoside | C24H46O11 | neg | 555.3004831 |
| Tempo | C9H19NO | neg | 156.1388659 |
| (-)-Naringenin | C15H12O5 | neg | 271.0615629 |
| N-Palmitoyl Aspartic acid | C26H43NO10S2 | neg | 295.6094291 |
| Gamma-Glutamyl-S-(1-propenyl)cysteine sulfoxide | C11H18N2O6S | neg | 351.08576 |
| Fenleuton | C17H15FN2O3 | neg | 351.0547571 |
| (S)-Equol | C15H14O3 | neg | 241.0875293 |
| Urobilin | C33H42N4O6 | neg | 589.3046824 |
| Pantetheine 4'-phosphate | C11H23N2O7PS | neg | 393.0657539 |
| Avatrombopag | C29H34Cl2N6O3S2 | neg | 323.0678651 |
| Vulgarin | C15H20O4 | neg | 587.2892042 |
| N-Acetyl-leukotriene E4 | C25H39NO6S | neg | 526.249292 |
| Hallacridone | C18H13NO4 | neg | 288.066426 |
| N-[(3a,5b,7a)-3-hydroxy-24-oxo-7-(sulfooxy)cholan-24-yl]-Glycine | C26H43NO8S | neg | 528.2647723 |
| Padmatin | C16H14O7 | neg | 317.0670481 |
| Pglu-his-pro | C16H21N5O5 | neg | 398.1252697 |
| Sakuranin | C22H24O10 | neg | 493.1366232 |

Notes: Metabolite: name of the identified metabolite; Formula: chemical formula of the metabolite; Mode: ionic mode, pos positive, neg negative mode; M/Z: mass-to-charge ratio, the ratio of the mass of a charged ion to its charge.

**Supplementary Table 8. Callback fecal metabolites of MA**

| Metabolite | Formula | Mode | M/Z |
| --- | --- | --- | --- |
| N,N'-Di-1,2,3,4-Tetrahydroacridin-9-Ylheptane-1,7-Diamine | C33H40N4 | pos | 510.3564288 |
| Benzotest | C26H38O3 | pos | 381.2793864 |
| Dihydroceramide | C19H39NO3 | pos | 330.3006232 |
| 5-NITRO-2-PHENYLPROPYLAMINOBENZOIC ACID [NPPB] | C16H16N2O4 | pos | 301.1184075 |
| Cilengitide | C27H40N8O7 | pos | 589.3037542 |
| Avadomide | C14H14N4O3 | pos | 287.1143086 |
| Ethenoadenosine | C12H13N5O4 | pos | 274.0926619 |
| Indole-3-acetaldehyde | C10H9NO | pos | 177.1024837 |
| 7-Hydroxy-8-O-methylaloin B | C22H24O10 | pos | 471.1277318 |
| Linalyl propionate | C13H22O2 | pos | 175.1483306 |
| 2,5,8-trimethylquinolin-4-ol | C12H13NO | pos | 188.1072643 |
| 2-Methylbenzaldehyde | C8H8O | pos | 121.0652539 |
| 4-Methylbenzaldehyde | C8H8O | pos | 121.0652044 |
| Fluoroacetic acid | C2H3FO2 | pos | 174.0586434 |
| Galactaric acid | C6H10O8 | pos | 252.0729743 |
| N-Monodemethylolopatadine | C20H21NO3 | pos | 368.1209152 |
| Valganciclovir, (S)- | C14H22N6O5 | pos | 319.1520722 |
| Octanedioylcarnitine | C15H27NO6 | pos | 318.1916201 |
| Kinetensin 4-7 | C26H37N9O6 | pos | 613.3251599 |
| Piperdial | C15H22O3 | pos | 289.1187255 |
| 6-Keto-prostaglandin F1a | C20H34O6 | pos | 370.2346065 |
| 7-Hydroxy-6-(methoxyacetyl)-2,2-dimethyl-2H-1-benzopyran | C14H16O4 | pos | 249.1123427 |
| Cervonoyl ethanolamide | C24H36O3 | pos | 355.2635075 |
| 5'-Deoxy-5-fluorocytidine | C9H12FN3O4 | pos | 287.1141905 |
| DG(14:1(9Z)/16:1(9Z)/0:0) | C33H60O5 | pos | 536.4437614 |
| Melanettin | C16H12O5 | pos | 302.1027912 |
| S-Hydroxymethylglutathione | C11H19N3O7S | pos | 355.1274036 |
| 1-{2-[(3-Ethylphenyl)amino]-2-oxoethyl}-6-oxo-1,6-dihydropyridine-3-carboxylic acid | C16H16N2O4 | pos | 301.1186407 |
| (5Z,7S,8E,10Z,13Z,15E,17S,19Z)-7,17-Dihydroxydocosa-5,8,10,13,15,19-hexaenoylcarnitine | C29H45NO6 | pos | 542.2907794 |
| Fluvoxamino acid | C14H17F3N2O3 | pos | 301.1187163 |
| Tragopogonsaponin J | C57H84O22 | pos | 583.2566936 |
| Tridemorph | C19H39NO | pos | 298.3107097 |
| 10,20-Dihydroxyeicosanoic acid | C20H40O4 | pos | 327.2895608 |
| 7-a,27-Dihydroxycholesterol | C27H46O3 | pos | 401.3420682 |
| Phenacyl 5-[(3aR,4S,5R,6aR)-5-hydroxy-4-[(3R,4S)-3-hydroxy-4-methyloct-1-en-6-ynyl]-3,3a,4,5,6,6a-hexahydro-1H-pentalen-2-ylidene]pentanoate | C30H38O5 | pos | 461.273246 |
| LysoPE(0:0/15:0) | C20H42NO7P | pos | 440.2781438 |
| 2-Naphthylamine | C10H9N | pos | 144.080867 |
| Metapro | C22H36N2O6 | pos | 488.2750086 |
| Methyl linoleate | C19H34O2 | pos | 295.2633206 |
| 7alpha-Hydroxy-3-oxo-4-cholestenoate | C27H42O4 | pos | 431.3122518 |
| DG(2:0/0:0/PGD1) | C25H42O8 | pos | 471.2935038 |
| Hydrocotarnine | C12H15NO3 | pos | 460.2440293 |
| Nonadecanoic acid | C19H38O2 | pos | 316.3212835 |
| PC(20:2(11Z,14Z)/TXB2) | C48H86NO12P | pos | 922.5723274 |
| Lilopristone | C29H37NO3 | pos | 480.3150008 |
| 4-Hydroxyestrone-2-S-glutathione | C28H37N3O9S | pos | 633.2570703 |
| N-Eicosapentaenoyl Arginine | C26H42N4O3 | pos | 500.3594512 |
| (2xi,4xi)-2,4-Nonadien-1-ol | C9H16O | pos | 158.1540954 |
| Deoxyspergualin | C17H37N7O3 | pos | 370.2957044 |
| 19-Hydroxyandrost-4-ene-3,17-dione | C19H26O3 | pos | 347.1605133 |
| Estrone | C18H22O2 | pos | 315.134187 |
| DG(8:0/i-19:0/0:0) | C30H58O5 | pos | 537.3912696 |
| Citpressine II | C17H17NO5 | pos | 333.1446927 |
| Dehydrocarpaine I | C28H48N2O4 | pos | 477.3700514 |
| Metoprolol | C15H25NO3 | pos | 535.3756039 |
| DG(8:0/19:0/0:0) | C30H58O5 | pos | 537.3914475 |
| DG(16:1(9Z)/14:1(9Z)/0:0) | C33H60O5 | pos | 536.4437862 |
| (2,5-Dihydroxypyrrol-1-yl) 4-[(2,5-dioxopyrrol-1-yl)methyl]cyclohexane-1-carboxylate | C16H18N2O6 | pos | 379.0852452 |
| N-Acetylneuraminic Acid | C11H19NO9 | pos | 310.113847 |
| Urobilinogen | C33H42N4O6 | pos | 613.301268 |
| 4-Chloro-2-nitrobenzylalcohol | C29H37N3O6 | pos | 587.287804 |
| W Hydroxy testosterone | C19H28O3 | pos | 343.1653141 |
| 5-p-Coumaroylquinic acid | C16H18O8 | pos | 339.1082304 |
| Perampanel | C23H15N3O | pos | 382.155138 |
| Dolichyl b-D-glucosyl phosphate | C21H39O9P | pos | 466.2347361 |
| (3-Aminopropyl)(n-butyl)phosphinic acid | C7H18NO2P | pos | 144.0935289 |
| (4As,5aR,6R,12aR)-1,10,11,12a-tetrahydroxy-6-methyl-3,12-dioxo-4a,5,5a,6-tetrahydro-4H-tetracene-2-carboxamide | C20H19NO7 | pos | 386.12438 |
| Isoeugenol | C10H12O2 | pos | 197.117555 |
| Prazepam | C19H17ClN2O | pos | 342.134296 |
| 5-Nitro-2-(3-phenylpropylamino)benzoic acid | C16H16N2O4 | pos | 301.1184844 |
| Blumealactone C | C17H24O6 | pos | 342.1917491 |
| 5-Hydroxydecanedioylcarnitine | C17H31NO7 | pos | 326.1970462 |
| N-Eicosapentaenoyl Phenylalanine | C29H39NO3 | pos | 488.2551066 |
| EPIMESTROL | C19H26O3 | pos | 347.1606822 |
| Gamma-Glu-leu | C11H20N2O5 | pos | 261.1449053 |
| Oxybutynin AL | C5H2O2 | pos | 206.0450227 |
| Kresoxim-Methyl | C18H19NO4 | pos | 331.1658157 |
| Ethofumesate-2-Keto | C11H12O5S | pos | 274.0746233 |
| Delphinidin | C15H11O7+ | pos | 326.038079 |
| Indan-1-ol | C9H10O | pos | 117.0702084 |
| 3-Hydroxydodec-8-enedioylcarnitine | C19H33NO7 | pos | 370.223109 |
| N-(5-acetamidopentyl)acetamide | C9H18N2O2 | pos | 187.1444074 |
| Gamma-Glutamylvaline | C10H18N2O5 | pos | 247.1290728 |
| 1,5-Naphthalenediamine | C10H10N2 | pos | 159.0917801 |
| 3-Hydroxy-2-oxoindole | C8H7NO2 | pos | 132.0447472 |
| N-Acetylputrescine | C6H14N2O | pos | 131.1181751 |
| Cytosine | C4H5N3O | pos | 112.0509639 |
| 2,7-dihydroxy-4'-methoxyisoflavanone | C16H14O5 | pos | 287.091941 |
| N-(2-methoxy-5-methylphenyl)-2,5-dimethyl-1,3-oxazole-4-carboxamide | C14H16N2O3 | pos | 261.1236443 |
| Cyanidin 3-O-alpha-L-arabinoside | C20H19O10+ | neg | 400.0834516 |
| Griseolic acid | C14H13N5O8 | neg | 414.0420566 |
| 4-Ethylphenol | C8H10O | neg | 121.0653416 |
| Sinensetin | C20H20O7 | neg | 353.1012264 |
| Riboprine | C15H21N5O4 | neg | 372.1095078 |
| P-Tolyl Sulfate | C7H8O4S | neg | 187.0066985 |
| O-methoxycatechol-O-sulphate | C7H8O5S | neg | 203.0017215 |
| Gibberellin A37 | C20H26O5 | neg | 345.1727241 |
| THURFYL NICOTINATE | C11H13NO3 | neg | 206.0820813 |
| Glycerophosphoinositol | C9H19O11P | neg | 333.0622952 |
| Dihydrowogonin 7-glucoside | C22H24O10 | neg | 447.1309116 |
| 3-Methoxy-4-hydroxyphenylglycol glucuronide | C15H20O10 | neg | 341.0883764 |
| Dihydrokaempferol | C15H12O6 | neg | 287.0567251 |
| Phenol sulphate | C6H6O4S | neg | 172.9910371 |
| Urothion | C11H11N5O3S2 | neg | 324.0235588 |
| Mono-(2-ethyl-5-hydroxyhexyl) phthalate | C16H22O5 | neg | 587.2892161 |
| S-Glutaryldihydrolipoamide | C13H23NO4S2 | neg | 366.1061143 |
| Aminocatechol N-acetate sulfate | C8H9NO6S | neg | 227.9972751 |
| 7-Methylguanosine 5'-phosphate | C11H17N5O8P+ | neg | 377.0707954 |
| 1-Methyluric acid | C6H6N4O3 | neg | 181.0363903 |
| Dihydro-3-coumaric acid | C9H10O3 | neg | 165.0552939 |
| (E)-8-(2-(4-(Diphenylamino)benzylidene)hydrazinyl)-N-hydroxy-8-oxooctanamide | C27H30N4O3 | neg | 495.1846578 |
| Cyclopropanecarboxamide, 1,2,2,3,3-pentamethyl- | C9H17NO | neg | 200.1289692 |
| (2R,4S)-4-Carbamimidamido-3-acetamido-2-((1R,2R)-2-hydroxy-1-methoxy-3-(octanoyloxy)propyl)-3,4-dihydro-2H-pyran-6-carboxylic acid | C21H36N4O8 | neg | 471.2429239 |
| Amastatin | C21H38N4O8 | neg | 473.2593038 |
| 3-Methoxyphenol sulfate | C7H8O5S | neg | 249.0077494 |
| Indole carboxylic acid sulfate | C9H7NO5S | neg | 239.9973463 |
| Lersivirine | C17H18N4O2 | neg | 291.1277234 |
| 4-Vinylphenol sulfate | C8H8O4S | neg | 245.0127382 |
| P-Tolyl-ss-D-glucuronide | C13H16O7 | neg | 283.0828416 |
| Cis-Melilotoside | C15H18O8 | neg | 325.0937842 |
| 2-Methoxyacetaminophen sulfate | C9H11NO6S | neg | 242.013053 |
| [4-[4-(4-Hydroxyphenyl)hex-3-en-3-yl]phenyl] phosphono hydrogen phosphate | C18H22O8P2 | neg | 409.0608396 |
| LysoPC(14:1(9Z)/0:0) | C22H44NO7P | neg | 486.2580132 |
| Methylthiomethyl hexanoate | C8H16O2S | neg | 221.0853476 |
| N-[4-Cloro-3-(T-butyloxome)phenyl-2-methyl-3-furan-carbothiamide | C17H19ClN2O2S | neg | 395.0813295 |
| 4-Vinylphenol | C8H8O | neg | 119.049691 |
| Melibiitol | C12H24O11 | neg | 381.0770589 |
| [(S)-1-Carboxy-2-phenyl-ethyl]-carbamoyl-Arg-Val-Arg-aldehyde | C27H44N10O6 | neg | 625.3225471 |
| Glycosminine | C15H12N2O | neg | 273.0443476 |
| Phenyl salicylate | C13H10O3 | neg | 259.0615443 |
| Hydroxypioglitazone | C19H20N2O4S | neg | 407.0814637 |
| Geranyl-PP | C10H20O7P2 | neg | 351.0185666 |
| Austin | C27H32O9 | neg | 499.2016325 |
| Cambendazole | C14H14N4O2S | neg | 323.0597817 |
| Ichangin 4-glucoside | C32H42O14 | neg | 631.2427729 |
| Pyroglutamyl-prolyl-arginine-4-nitroanilide | C22H30N8O6 | neg | 483.2065498 |
| PE(15:1/0:0) | C20H40NO7P | neg | 436.2477208 |
| Sambacin | C26H36O12 | neg | 575.1923802 |
| Polyporenic acid C | C31H46O4 | neg | 519.2927866 |
| Atorvastatin lactone | C33H33FN2O4 | neg | 561.2131666 |
| 7-O-Succinyl macrolactin A | C28H38O8 | neg | 483.2421726 |
| Cholic Acid | C24H40O5 | neg | 815.5706096 |
| 7-Hydroxyhexadecanedioylcarnitine | C23H43NO7 | neg | 482.2537552 |
| Gibberellin A38 glucosyl ester | C26H36O11 | neg | 559.1972432 |
| Physagulin E | C36H50O14 | neg | 743.2692875 |
| VPGPR Enterostatin | C23H40N8O6 | neg | 545.2793189 |
| 4-Hydroxy-2,6,6-trimethyl-3-oxo-1,4-cyclohexadiene-1-carboxaldehyde | C10H12O3 | neg | 539.2311709 |
| PC(18:1(11Z)/18:3(9Z,12Z,15Z)) | C44H80NO8P | neg | 762.5372927 |
| 3-Hydroxyhexadecanedioylcarnitine | C23H43NO7 | neg | 482.2531946 |
| Pinometostat | C30H42N8O3 | neg | 599.288913 |
| 4-(2-Aminoethyl)cyclohexa-3,5-diene-1,2-diol | C8H13NO2 | neg | 464.2787872 |
| Bazedoxifene | C30H34N2O3 | neg | 507.2043104 |
| Cinncassiol C1 19-glucoside | C26H38O12 | neg | 577.2080358 |
| 2,3-dinor-8-iso-PGF2a | C18H30O5 | neg | 325.1999333 |
| 3-[4-(sulfooxy)phenyl]propanoic acid | C9H10O6S | neg | 245.0128214 |
| [(3R,4S)-1,1-Difluoro-3-(hexadecanoylamino)-4-hydroxy-4-phenylbutyl]phosphonic acid | C26H44F2NO5P | neg | 500.2695543 |
| 3-Dehydrocholic Acid | C24H38O5 | neg | 811.5392314 |
| 3-Hydroxybenzyl alcohol glucoside | C13H18O7 | neg | 285.0997999 |
| Cinobufagin | C26H34O6 | neg | 487.2373862 |
| Dodecyl-beta-D-maltoside | C24H46O11 | neg | 555.3004831 |
| (-)-Naringenin | C15H12O5 | neg | 271.0615629 |
| (S)-Equol | C15H14O3 | neg | 241.0875293 |
| Urobilin | C33H42N4O6 | neg | 589.3046824 |
| Pantetheine 4'-phosphate | C11H23N2O7PS | neg | 393.0657539 |
| Avatrombopag | C29H34Cl2N6O3S2 | neg | 323.0678651 |
| Vulgarin | C15H20O4 | neg | 587.2892042 |
| Hallacridone | C18H13NO4 | neg | 288.066426 |
| N-[(3a,5b,7a)-3-hydroxy-24-oxo-7-(sulfooxy)cholan-24-yl]-Glycine | C26H43NO8S | neg | 528.2647723 |
| Padmatin | C16H14O7 | neg | 317.0670481 |
| Pglu-his-pro | C16H21N5O5 | neg | 398.1252697 |
| Sakuranin | C22H24O10 | neg | 493.1366232 |
| Stercobilinogen | C33H48N4O6 | pos | 597.366015 |
| PE(PGF1alpha/20:4(8Z,11Z,14Z,17Z)) | C45H78NO11P | pos | 862.519963 |
| Pseudouridine | C9H12N2O6 | pos | 245.0771178 |
| Pymetrozine | C10H11N5O | pos | 218.1026279 |
| Val Val Glu | C15H27N3O6 | pos | 346.1981359 |
| Arginylisoleucine | C12H25N5O3 | pos | 270.1928282 |
| Levamisole | C11H12N2S | pos | 472.1591443 |
| Neopterin | C9H11N5O4 | pos | 276.0693871 |
| N-Acetyltryptophan | C13H14N2O3 | pos | 229.0974094 |
| Oleoyltaurine | C20H39NO4S | pos | 372.2600681 |
| 5,8-Dihydroxy-1,4-naphthoquinone | C10H6O4 | pos | 208.0607683 |
| 1-Benzylpiperidine-4-carboxylic acid | C13H17NO2 | pos | 202.1229318 |
| 20-Cooh ltb4 | C21H32O6 | pos | 425.1897629 |
| Indole-3-Carboxaldehyde | C9H7NO | pos | 146.0602015 |
| (1R,2S,7R,9R,10R,12R)-10-Hydroxy-2-(hydroxymethyl)-1,5-dimethylspiro[8-oxatricyclo[7.2.1.02,7]dodecane-12,2'-oxirane]-3,4-dione | C15H20O6 | pos | 297.1336942 |
| Methyl (3x,4E,10R)-3,10-dihydroxy-4,11-dodecadiene-6,8-diynoate 10-glucoside | C19H24O9 | pos | 419.1291952 |
| Dimethylallylpyrophosphate | C5H12O7P2 | pos | 310.020703 |
| 1,1,1-Trifluorohenicosa-6,9,12,15-tetraene | C21H33F3 | pos | 387.2254407 |
| (2R,3R,4R,5S)-2-(Hydroxymethyl)-1-nonylpiperidine-3,4,5-triol | C15H31NO4 | pos | 620.4880204 |
| Soyasapogenol C | C30H48O2 | pos | 504.3780471 |
| 14-demethyllanosterol | C29H46O | pos | 433.3409032 |
| LysoPG(18:1(9Z)/0:0) | C24H47O9P | pos | 533.2812541 |
| Atractylenolide II | C15H20O2 | pos | 506.3251089 |
| Porphobilinogen | C10H14N2O4 | pos | 470.2263325 |
| Deoxycortisol | C21H32O4 | pos | 381.2621638 |
| Cardanolmonoene | C21H34O | pos | 325.2528629 |
| Alpha-Dimorphecolic acid | C18H32O3 | pos | 261.2214698 |
| 2,6-Dimethylaniline | C8H11N | pos | 122.0967646 |
| LysoSM(d18:1) | C23H50N2O5P+ | pos | 510.3194527 |
| (R)-Pronuciferine | C19H21NO3 | pos | 623.3091789 |
| Formiminoglutamic acid | C6H10N2O4 | pos | 175.0716026 |
| Mycorradicin | C14H16O4 | pos | 281.1387105 |
| Val-Phe-Val-Tyr | C28H38N4O6 | pos | 559.3105146 |
| Delapril | C26H32N2O5 | pos | 470.2661524 |
| Gentamicin A sulfate | C18H36N4O10 | pos | 486.2727164 |
| Fluvoxamine | C15H21F3N2O2 | pos | 637.324302 |
| 3-Methylindole | C9H9N | pos | 132.0810283 |
| Aspartyl-Tryptophan | C15H17N3O5 | pos | 284.1051955 |
| Gamma-Glutamylglutamic acid | C10H16N2O7 | pos | 299.0854016 |
| Cowagarcinone D | C28H30O6 | pos | 445.1983441 |
| N-Hydroxy-L-tyrosine | C9H11NO4 | pos | 162.055096 |
| Netilmicin | C21H41N5O7 | pos | 476.3077643 |
| Entacapone | C14H15N3O5 | pos | 270.0895457 |
| Methionyl-Glycine | C7H14N2O3S | pos | 270.0896172 |
| Tirofiban | C22H36N2O5S | pos | 458.2659933 |
| Dehydrosafynol | C13H10O2 | pos | 221.0560156 |
| 3-Hydroxy-cis-5-octenoylcarnitine | C15H27NO5 | pos | 302.1969607 |
| 5-(2'-Carboxyethyl)-4,6-Dihydroxypicolinate | C9H9NO6 | pos | 260.0743917 |
| Flavin adenine dinucleotide (FAD) | C27H33N9O15P2 | pos | 786.167063 |
| 4-(2-Amino-3-hydroxyphenyl)-2,4-dioxobutanoic acid | C10H9NO5 | pos | 206.0451168 |
| Panthenol | C9H19NO4 | pos | 206.1390437 |
| Nivalenol | C15H20O7 | pos | 354.1529955 |
| Glucose propionate | C9H16O8 | pos | 217.0709842 |
| Ribothymidine | C10H14N2O6 | pos | 259.0928657 |
| Mefloquine | C17H16F6N2O | pos | 379.1219171 |
| ARGININOSUCCINATE | C10H18N4O6 | pos | 335.095444 |
| Ile Leu | C12H24N2O3 | pos | 245.1863045 |
| 4-Methyl-5-Thiazoleethanol | C6H9NOS | pos | 144.047922 |
| Thiamine | C12H17N4OS+ | pos | 265.1119775 |
| Coumafuryl | C17H14O5 | neg | 343.0828189 |
| Gossyvertin | C16H18O4 | neg | 273.1138007 |
| 5-Hydroxyindoxyl sulfate | C9H9NO3S | neg | 210.0228889 |
| Pantetheine | C11H22N2O4S | neg | 277.1231178 |
| Fosphenytoin | C16H15N2O6P | neg | 343.0494339 |
| Fasoracetam | C10H16N2O2 | neg | 241.1194856 |
| TWS119 | C18H14N4O2 | neg | 317.1040611 |
| 2-Hydroxybenzaldehyde | C7H6O2 | neg | 121.0289923 |
| 2,8-Quinolinediol | C9H7NO2 | neg | 206.0457324 |
| Ascorbigen | C15H15NO6 | neg | 342.041502 |
| (E)-8-Hydroxy-2-octene-4,6-diynoic acid | C8H6O3 | neg | 359.0784069 |
| Vanillic acid 4-O-sulfate | C8H8O7S | neg | 228.9809292 |
| Theobromine | C7H8N4O2 | neg | 161.0479579 |
| P-Toluenesulfonic Acid | C7H8O3S | neg | 171.0117121 |
| 3-(3-Oxobutanoyloxy)butanoic acid | C8H12O5 | neg | 223.0377266 |
| N-[(3s)-2-Oxotetrahydrofuran-3-Yl]butanamide | C8H13NO3 | neg | 216.0877624 |
| 10-Formyldihydrofolate | C20H21N7O7 | neg | 470.1442292 |
| (2R,2'R)-3,3'-disulfanediylbis(2-acetamidopropanoic acid) | C10H16N2O6S2 | neg | 323.0381972 |
| Xanthosine | C10H12N4O6 | neg | 283.0689742 |
| 5-Hydroxy-N-formylkynurenine | C11H12N2O5 | neg | 251.06843 |
| Inosine | C10H12N4O5 | neg | 267.0740713 |
| 1,4-Benzothiazine-O-quinonimine | C12H12N2O5S | neg | 332.9926 |
| (R)-5-Diphosphomevalonic acid | C6H14O10P2 | neg | 328.9835414 |
| 3,5-Dichloro-L-tyrosine | C9H9Cl2NO3 | neg | 285.9428126 |
| Oxoglutaric acid | C5H6O5 | neg | 145.0137856 |
| Glucose pyruvate | C9H14O9 | neg | 265.0569666 |
| Gentiobioside | C13H24O11 | neg | 391.1023644 |
| 9,10-Epoxyoctadecanoic acid | C18H34O4 | neg | 313.2388469 |
| Isoursodeoxycholic acid | C24H40O4 | neg | 413.2681118 |
| Ethyl hydrogen sulfate | C2H6O4S | neg | 170.9964494 |
| 1-Methylpseudouridine | C10H14N2O6 | neg | 293.0552006 |
| 3,4-Methylenedioxybenzaldehyde | C8H6O3 | neg | 149.0239485 |
| S-Carboxymethyl-L-cysteine | C5H9NO4S | neg | 215.9730253 |
| Succinic Anhydride | C4H4O3 | neg | 99.00810694 |
| 4-Methylthio-2-Oxobutanoic Acid | C5H8O3S | neg | 147.011669 |
| Xanthurenic Acid | C10H7NO4 | neg | 204.0300846 |
| 3,4-dihydroxyphenylacetic Acid | C8H8O4 | neg | 167.0346458 |
| Toluene diisocyanate | C9H6N2O2 | neg | 219.0410614 |
| P-Acetaminobenzaldehyde | C9H9NO2 | neg | 162.0556016 |
| Diacetyl benzene-1,2-dicarboxylate | C12H10O6 | neg | 231.0275752 |
| Neotussilagine | C10H17NO3 | neg | 244.1192241 |
| Floionolic acid | C18H36O5 | neg | 331.2492954 |
| PA(12:0/18:1(12Z)-2OH(9,10)) | C33H63O10P | neg | 685.3826558 |
| SM(d19:0/PGD2) | C44H83N2O9P | neg | 849.5529723 |
| Cohibin B | C35H64O4 | neg | 593.4801539 |
| FAD | C27H33N9O15P2 | neg | 784.153472 |
| Trodusquemine | C37H72N4O5S | neg | 719.4972066 |
| SM(d19:0/20:4(6E,8Z,11Z,13E)-2OH(5S,15S)) | C44H83N2O8P | neg | 833.5583135 |
| SM(d16:1/5-iso PGF2VI) | C39H73N2O9P | neg | 781.4559194 |
| PA(18:1(12Z)-2OH(9,10)/a-13:0) | C34H65O10P | neg | 699.3982087 |
| N-Oleoyl Cysteine | C21H39NO3S | neg | 769.5286822 |
| Cholestane-3,7,12,25-tetrol-3-glucuronide | C33H56O10 | neg | 593.3708115 |
| Arginyl-glycyl-aspartic acid | C12H22N6O6 | neg | 345.1498627 |
| N-Lauroylglycine | C14H27NO3 | neg | 256.1919993 |
| Alpha-Bisabolol oxide A | C15H26O2 | neg | 283.1917202 |
| 3-hydroxypristanic acid | C19H38O3 | neg | 359.2807838 |
| (11S,12S,13S)-Epoxy-hydroxyoctadeca-cis-9-cis-15-dien-1-oic acid | C18H30O4 | neg | 331.1920731 |
| Phenylpyruvic acid | C9H8O3 | neg | 163.0395387 |
| 9,10,16-trihydroxy palmitic acid | C16H32O5 | neg | 303.2177835 |
| Subaphylline | C14H20N2O3 | neg | 573.293188 |
| Pantethine | C22H42N4O8S2 | neg | 553.2389378 |
| 3-[[5-Methyl-2-(1-methylethyl)cyclohexyl]oxy]-1,2-propanediol | C13H26O3 | neg | 275.1867617 |
| 4,4'-Sulfonyldiphenol | C12H10O4S | neg | 249.0229369 |

Notes: Metabolite: name of the identified metabolite; Formula: chemical formula of the metabolite; Mode: ionic mode, pos positive, neg negative mode; M/Z: mass-to-charge ratio, the ratio of the mass of a charged ion to its charge.

**Supplementary Table 9. Callback fecal metabolites of EA and MA**

| Metabolite | Formula | Mode | M/Z |
| --- | --- | --- | --- |
| PE(PGF1alpha/20:4(8Z,11Z,14Z,17Z)) | C45H78NO11P | pos | 862.519963 |
| N,N'-Di-1,2,3,4-Tetrahydroacridin-9-Ylheptane-1,7-Diamine | C33H40N4 | pos | 510.3564288 |
| Benzotest | C26H38O3 | pos | 381.2793864 |
| Dihydroceramide | C19H39NO3 | pos | 330.3006232 |
| Pseudouridine | C9H12N2O6 | pos | 245.0771178 |
| 5-NITRO-2-PHENYLPROPYLAMINOBENZOIC ACID [NPPB] | C16H16N2O4 | pos | 301.1184075 |
| Cilengitide | C27H40N8O7 | pos | 589.3037542 |
| Avadomide | C14H14N4O3 | pos | 287.1143086 |
| Ethenoadenosine | C12H13N5O4 | pos | 274.0926619 |
| 7-Hydroxy-8-O-methylaloin B | C22H24O10 | pos | 471.1277318 |
| Linalyl propionate | C13H22O2 | pos | 175.1483306 |
| Levamisole | C11H12N2S | pos | 472.1591443 |
| Valganciclovir, (S)- | C14H22N6O5 | pos | 319.1520722 |
| Oleoyltaurine | C20H39NO4S | pos | 372.2600681 |
| Kinetensin 4-7 | C26H37N9O6 | pos | 613.3251599 |
| Piperdial | C15H22O3 | pos | 289.1187255 |
| 6-Keto-prostaglandin F1a | C20H34O6 | pos | 370.2346065 |
| 5,8-Dihydroxy-1,4-naphthoquinone | C10H6O4 | pos | 208.0607683 |
| 1-Benzylpiperidine-4-carboxylic acid | C13H17NO2 | pos | 202.1229318 |
| 20-Cooh ltb4 | C21H32O6 | pos | 425.1897629 |
| 7-Hydroxy-6-(methoxyacetyl)-2,2-dimethyl-2H-1-benzopyran | C14H16O4 | pos | 249.1123427 |
| Cervonoyl ethanolamide | C24H36O3 | pos | 355.2635075 |
| 5'-Deoxy-5-fluorocytidine | C9H12FN3O4 | pos | 287.1141905 |
| Indole-3-Carboxaldehyde | C9H7NO | pos | 146.0602015 |
| DG(14:1(9Z)/16:1(9Z)/0:0) | C33H60O5 | pos | 536.4437614 |
| Melanettin | C16H12O5 | pos | 302.1027912 |
| 1-{2-[(3-Ethylphenyl)amino]-2-oxoethyl}-6-oxo-1,6-dihydropyridine-3-carboxylic acid | C16H16N2O4 | pos | 301.1186407 |
| (5Z,7S,8E,10Z,13Z,15E,17S,19Z)-7,17-Dihydroxydocosa-5,8,10,13,15,19-hexaenoylcarnitine | C29H45NO6 | pos | 542.2907794 |
| Fluvoxamino acid | C14H17F3N2O3 | pos | 301.1187163 |
| Tragopogonsaponin J | C57H84O22 | pos | 583.2566936 |
| 1,1,1-Trifluorohenicosa-6,9,12,15-tetraene | C21H33F3 | pos | 387.2254407 |
| 7-a,27-Dihydroxycholesterol | C27H46O3 | pos | 401.3420682 |
| Phenacyl 5-[(3aR,4S,5R,6aR)-5-hydroxy-4-[(3R,4S)-3-hydroxy-4-methyloct-1-en-6-ynyl]-3,3a,4,5,6,6a-hexahydro-1H-pentalen-2-ylidene]pentanoate | C30H38O5 | pos | 461.273246 |
| Soyasapogenol C | C30H48O2 | pos | 504.3780471 |
| DG(2:0/0:0/PGD1) | C25H42O8 | pos | 471.2935038 |
| LysoPG(18:1(9Z)/0:0) | C24H47O9P | pos | 533.2812541 |
| Porphobilinogen | C10H14N2O4 | pos | 470.2263325 |
| Nonadecanoic acid | C19H38O2 | pos | 316.3212835 |
| PC(20:2(11Z,14Z)/TXB2) | C48H86NO12P | pos | 922.5723274 |
| Alpha-Dimorphecolic acid | C18H32O3 | pos | 261.2214698 |
| 4-Hydroxyestrone-2-S-glutathione | C28H37N3O9S | pos | 633.2570703 |
| N-Eicosapentaenoyl Arginine | C26H42N4O3 | pos | 500.3594512 |
| (2xi,4xi)-2,4-Nonadien-1-ol | C9H16O | pos | 158.1540954 |
| Deoxyspergualin | C17H37N7O3 | pos | 370.2957044 |
| Estrone | C18H22O2 | pos | 315.134187 |
| DG(8:0/i-19:0/0:0) | C30H58O5 | pos | 537.3912696 |
| Citpressine II | C17H17NO5 | pos | 333.1446927 |
| Metoprolol | C15H25NO3 | pos | 535.3756039 |
| DG(8:0/19:0/0:0) | C30H58O5 | pos | 537.3914475 |
| DG(16:1(9Z)/14:1(9Z)/0:0) | C33H60O5 | pos | 536.4437862 |
| (2,5-Dihydroxypyrrol-1-yl) 4-[(2,5-dioxopyrrol-1-yl)methyl]cyclohexane-1-carboxylate | C16H18N2O6 | pos | 379.0852452 |
| N-Acetylneuraminic Acid | C11H19NO9 | pos | 310.113847 |
| Urobilinogen | C33H42N4O6 | pos | 613.301268 |
| W Hydroxy testosterone | C19H28O3 | pos | 343.1653141 |
| 5-p-Coumaroylquinic acid | C16H18O8 | pos | 339.1082304 |
| Val-Phe-Val-Tyr | C28H38N4O6 | pos | 559.3105146 |
| Dolichyl b-D-glucosyl phosphate | C21H39O9P | pos | 466.2347361 |
| (3-Aminopropyl)(n-butyl)phosphinic acid | C7H18NO2P | pos | 144.0935289 |
| (4As,5aR,6R,12aR)-1,10,11,12a-tetrahydroxy-6-methyl-3,12-dioxo-4a,5,5a,6-tetrahydro-4H-tetracene-2-carboxamide | C20H19NO7 | pos | 386.12438 |
| Prazepam | C19H17ClN2O | pos | 342.134296 |
| Gamma-Glutamylglutamic acid | C10H16N2O7 | pos | 299.0854016 |
| 5-Hydroxydecanedioylcarnitine | C17H31NO7 | pos | 326.1970462 |
| N-Hydroxy-L-tyrosine | C9H11NO4 | pos | 162.055096 |
| Netilmicin | C21H41N5O7 | pos | 476.3077643 |
| N-Eicosapentaenoyl Phenylalanine | C29H39NO3 | pos | 488.2551066 |
| EPIMESTROL | C19H26O3 | pos | 347.1606822 |
| Dehydrosafynol | C13H10O2 | pos | 221.0560156 |
| Panthenol | C9H19NO4 | pos | 206.1390437 |
| Nivalenol | C15H20O7 | pos | 354.1529955 |
| Mefloquine | C17H16F6N2O | pos | 379.1219171 |
| ARGININOSUCCINATE | C10H18N4O6 | pos | 335.095444 |
| 4-Methyl-5-Thiazoleethanol | C6H9NOS | pos | 144.047922 |
| Thiamine | C12H17N4OS+ | pos | 265.1119775 |
| 2,7-dihydroxy-4'-methoxyisoflavanone | C16H14O5 | pos | 287.091941 |
| Coumafuryl | C17H14O5 | neg | 343.0828189 |
| Gossyvertin | C16H18O4 | neg | 273.1138007 |
| Cyanidin 3-O-alpha-L-arabinoside | C20H19O10+ | neg | 400.0834516 |
| Riboprine | C15H21N5O4 | neg | 372.1095078 |
| P-Tolyl Sulfate | C7H8O4S | neg | 187.0066985 |
| Fasoracetam | C10H16N2O2 | neg | 241.1194856 |
| O-methoxycatechol-O-sulphate | C7H8O5S | neg | 203.0017215 |
| TWS119 | C18H14N4O2 | neg | 317.1040611 |
| Gibberellin A37 | C20H26O5 | neg | 345.1727241 |
| Glycerophosphoinositol | C9H19O11P | neg | 333.0622952 |
| Dihydrowogonin 7-glucoside | C22H24O10 | neg | 447.1309116 |
| 3-Methoxy-4-hydroxyphenylglycol glucuronide | C15H20O10 | neg | 341.0883764 |
| (E)-8-Hydroxy-2-octene-4,6-diynoic acid | C8H6O3 | neg | 359.0784069 |
| Dihydrokaempferol | C15H12O6 | neg | 287.0567251 |
| Theobromine | C7H8N4O2 | neg | 161.0479579 |
| P-Toluenesulfonic Acid | C7H8O3S | neg | 171.0117121 |
| Phenol sulphate | C6H6O4S | neg | 172.9910371 |
| 3-(3-Oxobutanoyloxy)butanoic acid | C8H12O5 | neg | 223.0377266 |
| Mono-(2-ethyl-5-hydroxyhexyl) phthalate | C16H22O5 | neg | 587.2892161 |
| 10-Formyldihydrofolate | C20H21N7O7 | neg | 470.1442292 |
| Aminocatechol N-acetate sulfate | C8H9NO6S | neg | 227.9972751 |
| 7-Methylguanosine 5'-phosphate | C11H17N5O8P+ | neg | 377.0707954 |
| 5-Hydroxy-N-formylkynurenine | C11H12N2O5 | neg | 251.06843 |
| Cyclopropanecarboxamide, 1,2,2,3,3-pentamethyl- | C9H17NO | neg | 200.1289692 |
| Glucose pyruvate | C9H14O9 | neg | 265.0569666 |
| Gentiobioside | C13H24O11 | neg | 391.1023644 |
| (2R,4S)-4-Carbamimidamido-3-acetamido-2-((1R,2R)-2-hydroxy-1-methoxy-3-(octanoyloxy)propyl)-3,4-dihydro-2H-pyran-6-carboxylic acid | C21H36N4O8 | neg | 471.2429239 |
| 9,10-Epoxyoctadecanoic acid | C18H34O4 | neg | 313.2388469 |
| Amastatin | C21H38N4O8 | neg | 473.2593038 |
| Indole carboxylic acid sulfate | C9H7NO5S | neg | 239.9973463 |
| 3,4-dihydroxyphenylacetic Acid | C8H8O4 | neg | 167.0346458 |
| Toluene diisocyanate | C9H6N2O2 | neg | 219.0410614 |
| Lersivirine | C17H18N4O2 | neg | 291.1277234 |
| Cis-Melilotoside | C15H18O8 | neg | 325.0937842 |
| 2-Methoxyacetaminophen sulfate | C9H11NO6S | neg | 242.013053 |
| LysoPC(14:1(9Z)/0:0) | C22H44NO7P | neg | 486.2580132 |
| Methylthiomethyl hexanoate | C8H16O2S | neg | 221.0853476 |
| N-[4-Cloro-3-(T-butyloxome)phenyl-2-methyl-3-furan-carbothiamide | C17H19ClN2O2S | neg | 395.0813295 |
| Neotussilagine | C10H17NO3 | neg | 244.1192241 |
| [(S)-1-Carboxy-2-phenyl-ethyl]-carbamoyl-Arg-Val-Arg-aldehyde | C27H44N10O6 | neg | 625.3225471 |
| Glycosminine | C15H12N2O | neg | 273.0443476 |
| Phenyl salicylate | C13H10O3 | neg | 259.0615443 |
| Hydroxypioglitazone | C19H20N2O4S | neg | 407.0814637 |
| Austin | C27H32O9 | neg | 499.2016325 |
| Cambendazole | C14H14N4O2S | neg | 323.0597817 |
| Floionolic acid | C18H36O5 | neg | 331.2492954 |
| PA(12:0/18:1(12Z)-2OH(9,10)) | C33H63O10P | neg | 685.3826558 |
| SM(d19:0/PGD2) | C44H83N2O9P | neg | 849.5529723 |
| Sambacin | C26H36O12 | neg | 575.1923802 |
| Atorvastatin lactone | C33H33FN2O4 | neg | 561.2131666 |
| 7-O-Succinyl macrolactin A | C28H38O8 | neg | 483.2421726 |
| Cholic Acid | C24H40O5 | neg | 815.5706096 |
| Gibberellin A38 glucosyl ester | C26H36O11 | neg | 559.1972432 |
| 4-Hydroxy-2,6,6-trimethyl-3-oxo-1,4-cyclohexadiene-1-carboxaldehyde | C10H12O3 | neg | 539.2311709 |
| PC(18:1(11Z)/18:3(9Z,12Z,15Z)) | C44H80NO8P | neg | 762.5372927 |
| Pinometostat | C30H42N8O3 | neg | 599.288913 |
| Bazedoxifene | C30H34N2O3 | neg | 507.2043104 |
| Cinncassiol C1 19-glucoside | C26H38O12 | neg | 577.2080358 |
| PA(18:1(12Z)-2OH(9,10)/a-13:0) | C34H65O10P | neg | 699.3982087 |
| N-Oleoyl Cysteine | C21H39NO3S | neg | 769.5286822 |
| Cholestane-3,7,12,25-tetrol-3-glucuronide | C33H56O10 | neg | 593.3708115 |
| Arginyl-glycyl-aspartic acid | C12H22N6O6 | neg | 345.1498627 |
| N-Lauroylglycine | C14H27NO3 | neg | 256.1919993 |
| [(3R,4S)-1,1-Difluoro-3-(hexadecanoylamino)-4-hydroxy-4-phenylbutyl]phosphonic acid | C26H44F2NO5P | neg | 500.2695543 |
| 3-Dehydrocholic Acid | C24H38O5 | neg | 811.5392314 |
| 3-hydroxypristanic acid | C19H38O3 | neg | 359.2807838 |
| (11S,12S,13S)-Epoxy-hydroxyoctadeca-cis-9-cis-15-dien-1-oic acid | C18H30O4 | neg | 331.1920731 |
| 3-Hydroxybenzyl alcohol glucoside | C13H18O7 | neg | 285.0997999 |
| Cinobufagin | C26H34O6 | neg | 487.2373862 |
| Dodecyl-beta-D-maltoside | C24H46O11 | neg | 555.3004831 |
| (-)-Naringenin | C15H12O5 | neg | 271.0615629 |
| 9,10,16-trihydroxy palmitic acid | C16H32O5 | neg | 303.2177835 |
| Subaphylline | C14H20N2O3 | neg | 573.293188 |
| Pantethine | C22H42N4O8S2 | neg | 553.2389378 |
| 3-[[5-Methyl-2-(1-methylethyl)cyclohexyl]oxy]-1,2-propanediol | C13H26O3 | neg | 275.1867617 |
| (S)-Equol | C15H14O3 | neg | 241.0875293 |
| Urobilin | C33H42N4O6 | neg | 589.3046824 |
| Pantetheine 4'-phosphate | C11H23N2O7PS | neg | 393.0657539 |
| Avatrombopag | C29H34Cl2N6O3S2 | neg | 323.0678651 |
| Vulgarin | C15H20O4 | neg | 587.2892042 |
| 4,4'-Sulfonyldiphenol | C12H10O4S | neg | 249.0229369 |
| Hallacridone | C18H13NO4 | neg | 288.066426 |
| N-[(3a,5b,7a)-3-hydroxy-24-oxo-7-(sulfooxy)cholan-24-yl]-Glycine | C26H43NO8S | neg | 528.2647723 |
| Padmatin | C16H14O7 | neg | 317.0670481 |
| Pglu-his-pro | C16H21N5O5 | neg | 398.1252697 |
| Sakuranin | C22H24O10 | neg | 493.1366232 |

Notes: Metabolite: name of the identified metabolite; Formula: chemical formula of the metabolite; Mode: ionic mode, pos positive, neg negative mode; M/Z: mass-to-charge ratio, the ratio of the mass of a charged ion to its charge.
